# Randomised Trial of No, Short-term, or Long-term Androgen Deprivation Therapy with Postoperative Radiotherapy After Radical Prostatectomy: Results from the Three-way Comparison of RADICALS-HD (NCT00541047)

**DOI:** 10.1016/j.eururo.2024.07.026

**Published:** 2024-08-31

**Authors:** Chris C. Parker, Noel W. Clarke, Adrian D. Cook, Peter M. Petersen, Charles N. Catton, William R. Cross, Howard Kynaston, Raj A. Persad, Fred Saad, John Logue, Heather Payne, Claire Amos, Lorna Bower, Rakesh Raman, Ian Sayers, Jane Worlding, Wendy R. Parulekar, Mahesh K.B. Parmar, Matthew R. Sydes, RADICALS Investigators

**Affiliations:** a Royal Marsden NHS Foundation Trust, Sutton, UK; b The Institute of Cancer Research, Sutton, UK; c Department of Urology, The Christie and Salford Royal Hospitals, Manchester, UK; d The University of Manchester, Manchester, UK; e MRC Clinical Trials Unit at UCL, Institute of Clinical Trials and Methodology, UCL, London, UK; f Department of Oncology, Rigshospitalet, University of Copenhagen, Copenhagen, Denmark; g Department of Radiation Oncology, Princess Margaret, Cancer Centre, University Health Network, Toronto, ON, Canada; h Department of Urology, St James’s University Hospital, Leeds, UK; i Division of Cancer & Genetics, Cardiff University Medical School, Cardiff, UK; j Department of Urology, Bristol Urological Institute, Bristol, UK; k Department of Urology, Centre Hospitalier de l’Université de Montréal, Montreal, Canada; l Christie Hospital, Manchester, UK; m The Prostate Centre, London, UK; n Guy’s and St Thomas’ NHS Foundation Trust, London, UK; o Kent Oncology Centre, Kent & Canterbury Hospital, Canterbury, UK; p Deanesly Centre, New Cross Hospital, Wolverhampton, UK; q University Hospitals Coventry and Warwickshire NHS Trust, Coventry, UK; r Canadian Cancer Trials Group, Queen’s University, Kingston, ON, Canada

**Keywords:** Prostate cancer, Randomised controlled trial, Clinical trials, Multiarm trial, Radical prostatectomy

## Abstract

**Background and objective:**

The use and duration of androgen deprivation therapy (ADT) with postoperative radiotherapy (RT) have been uncertain. RADICALS-HD compared adding no (“None”), 6-months (“Short”), or 24-mo (“Long”) ADT to study efficacy in the long term.

**Methods:**

Participants with prostate cancer were indicated for postoperative RT and agreed randomisation between all durations. ADT was allocated for 0, 6, or 24 mo. The primary outcome measure (OM) was metastasis-free survival (MFS). The secondary OMs included freedom from distant metastasis, overall survival, and initiation of non-protocol ADT. Sample size was determined by two-way comparisons. Analyses followed standard time-to-event approaches and intention-to-treat principles.

**Key findings and limitations:**

Between 2007 and 2015, 492 participants were randomised one of three groups: 166 None, 164 Short, and 162 Long. The median age at randomisation was 66 yr; Gleason scores at surgery were as follows: <7 = 64 (13%), 3+4 = 229 (47%), 4+3 = 127 (26%), and 8+ = 72 (15%); T3b was 112 (23%); and T4 was 5 (1%). The median follow-up was 9.0 yr and, with MFS events reported for 89 participants (32 None, 31 Short, and 26 Long), there was no evidence of difference in MFS overall (logrank *p* = 0.98), and, for Long versus None, hazard ratio = 0.948 (95% confidence interval 0.54–1.68). After 10 yr, 80% None, 77% Short, and 81% Long patients were alive without metastatic disease. The three-way randomisation was not powered to conventional levels for assessment, yet provides a fair comparison.

**Conclusions and clinical implications:**

Long-term outcomes after radical prostatectomy are usually favourable. In those indicated for postoperative RT and considered suitable for no, short-term, or long-term ADT, there was no evidence of improvement with addition of ADT. Future research should focus on patients at a higher risk of metastases in whom improvements are required more urgently.

## 1 Introduction

The RADICALS-HD trial tested the use and duration of androgen deprivation therapy (ADT) given in combination with postoperative radiotherapy (RT) after radical prostatectomy. Participants could be randomised to no ADT (here-after referred to as “None”), 6-months ADT (“Short”), or 24-months ADT (“Long”). How the trial design evolved has been described previously [1]. The results from RADICALS-HD were first presented at the European Society for Medical Oncology (ESMO) Annual Meeting in 2022 [2].

The analyses of RADICALS-HD have focused on the trial’s two main ADT comparisons (“None vs Short” and “Short vs Long”), which were each explicitly powered to detect a clinically relevant difference. The trial did not mandate randomisation between all three arms and, therefore, was not powered to compare RT alone versus RT plus 2 yr of ADT (“None vs Long”). The statistical analysis plan [3] included an analysis of that comparison for completeness and for contribution to wider analyses. These findings are presented here with reflection on the conduct of the trial, before discussing how the results might impact clinical practice and future research.

## 2 Patients and methods

Eligible patients had previously chosen surgery for their prostate cancer and were indicated for postoperative RT. According to the choice of participants and their physicians, RADICALS-HD participants could be randomised two-way None versus Short, two-way Short versus Long, or three-way None versus Short versus Long. Here, the three-way comparison of None versus Short versus Long, which is the only direct comparison of None versus Long, has been described. Participants in the three-way comparison allocated to None were also reported in the two-way None-versus-Short comparison, those allocated to Long were also reported in the two-way Short-versus-Long comparison, and those allocated to Short were also reported in both the two-way None-versus-Short comparison and the two-way Short-versus-Long comparison. The findings of both two-way comparisons from RADICALS-HD are described in detail elsewhere [4,5], as are the results from the protocol’s RT timing randomisation, RADICALS-RT [6,7]. The protocol is available online (https://www.mrcctu.ucl.ac.uk/media/1811/radicals-protocol-version-60-14-dec-2018_signed.pdf).

Randomisation used the method of minimisation with a random element, stratified by Gleason score, positive margins, RT timing, planned RT schedule, and planned ADT type. ADT was to be initiated as soon as possible after randomisation. Follow-up was scheduled for every 4 mo for the first 2 yr, then 6-monthly to 5 yr, and annually thereafter.

ADT, if allocated, was given with local choice of a gonadotrophin-releasing hormone analogue (GnRHa), initially supplemented by 3 wk of an antiandrogen started 1 wk prior to the first GnRHa administration. A choice to use monthly injections was encouraged in the Short group. Outside Canadian sites, bicalutamide monotherapy 150 mg daily or degarelix was an acceptable alternative.

RT was commenced approximately 2 mo after starting ADT, if allocated. The choice of an intended RT schedule was prespecified for each participant as either 52.5 Gy in 20 fractions over 4 wk or 66.0 Gy in 33 fractions over 6.5 wk. RT was to include the prostate bed and could also include pelvic lymph nodes.

The primary outcome measure was metastasis-free survival (MFS), defined as any distant metastasis or death from any cause. The secondary outcome measures included freedom from distant metastasis (any distant metastasis or death from prostate cancer), overall survival (death from any cause), and initiation of nonprotocol ADT.

The sample size for RADICALS-HD was determined, overall, by the two two-way comparisons, after most early trial participants elected one of the two-way randomisations. Therefore, although the three-way randomisation provided the only randomised data directly comparing the None and Long groups, the oversight committees each acknowledged from an early stage that this comparison would not be powered to conventional levels. The Independent Data Monitoring Committee met to review data from RADICALS on ten occasions and did not recommend data to be released early. The timing of this analysis has been triggered by the reporting of the two two-way comparisons reaching their target number of events for analysis.

The full statistical analysis plan is published elsewhere [3] and is summarised here. For this three-way randomisation, primary effect estimates compared against no treatment, that is, Long with None and Short with None, and the comparison of Long with Short is also reported. Median follow-up was calculated by reverse censoring on death. All analyses followed the principle of intention to treat. The statistical significance of any difference overall between the three randomised groups was evaluated with a logrank test, stratified by randomisation stratification factors. The prioritised approach for summarising the estimated treatment effect followed checking the proportional hazards assumption with the Grambsch-Therneau test: if there was no evidence (*p* ≥ 0.10) of nonproportional hazards, the effect estimate as a hazard ratio (HR) from Cox regression models (stratified for the randomisation factors) was prioritised; if there was evidence of nonproportional hazards-which makes HRs difficult to interpret–restricted mean “survival” time (RMST) was used as the primary estimate of effect, estimated from a flexible polynomial model, accounting for the randomisation stratification factors and with time restricted (t*) to 10 yr after randomisation. All analyses were done in Stata version 17 (StataCorp, College Station, TX, USA). For each outcome measure, from a three-arm model, both approaches are presented for completeness, and RMST is a useful summary measure regardless of proportionality. RMST differences, where reported, were calculated in pairwise models using the strmst command. Time -to event graphs were presented using the extended risk table of the KMunicate format, but without additional confidence intervals (CIs) to simplify the viewing [8]. CIs are constructed with a 95% confidence level. Events rates at specified times were taken from Kaplan-Meier survival functions.

## 3 Results

**Fig. 1:**
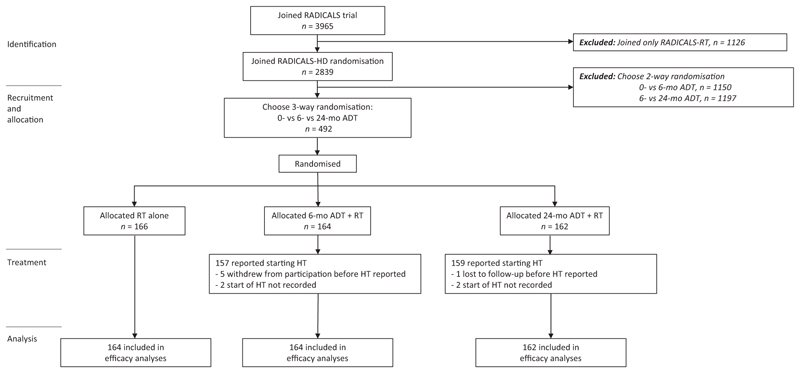
CONSORT diagram. ADT = androgen deprivation therapy; HT = hormone therapy; RT = radiotherapy.

Between 22 November 2007 and 29 June 2015, 492 participants planned for postoperative RT were allocated randomly in RADICALS-HD’s three-way comparison across the three treatment arms: no ADT (None, *n* = 166), short-course 6-mo ADT (Short, *n* = 164), or long-course 24-mo ADT (Long, *n* = 162; Fig. 1).

**Fig. 2:**
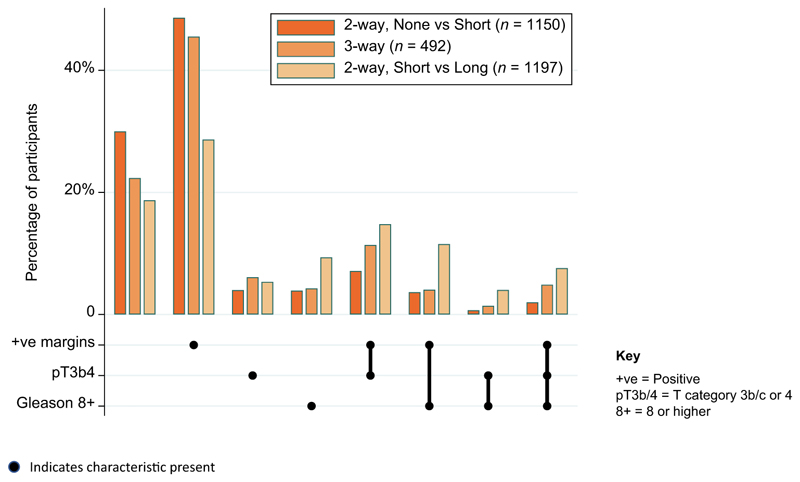
UpSet plot of participant characteristics, by RADICALS-HD randomisation. ADT = androgen deprivation therapy; Long = addition of 24 mo of ADT; None = no addition of ADT; Short = addition of 6 mo of ADT.

Most participants were from the UK (92%), with others from Canada (4%) and Denmark (4%). The median age of participants at randomisation was 66 yr (interquartile range [IQR] 61, 69). At surgery, 64 (13%) had Gleason score <7, 229 (47%) had Gleason score 3 + 4, 127 (26%) had Gleason score 4 + 3, 72 (15%) had Gleason score 8 plus, and 117/489 (24%) had stage ≥T3b disease (Table 1). Figure 2 shows, as an UpSet plot, the distribution of disease characteristics for participants in the three-way randomisation overall, contextualised by the participants in each of the previously reported two-way randomisations. The characteristics sit between the two-way comparisons, with participants most commonly having only positive margins as a randomisation risk factor with no risk factors ranking as second most common.

Postoperative RT was initiated in 300/492 (61%) participants in the salvage setting and in 192/492 (39%) in the adjuvant setting. The most common, planned RT schedule was 66 Gy in 33 fractions for 347/492 (71%), and the RT target was most commonly prostate bed only for 459/492 (93%). The median reported time to starting ADT was similar in the Short and Long groups: 7 d (IQR 1, 14) and 8 d (IQR 2, 15) respectively. The median time to the last reported administration of ADT was 5 mo (Short: IQR 3, 6) and 22 mo (Long: IQR 19, 24).

Follow-up at sites of trial participants ended on 31 December 2021: 392 participants were still in followup at that time; 66 were known to have died, and 34 had previously chosen to stop their participation or had been lost to follow-up. The median follow-up was 9.0 yr (IQR 7.1, 11.0). Among those still in active follow-up at the end of the trial, the minimum follow-up was 5.9 yr. The database was locked on May 27, 2022.

**Fig. 3:**
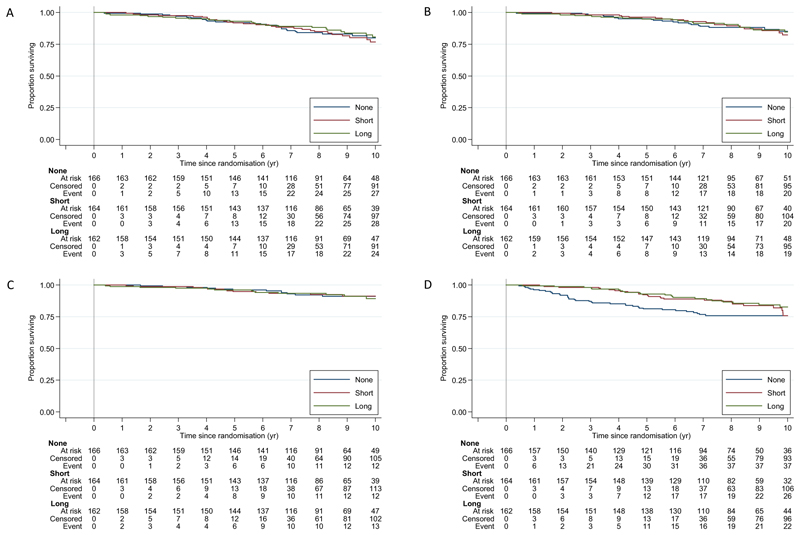
Primary and secondary outcome measures (A) metastasis-free survival, (B) overall survival, (C) freedom from distant metastasis, and (D) time to salvage hormone therapy. ADT = androgen deprivation therapy; Long = addition of 24 mo of ADT; None = no addition of ADT; Short = addition of 6 mo of ADT.

MFS events were reported for 89 participants (32 None, 31 Short, and 26 Long). There was no evidence of a difference between the three treatment groups overall (logrank *p* = 0.98). Given no evidence of nonproportional hazards, overall, in the treatment effect (*p* = 0.43), the estimated treatment effect on MFS is summarised for Long versus None as HR = 0.95 (95% CI 0.54–1.68), for Short versus None as HR = 1.01 (95% CI 0.57–1.76), and for Long versus Short as HR = 0.81 (95% CI 0.45–1.44; Table 2 and Fig. 3). After 10 yr, 80.1% in the None, 76.8% in the Short, and 80.7% in the Long group were alive without metastatic disease.

Only 66 deaths had been reported in total, and these were mostly attributed to other causes. Of 22, 23, and 21 deaths reported for None, Short, and Long, five, three, and six deaths were attributed to prostate cancer, respectively.

A statistically significant difference was observed between the three randomised groups in the time from randomisation to initiation of salvage ADT (overall logrank *p* = 0.026; Table 2). Given the evidence of nonproportional hazards, this is primarily summarised as differences in RMST, restricted to 10 yr, where there was evidence of longer time to initiation of salvage ADT for Short versus None (RMST difference = 0.81 yr; 95% CI 0.24, 1.38) and Long versus None (RMST difference = 0.90 yr; 95% CI 0.36, 1.44). For the other secondary outcome measures, there was no statistically significant difference between the three randomised groups in overall survival (overall logrank *p* = 0.940) or in freedom from distant metastases (overall logrank *p* = 0.768; Table 2).

## 4 Discussion

There was no evidence of a benefit from the addition of 2 yr of ADT to postoperative RT for patients with previous radical prostatectomy for whom both no ADT and short-term ADT were considered. These are primary data from a randomised comparison, but this comparison was under-powered and the results cannot exclude the possibility of a relative benefit in MFS up to 47% or a relative detriment up to 68%. The data from this comparison will be included in the DADSPORT meta-analysis that will provide the best estimate of the benefit from adding hormone therapy to postoperative RT [9].

An improvement in long-term clinical outcomes with the addition of 2 yr of hormone therapy to postoperative RT has been reported elsewhere. The RTOG 9601 trial, which tested androgen blockade using bicalutamide monotherapy rather than testosterone suppression using GnRH therapy, reported an improvement in overall survival (HR = 0.77) as well as MFS [10]. The RADICALS-HD two-way comparison of Short versus Long showed an improvement in MFS for 2-yr ADT over 6-mo ADT (HR = 0.77; 95% CI 0.61–0.97; *p* = 0.029) [2,5]. That comparison comprised mostly people for whom ADT was considered, by their managing clinicians, to be definitely indicated: 1197/1523 were randomised only between Short and Long, plus 326 were randomised between None, Short, and Long (reported here).

Seeing no clear evidence of improvement in MFS in this direct comparison of 2 yr of ADT with no ADT in RADICALS-HD, should one exist, is not surprising because of the small sample size, patient characteristics, and subsequent low event rate. The broad CIs around the point estimate of the effect size are consistent with the results of the previous trials. Postoperative RT was given at lower prostate-specific antigen (PSA) levels in RADICALS-HD than in RTOG 9601. This is pertinent because a subgroup analysis of RTOG 9601 suggested that the benefit of 2 yr of hormone therapy was greater in participants with a pre-RT PSA value of >0.6 ng/ml [11]; only 14% (46/328) of patients in the None-versus-Long comparison had a pre-RT PSA value of >0.6 ng/ml.

**Fig. 4:**
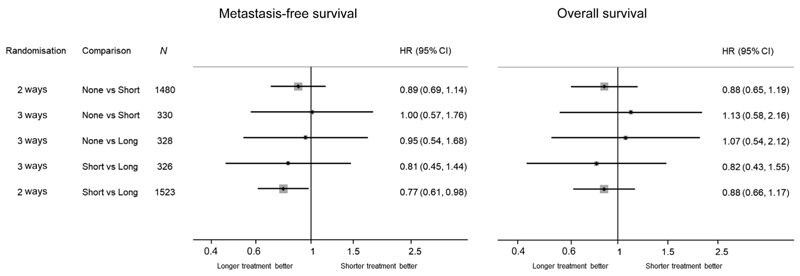
Summary of two- and three-way RADICALS-HD randomisations. ADT = androgen deprivation therapy; CI = confidence interval; HR = hazard ratio; Long = addition of 24 mo of ADT; None = no addition of ADT; Short = addition of 6 mo of ADT.

Given the previous evidence that long-term ADT improves the outcome of postoperative RT, some might be surprised that the HR for MFS in the current analysis of None versus Long appears so close to no effect. It may be instructive to visualise the estimates of the treatment effect on MFS for each pairwise comparison in RADICALS-HD from both the three-way randomisation and the two two-way randomisations (published elsewhere; Table 3 and Fig. 4). The CIs for each comparison in the three-way randomisation are, of course, much wider. These findings from None versus Long are not inconsistent with the reports from RTOG 9601.

When RADICALS-HD was first designed, it was intended to be a single trial with only the three-way randomisation (None vs Short vs Long). However, partly to simplify discussions around co-participation in RADICALS-RT where suitable, patients and investigators had the option of entering either of the two two-way randomisations (None vs Short or Short vs Long) in the early parts of the trial. Each two-way randomisation (*n* = 2347 in total) recruited much better than the three-way randomisation (*n* = 492). This reflected the common view for each potential participant that either ADT was definitely needed or long-term ADT should be avoided. If all participants had entered the three-way randomisation, as was encouraged by the Trial Management Group, the trial should have also been powered for the None-versus-Long comparison, and overall recruitment would have been reduced by around 25% and the time taken to read out shortened by several years. If the trial were being designed now, consideration may have been given to implementing the newer ROCI design [12], which would have allowed duration ranging and modelling to assess the most appropriate duration of ADT or a PRACTICAL design [13], given that each duration was in common practice already with no agreed standard of care.

The deliberate simplicity of the trial was also reflected in the choice not to collect data on adverse effects from hormone therapy; this was felt to be sufficiently well known, in terms of both clinician-reported and patient-reported scales. Therefore, there are no adverse event data or patient-reported outcome data to report here, with the latter being collected only in RADICALS-RT [7]. Similarly, testosterone recovery times were not recorded. Prostate-specific membrane antigen positron emission tomography scans, which are now used increasingly, were not available at the start of the trial, so the trial did not record the type of imaging modality. The large majority of metastatic events reported here will have been detected on bone scan, magnetic resonance imaging, or computed tomography scan.

Current clinical guidelines are permissive regarding the use of ADT with postoperative RT. For example, the ESMO clinical practice guidelines for prostate cancer state that “ADT .... may be offered to men having salvage RT” [14]. Such guidelines should now be reviewed in the light of all the data from RADICALS-HD. Overall, our view is that the results from the None-versus-Short comparison [4] strengthen the case for salvage RT alone, without ADT, at least in men with relatively favourable disease characteristics (lower pre-RT PSA, longer time since radical prostatectomy, and lower Gleason score). Conversely, if ADT is to be used with postoperative RT, particularly in men with more adverse disease characteristics, then the results of the Short-versus-Long comparison [5] strengthen the case for using 2 yr rather than 6 mo of treatment. The RADICALS-HD results overall raise doubts about the rationale for using 6 mo of ADT with postoperative RT. More evidence to inform the use of ADT with postoperative RT will be provided by the DADSPORT meta-analysis, which might help define the appropriate duration of ADT [9]. Genomic classifiers have been shown to predict the outcome of salvage RT and might also predict the benefit from the addition of ADT [15].

## 5 Conclusions

RADICALS-HD has demonstrated that men having postoperative RT after radical prostatectomy usually have a favourable outcome and that relatively few develop metastatic disease within 10 yr. For future trials to report in a reasonable timescale, it will be necessary to identify patients at a higher risk of metastases and prostate cancer death.

## Supplementary Material

Supplementary 1

## Figures and Tables

**Table 1:** Participant characteristics and prerandomisation planned treatment^a^

	None (*n* = 166)	Short (*n* = 164)	Long (*n* = 162)
**Age at rand^n^ (yr)**	65 (61–69)	66 (61–69)	67 (62–69)
**PSA at rand^n^ (ng/ml)**	0.23 (0.15–0.40)	0.20 (0.10–0.40)	0.21 (0.10–0.40)
**Gleason score**			
<7	21 (13)	24 (15)	19 (12)
3 + 4	75 (45)	72 (44)	82 (51)
4 + 3	43 (28)	44 (27)	40 (25)
8 plus	27 (16)	24 (15)	21 (13)
Missing	0 (NA)	0 (NA)	0 (NA)
**T stage**			
1/2	51 (31)	50 (31)	61 (38)
3a	79 (48)	68 (42)	63 (39)
3b/c	36 (22)	43 (26)	33 (21)
4	0 (0)	2 (1)	3 (2)
Missing	0	1 (NA)	2 (NA)
**Lymph node involvement**			
Node negative	87 (53)	87 (53)	86 (53)
Node positive	7(4)	8(3)	7(4)
No dissection	71 (43)	69 (43)	69 (43)
Missing	1 (NA)	0 (NA)	0 (NA)
**Positive margins**			
Absent	57 (34)	55 (32)	56 (35)
Present	109 (66)	109 (66)	106 (65)
Missing	0 (NA)	0 (NA)	0 (NA)
**CAPRA-S score**			
Low (0–2)	16 (10)	15 (9)	20 (12)
Intermediate (3–5)	78 (48)	81 (49)	87 (54)
High (6+)	69 (42)	68 (41)	55 (34)
Missing	3 (NA)	0 (NA)	0 (NA)
**Country**			
UK	154 (93)	147 (90)	152 (94)
Canada	5 (3)	10 (6)	3 (2)
Denmark	6 (4)	6 (4)	6 (4)
Ireland	1 (1)	1 (1)	1 (1)
**Timing of radiotherapy**			
Adjuvant	64 (39)	66 (40)	62 (38)
Early salvage	102 (61)	98 (60)	100 (62)
**Planned RT schedule**			
52.5 Gy/20 f	41 (25)	40 (24)	43 (27)
66.0 Gy/33 f	118 (71)	117 (74)	112 (69)
Other	7 (4)	7 (4)	7 (4)
Missing	0 (NA)	0 (NA)	0 (NA)
**Planned RT target**			
Prostate bed	153 (92)	153 (93)	153 (94)
Prostate bed + lymph nodes	13 (8)	11 (7)	9 (6)
Missing	0 (NA)	0 (NA)	0 (NA)
**Planned ADT (if allocated)**			
GnRH agonist	144 (87)	143 (87)	138 (86)
Bicalutamide	22 (13)	21 (13)	23 (14)
Missing	0 (NA)	0 (NA)	1 (NA)

ADT = androgen deprivation therapy; f = fractions; GnRH = gonadotrophin-releasing hormone; NA = not available; PSA = prostate-specific antigen; Rand^n^ = randomisation; RT = radiotherapy.

a Data are presented as *n* (%) or median (quartiles).

**Table 2:** Primary and secondary outcome measures

	Logrank *p* value	None (*n* = 166)		Short (*n* = 164)		Long (*n* = 162)	
**Metastasis-free survival** ^a^							
Events		32		31		26	
Metastases first		15		12		12	
Prostate cancer death first		1		1		1	
Death from other causes first		16		18		13	
10-yr event free for MFS (%)		81		77		81	
RMST (yr) ^b^		9.18	(8.89, 9.47)	9.14	(8.84, 9.44)	9.22	(8.91, 9.53)
Overall logrank *p* value ^c^	0.98						
Hazard ratio (95% CI) ^d^ vs none		NA		1.01	(0.57, 1.76)	0.95	(0.54, 1.68)
**Overall survival** ^e^							
Events		22		23		21	
10-yr survival (%)		85		82		85	
RMST (yr) ^b^		9.43	(9.18, 9.68)	9.39	(9.14, 9.64)	9.44	(9.18, 9.69)
Overall logrank p value ^c^	0.94						
Hazard ratio (95% CI) ^d^ vs none		NA		1.12	(0.59, 2.16)	1.07	(0.54, 2.12)
**Freedom from distant metastasis** ^f^							
Events		16		13		13	
10-yr event free for FFDM (%)		91		91		89	
RMST (yr) ^b^		9.58	(9.36, 9.80)	9.60	(9.39, 9.82)	9.54	(9.28, 9.80)
Overall logrank *p* value ^c^	0.77						
Hazard ratio (95% CI) ^d^ vs none		NA		0.79	(0.33, 1.89)	1.09	(0.50, 2.37)
**Time to salvage hormone therapy** ^g^							
Events		38		26		22	
10-yr event free (%)		76		76		83	
RMST (yr) ^b^		8.42	(7.95, 8.88)	9.11	(8.78, 9.43)	9.35	(9.08, 9.63)
Overall logrank p value ^c^	0.026						
RMST difference (yr) vs none ^h^		NA		0.81	(0.24, 1.38)	0.90	(0.36, 1.44)

ADT = androgen deprivation therapy; CI = confidence interval; FFDM = freedom from distant metastasis; HR = hazard ratio; MFS = metastasis-free survival; NA = not available; RMST = restricted mean survival time; RT = radiotherapy.

a No evidence of nonproportionality in the treatment effect: Grambsch-Therneau test of nonproportional hazards *p* = 0.43—primarily summarise effect with hazard ratio.

b RMST = restricted mean “survival” time (95% CI).

c Test of difference between all three groups, adjusted for randomisation stratification factors.

d Estimate of treatment effect: hazard ratio relative to RT alone, adjusted for randomisation stratification factors.

e No evidence of nonproportionality in the treatment effect: Grambsch-Therneau test of nonproportional hazards *p* = 0.70—primarily summarise effect with hazard ratio.

f No evidence of nonproportionality in the treatment effect: Grambsch-Therneau test of nonproportional hazards *p* = 0.10—primarily summarise effect with hazard ratio.

g No evidence of nonproportionality in the treatment effect: Grambsch-Therneau test of nonproportional hazards *p* = 0.033—primarily summarise effect with RMST. For short-term versus no ADT, HR = 0.48 (95% CI 0.26, 0.90), and for long-term versus no ADT, HR = 0.54 (95% CI 0.31, 0.95).

h RMST difference was taken from pairwise models.

**Table 3:** Summary of two- and three-way RADICALS-HD comparisons, MFS, and overall survival

Comparison	Context	*N*	Reference	Comparator	HR (95% CI)	Cross-Ref
**Metastasis-free survival**						
None vs Short	2-way randomisation	1480	None	Short	0.89 (0.69, 1.14)	Published elsewhere [4]
None vs Short	Subset in three-way randomisation	330	None	Short	1.01 (0.57, 1.76)	Here
None vs Long	3-way randomisation	328	None	Long	0.95 (0.54, 1.68)	Here
Short vs Long	Subset in three-way randomisation	326	Short	Long	0.81 (0.45, 1.44)	Here
Short vs Long	2-way randomisation	1523	Short	Long	0.77 (0.61, 0.98)	Published elsewhere [5]
**Overall survival**						
None vs Short	2-way randomisation	1480	None	Short	0.88 (0.65, 1.19)	Published elsewhere [4]
None vs Short	Subset in three-way randomisation	330	None	Short	1.12 (0.59, 2.16)	Here
None vs Long	3-way randomisation	328	None	Long	1.07 (0.54, 2.12)	Here
Short vs Long	Subset in three-way randomisation	326	Short	Long	0.82 (0.43, 1.55)	Here
Short vs Long	2-way randomisation	1523	Short	Long	0.88 (0.66, 1.17)	Published elsewhere [5]

ADT = androgen deprivation therapy; CI = confidence interval; HR = hazard ratio; Long = addition of 24 mo of ADT; MFS = metastasis-free survival; *N* = number of participants; None = no addition of ADT; Short = addition of 6 mo of ADT.

## Data Availability

The datasets are available upon request as per the moderated access approach of the MRC CTU at UCL. Please contact the corresponding author for more information or visit https://www.mrc-ctu.ucl.ac.uk/our-research/other-research-policy/data-sharing/.

## Advancing Practice

### What does this study add?

These results will inform clinical decision-making on the duration of androgen deprivation therapy (ADT) to use with postoperative radiotherapy for prostate cancer. The findings are contextualised with respect to the trial’s other results and the participant characteristics. Future research should focus on participants at a higher risk of developing metastatic disease after radical prostatectomy.

### Clinical Relevance

This three-way randomized trial provides the highest level evidence to date comparing metastasis-free survival (MFS) outcomes following postoperative radiotherapy after prostatectomy among patients receiving 0, 6, or 24 months of androgen deprivation therapy (ADT). With 492 patients enrolled, and a median follow-up of 9 years, there was no evidence of any difference in MFS between the groups. Two important caveats exist. First, this trial was for patients who were considered suitable to be randomized to receive 0 ADT; i.e., this represents a more favorable group overall. Second, this trial was not powered to detect small differences in MFS. Future trials should focus on patient groups most likely to harbour an increased risk of metastasis. Associate Editor: Amar U Kishan

### Patient Summary

A total of 492 participants due for radiotherapy (RT) after previous prostate cancer surgery were allocated to RT alone, RT with 6 mo of hormone therapy, or RT with 24 mo of hormone therapy. No meaningful differences were clearly seen in long-term outcomes between the three groups.
