# Supplementary material for: Randomised Trial of No, Short-term, or Long-term Androgen Deprivation Therapy with Postoperative Radiotherapy After Radical Prostatectomy: Results from the Three-way Comparison of RADICALS-HD (NCT00541047)
Source: Eur Urol. Author manuscript; Available in PMC 2025 Jan 8. (PMC7617288; doi:10.1016/j.eururo.2024.07.026)
Supplement: Supplementary 1 [file EMS201284-supplement-Supplementary_1.zip › 1-s2.0-S0302283824025156-mmc2.docx]

**Supplementary Table 1: Primary and Secondary Outcome Measures for RT+LTHT vs RT+STHT**

|  | **Logrank** | **Short** | | **Long** | |
| --- | --- | --- | --- | --- | --- |
|  | **P** | **(n=164)** | | **(n=162)** | |
| **Metastasis-free survival** |  |  |  |  |  |
| Events |  | 31 |  | 26 |  |
| Logrank p-value* | 0.47 |  |  |  |  |
| Hazard ratio (95%CI)† ** |  | N/A |  | 0.81 | (0.45, 1.44) |
|  |  |  |  |  |  |
| **Overall survival** |  |  |  |  |  |
| Events |  | 23 |  | 21 |  |
| Logrank p-value * | 0.54 |  |  |  | |
| Hazard ratio (95%CI)† ** |  | N/A |  | 0.82 | (0.43, 1.55) |
|  |  |  |  |  |  |
| **Freedom-from-distant-metastasis** |  |  |  |  |  |
| Events |  | 13 |  | 13 |  |
| Logrank p-value * | 0.68 |  |  |  |  |
| Hazard ratio (95%CI)† ** |  | N/A |  | 1.21 | (0.50, 2.93) |
|  |  |  |  |  |  |
| **Time to salvage hormone therapy** |  |  |  |  |  |
| Events |  | 26 |  | 22 |  |
| Logrank p-value * | 0.77 |  |  |  | |
| Hazard ratio (95%CI)^†^ ** |  | N/A |  | 1.10 | (0.57, 2.13) |
|  |  |  |  |  |  |

* Test of difference between two groups, adjusted for randomisation stratification factors

** Hazard ratio relative for Long relative to Short

† There was no evidence of non-proportional hazards in the estimates of the treatment effects with p-values from a Grambsch-Therneau test of 0.546, 0.393, 0.647 and 0.791, respectively.

**Supplementary Table 2: Exploratory subgroup analysis of randomisation stratification factors on metastases-free survival (MFS)**

|  | **None** | | | **Short** | | | **Long** | | |  | **Treatment effect** | | | |  |
| --- | --- | --- | --- | --- | --- | --- | --- | --- | --- | --- | --- | --- | --- | --- | --- |
|  | **(n=166)** | | | **(n=164)** | | | **(n=162)** | | | **Short vs None** | | | **Long vs None** | | **Interaction^Ψ^** |
|  | N | Events | (%) | N | Events | (%) | N | Events | (%) | HR | (95%CI) | HR | | (95% CI) | **P** |
| **Gleason score** |  |  |  |  |  |  |  |  |  |  |  |  | |  |  |
| <7 | 21 | 3 | (14%) | 24 | 3 | (13%) | 19 | 6 | (32%) | 0.80 | (0.15, 4.13) | 2.79 | | (0.70, 11.2) | 0.178 |
| 7 | 118 | 21 | (18%) | 116 | 22 | (19%) | 122 | 17 | (14%) | 1.07 | (0.59, 1.95) | 0.73 | | (0.39, 1.39) |  |
| 8+ | 27 | 8 | (30%) | 24 | 6 | (25%) | 21 | 3 | (14%) | 0.69 | (0.23, 2.02) | 0.43 | | (0.12, 1.65) |  |
| **Positive margins** |  |  |  |  |  |  |  |  |  |  |  |  | |  |  |
| Absent | 57 | 8 | (14%) | 55 | 14 | (25%) | 56 | 8 | (14%) | 1.91 | (0.80, 4.56) | 0.90 | | (0.34, 2.41) | 0.11 |
| Present | 109 | 24 | (22%) | 109 | 17 | (16%) | 106 | 18 | (17%) | 0.67 | (0.36, 1.25) | 0.76 | | (0.41, 1.40) |  |
| **Planned RT schedule** |  |  |  |  |  |  |  |  |  |  |  |  | |  |  |
| 52.5Gy/20f | 41 | 12 | (29%) | 40 | 12 | (30%) | 43 | 7 | (16%) | 0.96 | (0.43, 2.13) | 0.49 | | (0.19, 1.24) | 0.24 |
| 66Gy/33f | 118 | 18 | (15%) | 117 | 18 | (15%) | 112 | 19 | (17%) | 1.01 | (0.53, 1.94) | 1.13 | | (0.59, 2.15) |  |
| Other^θ^ | 7 | 2 | (29%) | 7 | 1 | (14%) | 7 | 0 | (0%) | 0.42 | (0.04, 4.69) | N/D | |  |  |
| **Timing of RT** |  |  |  |  |  |  |  |  |  |  |  |  | |  |  |
| Adjuvant | 64 | 12 | (19%) | 66 | 15 | (23%) | 62 | 10 | (16%) | 1.32 | (0.62, 2.81) | 0.78 | | (0.34, 1.82) | 0.49 |
| Salvage | 102 | 20 | (20%) | 98 | 16 | (16%) | 100 | 16 | (16%) | 0.75 | (0.39, 1.46) | 0.80 | | (0.42, 1.55) |  |
| **Planned ADT** |  |  |  |  |  |  |  |  |  |  |  |  | |  |  |
| LHRH agonist | 144 | 26 | (18%) | 143 | 29 | (20%) | 138 | 20 | (14%) | 1.16 | (0.68, 1.97) | 0.81 | | (0.45, 1.45) | 0.11 |
| Bicalutamide | 22 | 6 | (27%) | 21 | 2 | (10%) | 23 | 6 | (26%) | 0.26 | (0.05, 1.32) | 0.65 | | (0.21, 2.07) |  |

N/D=Not done

^Ψ^ Chi-squared test for interaction in Cox regression model

^θ^ Excluded from assessment in interaction test
