# Supplementary material for: Randomised Trial of No, Short-term, or Long-term Androgen Deprivation Therapy with Postoperative Radiotherapy After Radical Prostatectomy: Results from the Three-way Comparison of RADICALS-HD (NCT00541047)
Source: Eur Urol. Author manuscript; Available in PMC 2025 Jan 8. (PMC7617288; doi:10.1016/j.eururo.2024.07.026)
Supplement: Supplementary 1 [file EMS201284-supplement-Supplementary_1.zip › 1-s2.0-S0302283824025156-mmc3.pdf]

RADICALS OVERSIGHT COMMITTEES, STAFF AND COLLABORATORS

Version: 11-Jul-2023

CONTENTS

NOTES 2
TRIAL MANAGEMENT GROUP 3
INDEPENDENT DATA MONITORING COMMITTEE 4
TRIAL STEERING COMMITTEE 5
TRIALS UNIT STAFF 6
INVESTIGATORS AND COLLABORATORS: SITE STAFF 8
PARTICIPANTS 88

NOTES

Investigators and site staff are those who have formally appeared at any time on a site’s Delegation Logs.

CTU staff are those who have worke8d on or contributed to the trial any time from the outset until the date of this report.

The independent members of the Independent Data Monitoring Committee and Trial Oversight Committee play an important role in the conduct of the trial.

Industry collaborators are a subset of the people who have worked, on relevant sections, with the trial staff to ensure the trial runs efficiently.

TRIAL MANAGEMENT GROUP
(Listing only member external to the trials unit or senior staff at the trials unit)

Area	Status	Member	Geography	Role
Clinical/Surgical	Current	Charles Catton	Toronto, Canada	Country Lead
~~	~~~	Noel Clarke	Manchester, UK	
~~	~~~	William Cross	York, UK	Surgical Champion
~~	~~~	Howard Kynaston	Cardiff, UK	
~~	~~~	John Logue	Stockport, UK	RT advisor
~~	~~~	Peter Meidahl	Copenhagen, Denmark	Country Lead
~~	~~~	Chris Parker	Sutton, UK	Chief Investigator
~~	~~~	Heather Payne	London, UK	RT advisor
~~	~~~	Rajendra Persad	Bristol, UK	Surgical Champion
~~	~~~	Fred Saad	Montreal, Canada	
~~	Previous	Kilian Mellon	Leicester, UK	
~~	~~~	Chris Morash	Canada, UK	
~~	~~~	Garrett Durkan	Galway, Ireland	Country Lead
Nurse specialist	Current	Lorna Bower		
~~	Previous	Jane Gosling		
~~	~~~	Angela Lee		
PPI	Current	Peter Neville		
~~	~~~	Alan Stirling		
~~	Previous	Peter Barton		
~~	~~~	Ian Jamieson		
~~	~~~	Mike Sawkins		
~~	~~~	Jim Stansfeld		
Senior trials units	Current	Adrian Cook	MRC CTU at UCL	
~~	~~~	Max Parmar	MRC CTU at UCL	
~~	~~~	Wendy Parulekar	CCTG	
~~	~~~	Cheryl Pugh	MRC CTU at UCL	
~~	~~~	Matthew Sydes	MRC CTU at UCL	
~~	Previous	Claire Amos	MRC CTU at UCL	
~~	~~~	Silvia Forcat	MRC CTU at UCL	
~~	~~~	Barbara Uscinska	MRC CTU at UCL	

Key: PPI = Patient and public involvement
RT = Radiotherapy

Note: The full list of trials units staff is detailed below in a subsequent section.

INDEPENDENT DATA MONITORING COMMITTEE
(All members independent)

Member	Status	Role
Murray Brunt	Current	Chair 2
Mary Gospodorawicz	Current	Member
Jim Montie	~~~	~~~
Cindy Billingham	~~~	~~~
David Landau	Previous	Chair 1

TRIAL STEERING COMMITTEE
(Listing only independent members)

Member	Status	Role	
John Chester	Current	Chair 4	MRC CTU Cancer umbrella TSC
Emma Crosbie	~~~	~~~	MRC CTU Cancer umbrella TSC
Lucy Kilburn	~~~	~~~	MRC CTU Cancer umbrella TSC
Richard Stephens	~~~	~~~	MRC CTU Cancer umbrella TSC
Jeremy Whelan	Previous	Chair 2	MRC CTU Cancer umbrella TSC
Anne Thomas	~~~	Chair 3	MRC CTU Cancer umbrella TSC
Anne Russell	~~~	Member	MRC CTU Cancer umbrella TSC
Judith Bliss	~~~	~~~	MRC CTU Cancer umbrella TSC
Hisham Mehanna	~~~	~~~	MRC CTU Cancer umbrella TSC
David Guthrie	Previous	Chair 1	MRC CTU Cancer Urology TSC
Stan Dische	~~~	Member	MRC CTU Cancer Urology TSC
Michael Jewett	~~~	~~~	MRC CTU Cancer Urology TSC
John Scholefield	~~~	~~~	MRC CTU Cancer Urology TSC

TRIALS UNIT STAFF

Country	Trials Unit	Role	Status	Name
UK	MRC CTU at UCL	Statistician	Current	Adrian Cook
~~	~~~	~~~	Previous	Chris Brawley
~~	~~~	~~~	~~~	Andrew Embleton
~~	~~~	~~~	~~~	Gordana Jovic
~~	~~~	~~~	~~~	Rachel Morgan
~~	~~~	~~~	~~~	Max Parmar
~~	~~~	~~~	~~~	Matthew Sydes
~~	~~~	Clinical Project Manager	Current	Cheryl Pugh
~~	~~~	~~~	Previous	Claire Amos
~~	~~~	~~~	~~~	Silvia Forcat
~~	~~~	~~~	~~~	Barbara Uscinska
~~	~~~	Trial Manager	Previous	Cindy Goldstein
~~	~~~	~~~	~~~	Claire Murphy
~~	~~~	~~~	~~~	Dipa Noor
~~	~~~	~~~	~~~	Holly Pickering
~~	~~~	~~~	~~~	Carol Roach
~~	~~~	~~~	~~~	Hannah Sims
~~	~~~	Data Manager	Current	Christos Maniatis
~~	~~~	~~~	Previous	Katherine Beaney
~~	~~~	~~~	~~~	Katharine Bellenger
~~	~~~	~~~	~~~	Jenna Grabey
~~	~~~	~~~	~~~	Anna Herasimtschuk
~~	~~~	~~~	~~~	Paul Patterson
~~	~~~	~~~	~~~	Helena Ribeiro
~~	~~~	~~~	~~~	Fatimah Seray-Wurie
~~	~~~	~~~	~~~	Ben Spittle
~~	~~~	~~~	~~~	Lilian Tsang
~~	~~~	DMS PM	Current	Christina Chung
~~	~~~	~~~	Previous	Nancy Tappenden
~~	~~~	Data Scientist	Current	Fatima Mohamed
~~	~~~	~~~	Previous	Dominic Hague
~~	~~~	~~~	~~~	Lindsey Masters
~~	~~~	Programmer	Current	Preetha Shaji
~~	~~~	~~~	Previous	Zaheer Islam
~~	~~~	~~~	~~~	Mary Rauchenberger
Canada	CCTG	Physician Coordinator	Current	Wendy Parulekar
~~	~~~	~~~	Previous	Ralph Meyer
~~	~~~	~~~	~~~	Harriet Richardson
~~	~~~	Study Coordinator	Current	Cathy Davidson
~~	~~~	~~~	Previous	Conor Dellar
~~	~~~	~~~	~~~	Kate Whelan
~~	~~~	Clinical Trials Associate	Current	Karen Richardson
~~	~~~	~~~	Previous	Sue Casey

TRIALS UNIT STAFF

Country	Trials Unit	Role	Status	Name
~~	~~~	Research Associate	Previous	Mandy Fletcher
~~	~~~	~~~	~~~	Karen Murphy
~~	~~~	Oracle	Previous	Teddy Brown

INVESTIGATORS AND COLLABORATORS: SITE STAFF

Staff on site delegation logs

Country	Site	Names	Role
Canada	Abbotsford, BC: Fraser Valley Cancer Centre	Anand Karvat	Site PI
~~	~~~	Arthur Cheung	Clinical/Surgical
~~	~~~	Winkle Kwan	Clinical/Surgical
~~	~~~	Cheryl Carrasco	Principal CRA
~~	~~~	Gloria Garrioch	Principal CRA
~~	~~~	Janice Jong	Principal CRA
~~	~~~	Jen Darker	Principal CRA
~~	~~~	Cheryl Carrasco	Ethics CRA
~~	~~~	Gloria Garrioch	Ethics CRA
~~	~~~	Janice Jong	Ethics CRA
~~	~~~	Jen Darker	Ethics CRA
~~	~~~	Arlissa Johnson	Additional CRA
~~	~~~	Cathy Jackson	Additional CRA
~~	~~~	Cheryl Carrasco	Additional CRA
~~	~~~	Debbie Jepson	Additional CRA
~~	~~~	Donna Mitchell	Additional CRA
~~	~~~	Gloria Garrioch	Additional CRA
~~	~~~	Janice Jong	Additional CRA
~~	~~~	Michael W Braun	Additional CRA
~~	~~~	Monica Fourt	Additional CRA
~~	~~~	Noelle Baird	Additional CRA
~~	~~~	Rosanne Serpanchy	Additional CRA
~~	~~~	Sandeep Sandhu	Additional CRA
~~	~~~	Sue McIndoe	Additional CRA
~~	~~~	Helen Wu	Pharmacist
Canada	Barrie, ON: Royal Victoria Regional Health Centre	Christiaan Stevens	Site PI
~~	~~~	Adam Gladwish	Clinical/Surgical
~~	~~~	Frederick Yoon	Clinical/Surgical
~~	~~~	Gerard Morton	Clinical/Surgical
~~	~~~	Jason Yu	Clinical/Surgical
~~	~~~	Kimberley MarshGray	Principal CRA
~~	~~~	Michele Harris	Principal CRA
~~	~~~	Christine DiMarco	Ethics CRA
~~	~~~	Kayla Gerrity	Ethics CRA

INVESTIGATORS AND COLLABORATORS: SITE STAFF

Staff on site delegation logs

Country	Site	Names	Role
~~	~~~	Caitlin Pascoe	Additional CRA
~~	~~~	Cara Murphy	Additional CRA
~~	~~~	Ian Ding	Additional CRA
~~	~~~	Kayla Gerrity	Additional CRA
~~	~~~	Melanie Crawford	Additional CRA
~~	~~~	Michele Harris	Additional CRA
~~	~~~	Patricia MacIsaac	Additional CRA
~~	~~~	Rachelle Beausoleil	Additional CRA
~~	~~~	Sujata Pokhrel	Additional CRA
Canada	Brandon, MB: Western Manitoba Cancer Centre	Bashir Bashir	Site PI
~~	~~~	William Hunter	Site PI
~~	~~~	Arbind Dubey	Clinical/Surgical
~~	~~~	Bashir Bashir	Clinical/Surgical
~~	~~~	Gokulan Sivananthan	Clinical/Surgical
~~	~~~	William Hunter	Clinical/Surgical
~~	~~~	Joelle DuMontier	Principal CRA
~~	~~~	Leanne Anderson	Principal CRA
~~	~~~	Joelle DuMontier	Ethics CRA
~~	~~~	Leanne Anderson	Ethics CRA
~~	~~~	Leanne Anderson	Additional CRA
Canada	CAHN	Maroie Barkati	Site PI
~~	~~~	Carole Lambert	Clinical/Surgical
~~	~~~	Cynthia Menard	Clinical/Surgical
~~	~~~	Daniel Taussky	Clinical/Surgical
~~	~~~	Fred Saad	Clinical/Surgical
~~	~~~	Guila Delouya	Clinical/Surgical
~~	~~~	JeanPaul Bahary	Clinical/Surgical
~~	~~~	MarieClaude Beauchemin	Clinical/Surgical
~~	~~~	Pierre Rousseau	Clinical/Surgical
~~	~~~	Sophie Lavertu	Clinical/Surgical
~~	~~~	Adriana Carbonaro	Principal CRA
~~	~~~	Alexandra Frazzi	Principal CRA
~~	~~~	Chantal Lafleur	Principal CRA
~~	~~~	Siew Siew Pan	Principal CRA

INVESTIGATORS AND COLLABORATORS: SITE STAFF

Staff on site delegation logs

Country	Site	Names	Role
~~	~~~	Diane Trudel	Ethics CRA
~~	~~~	Mom Phat	Ethics CRA
~~	~~~	Chantal Lafleur	Additional CRA
~~	~~~	Mom Phat	Additional CRA
~~	~~~	Nidale ElSokhn	Additional CRA
~~	~~~	Silvine Benth	Additional CRA
Canada	Edmonton, AB: Cross Cancer Institute	Brita Danielson	Site PI
~~	~~~	Albert Murtha	Clinical/Surgical
~~	~~~	Don Yee	Clinical/Surgical
~~	~~~	Jim Rose	Clinical/Surgical
~~	~~~	John Oliver Amanie	Clinical/Surgical
~~	~~~	Matthew B Parliament	Clinical/Surgical
~~	~~~	Nadeem Pervez	Clinical/Surgical
~~	~~~	Nawaid Usmani	Clinical/Surgical
~~	~~~	Robert Pearcey	Clinical/Surgical
~~	~~~	Samir Patel	Clinical/Surgical
~~	~~~	Karen Tracey	Principal CRA
~~	~~~	Nirmal Joshi	Principal CRA
~~	~~~	Karen Tracey	Ethics CRA
~~	~~~	Nirmal Joshi	Ethics CRA
~~	~~~	Wanda Churchill	Ethics CRA
~~	~~~	Beverly Larson	Additional CRA
~~	~~~	Candra Williams	Additional CRA
~~	~~~	Carlie Smith	Additional CRA
~~	~~~	Debbie Mallett	Additional CRA
~~	~~~	Leni Santiago Garcia	Additional CRA
~~	~~~	Monika Lang	Additional CRA
~~	~~~	Nirmal Joshi	Additional CRA
~~	~~~	Wanda Churchill	Additional CRA
~~	~~~	Shelley Parker	Pharmacist
~~	~~~	Carol Borynec	Other
~~	~~~	Caroline Shewchuk	Other
~~	~~~	Colin Gramlich	Other
~~	~~~	Margaret Batz	Other

INVESTIGATORS AND COLLABORATORS: SITE STAFF
Staff on site delegation logs

Country	Site	Names	Role
~~	~~~	Sylvia McCrudden	Other
Canada	Halifax, NS: QEII Health Sciences Centre	David Bowes	Site PI
~~	~~~	Helmut Hollenhorst	Site PI
~~	~~~	Abdulla AlRashdan	Clinical/Surgical
~~	~~~	David Bowes	Clinical/Surgical
~~	~~~	Derek Wilke	Clinical/Surgical
~~	~~~	Nikhilesh Patil	Clinical/Surgical
~~	~~~	Paul K Joseph	Clinical/Surgical
~~	~~~	Robert DH Rutledge	Clinical/Surgical
~~	~~~	Erin Little	Principal CRA
~~	~~~	Heather Beaton	Principal CRA
~~	~~~	Kendra Dill	Principal CRA
~~	~~~	Emily Moffatt	Ethics CRA
~~	~~~	Jennifer MacVicar	Ethics CRA
~~	~~~	Joan Nieforth	Ethics CRA
~~	~~~	Robin Simpson	Ethics CRA
~~	~~~	Stevie Dugas	Ethics CRA
~~	~~~	Alison Avery	Additional CRA
~~	~~~	Angela MacDonald	Additional CRA
~~	~~~	Brittany Bond	Additional CRA
~~	~~~	Camryn Salyzyn	Additional CRA
~~	~~~	Connie Zinck	Additional CRA
~~	~~~	Donna Sutherland	Additional CRA
~~	~~~	Heather Walker	Additional CRA
~~	~~~	Jillian McCracken	Additional CRA
~~	~~~	Kara Bursey	Additional CRA
~~	~~~	Kathy MacIsaac	Additional CRA
~~	~~~	Kelsey van der Rijt	Additional CRA
~~	~~~	Lane Carvery	Additional CRA
~~	~~~	Lorrie Yunace	Additional CRA
~~	~~~	Lynn Hubley	Additional CRA
~~	~~~	Susan Burbridge	Additional CRA
~~	~~~	Tanya Yeo	Additional CRA
~~	~~~	Tina Rose	Additional CRA

INVESTIGATORS AND COLLABORATORS: SITE STAFF
Staff on site delegation logs

Country	Site	Names	Role
~~	~~~	Victoria Roberts	Additional CRA
~~	~~~	Claudia Harding	Pharmacist
~~	~~~	June Hodder	Pharmacy technician
~~	~~~	Kim BrucePayne	Other
Canada	Hamilton, ON: Juravinski Cancer Centre at Hamilton Health Sciences	Thomas B Corbett	Site PI
~~	~~~	Himu Lukka	Clinical/Surgical
~~	~~~	Ian S Dayes	Clinical/Surgical
~~	~~~	Jason W T Wong	Clinical/Surgical
~~	~~~	Malti Behn Patel	Clinical/Surgical
~~	~~~	Susan O Gudelis	Clinical/Surgical
~~	~~~	Thedoros Tsakirdis	Clinical/Surgical
~~	~~~	Diane DeRosa	Principal CRA
~~	~~~	Elaine Hill	Ethics CRA
~~	~~~	Yvonne Kinrade	Ethics CRA
~~	~~~	Anne Laughlin	Additional CRA
~~	~~~	Barbara Makepeace	Additional CRA
~~	~~~	Bianca Bier	Additional CRA
~~	~~~	Carmela Oliverio	Additional CRA
~~	~~~	Catherine Bucci	Additional CRA
~~	~~~	Diane DeRosa	Additional CRA
~~	~~~	Linda Nielsen	Additional CRA
~~	~~~	Lynn Marshall	Additional CRA
~~	~~~	Robin Eady	Additional CRA
Canada	Kelowna, BC: Cancer Centre for the Southern Interior	David W Petrik	Site PI
~~	~~~	Mohamed Manji	Site PI
~~	~~~	Bernard Lee	Clinical/Surgical
~~	~~~	David Kim	Clinical/Surgical
~~	~~~	David W Petrik	Clinical/Surgical
~~	~~~	Francois Bachand	Clinical/Surgical
~~	~~~	Juanita Crook	Clinical/Surgical
~~	~~~	Melanie Reed	Clinical/Surgical
~~	~~~	Mohamed Manji	Clinical/Surgical
~~	~~~	Ross Halperin	Clinical/Surgical
~~	~~~	Allana Scarfo	Principal CRA

INVESTIGATORS AND COLLABORATORS: SITE STAFF

Staff on site delegation logs

Country	Site	Names	Role
~~	~~~	Cherie Bates	Principal CRA
~~	~~~	Jeaneen Rudolph	Principal CRA
~~	~~~	Jessica Stojcic	Principal CRA
~~	~~~	Allana Scarfo	Ethics CRA
~~	~~~	Cherie Bates	Ethics CRA
~~	~~~	Jeaneen Rudolph	Ethics CRA
~~	~~~	Jessica Stojcic	Ethics CRA
~~	~~~	Aldyn Overend	Additional CRA
~~	~~~	Allana Scarfo	Additional CRA
~~	~~~	Brianna Creelman	Additional CRA
~~	~~~	Carol Winn	Additional CRA
~~	~~~	Cherie Bates	Additional CRA
~~	~~~	Danielle GeorgeChayka	Additional CRA
~~	~~~	Debbie Opitz	Additional CRA
~~	~~~	Flo Kronstal	Additional CRA
~~	~~~	Janet Yanchuk	Additional CRA
~~	~~~	Jeaneen Rudolph	Additional CRA
~~	~~~	Karen Wilkie	Additional CRA
~~	~~~	Kirsten Allen	Additional CRA
~~	~~~	Marie McClelland	Additional CRA
~~	~~~	Michaella Watson	Additional CRA
~~	~~~	Nancy Hartt	Additional CRA
~~	~~~	Shannon Cotts	Additional CRA
~~	~~~	Layton Carefoot	Pharmacist
~~	~~~	TuongVan Kim	Pharmacist
~~	~~~	Jill A Hayes	Other
~~	~~~	Tanya Lew	Other
Canada	Kingston, ON: Kingston Health Sciences Centre	Khaled O Zaza	Site PI
~~	~~~	Aamer Mahmud	Clinical/Surgical
~~	~~~	Carey Shenfield	Clinical/Surgical
~~	~~~	Maria Kalyvas	Clinical/Surgical
~~	~~~	Michael Brundage	Clinical/Surgical
~~	~~~	Vikaash Kumar	Clinical/Surgical
~~	~~~	Amber Burley	Principal CRA

INVESTIGATORS AND COLLABORATORS: SITE STAFF

Staff on site delegation logs

Country	Site	Names	Role
~~	~~~	Carrie Lindsay	Principal CRA
~~	~~~	Fred Zeltser	Principal CRA
~~	~~~	Jessica Ferguson	Principal CRA
~~	~~~	Amber Burley	Ethics CRA
~~	~~~	Angel Zhou	Ethics CRA
~~	~~~	Ashlee Young	Ethics CRA
~~	~~~	Carrie Lindsay	Ethics CRA
~~	~~~	Chloe Sutton	Ethics CRA
~~	~~~	Cindy Carlson	Ethics CRA
~~	~~~	Deborah Leach	Ethics CRA
~~	~~~	Denise KirbyBello	Ethics CRA
~~	~~~	Heather Doucette	Ethics CRA
~~	~~~	James Medd	Ethics CRA
~~	~~~	Jennifer Pritchard	Ethics CRA
~~	~~~	Jessica Ferguson	Ethics CRA
~~	~~~	Karen MacVicar	Ethics CRA
~~	~~~	Kristina Carmichael	Ethics CRA
~~	~~~	Noureen Hassan	Ethics CRA
~~	~~~	Rhonda Harpell	Ethics CRA
~~	~~~	Stephanie Raposo	Ethics CRA
~~	~~~	Tracy Vermette	Ethics CRA
~~	~~~	Abby Murano	Additional CRA
~~	~~~	Angel Zhou	Additional CRA
~~	~~~	Ashlee Young	Additional CRA
~~	~~~	Carrie Hartman	Additional CRA
~~	~~~	Carrie Lindsay	Additional CRA
~~	~~~	Chloe Sutton	Additional CRA
~~	~~~	Christine Maize	Additional CRA
~~	~~~	Craig Spencer	Additional CRA
~~	~~~	Hala ElKerdawy	Additional CRA
~~	~~~	Heather Doucette	Additional CRA
~~	~~~	Jackie Edwards	Additional CRA
~~	~~~	James Medd	Additional CRA
~~	~~~	Jennifer Pritchard	Additional CRA

INVESTIGATORS AND COLLABORATORS: SITE STAFF

Staff on site delegation logs

Country	Site	Names	Role
~~	~~~	Jessica Ferguson	Additional CRA
~~	~~~	Kristina Carmichael	Additional CRA
~~	~~~	Nancy Paul	Additional CRA
~~	~~~	Noureen Hassan	Additional CRA
~~	~~~	Stephanie Raposo	Additional CRA
~~	~~~	Stephanie Willing	Additional CRA
~~	~~~	Virginia Tennant	Additional CRA
~~	~~~	Yvonne Moelker	Additional CRA
~~	~~~	Marleen RossSmith	Pharmacist
~~	~~~	Ashley Ross	Other
~~	~~~	Mike Brander	Other
~~	~~~	Scott Bonner	Other
Canada	Kitchener, ON: Grand River Regional Cancer Centre at Grand River Hosp	Joda Kuk	Site PI
~~	~~~	Darindra Gopaul	Clinical/Surgical
~~	~~~	Jochen Knackstedt	Clinical/Surgical
~~	~~~	Ramana Rachakonda	Clinical/Surgical
~~	~~~	Ronald Hamilton	Clinical/Surgical
~~	~~~	Elyse Wellhauser	Principal CRA
~~	~~~	Kelly Walker	Principal CRA
~~	~~~	Kristin Krokoszynski	Principal CRA
~~	~~~	Susan Janke	Principal CRA
~~	~~~	Carol Ballantyne	Ethics CRA
~~	~~~	Anissa Mumin	Additional CRA
~~	~~~	Atif Siddiqui	Additional CRA
~~	~~~	Brenda S Shantz	Additional CRA
~~	~~~	Carla Girolametto	Additional CRA
~~	~~~	Carol Ballantyne	Additional CRA
~~	~~~	Debra Hendel	Additional CRA
~~	~~~	Elyse Wellhauser	Additional CRA
~~	~~~	Jennifer Gearing	Additional CRA
~~	~~~	Kelly Walker	Additional CRA
~~	~~~	Kristin Krokoszynski	Additional CRA
~~	~~~	MaryBeth Morrison	Additional CRA
~~	~~~	Sheeba Thallury	Additional CRA

INVESTIGATORS AND COLLABORATORS: SITE STAFF
Staff on site delegation logs

Country	Site	Names	Role
~~	~~~	Stephanie Nemirov	Additional CRA
Canada	London, ON: London Regional Cancer Program	George B Rodrigues	Site PI
~~	~~~	Belal Ahmad	Clinical/Surgical
~~	~~~	David DSouza	Clinical/Surgical
~~	~~~	Glenn Bauman	Clinical/Surgical
~~	~~~	Michael I Lock	Clinical/Surgical
~~	~~~	Tracy Sexton	Clinical/Surgical
~~	~~~	Varagur M Venkatesan	Clinical/Surgical
~~	~~~	Kes Sebborn	Principal CRA
~~	~~~	Christina Gurzanski	Ethics CRA
~~	~~~	Laura Bailey	Ethics CRA
~~	~~~	Mary Beth Husson	Ethics CRA
~~	~~~	Patricia Moore	Ethics CRA
~~	~~~	Shannon Kenny	Ethics CRA
~~	~~~	Albert Gratton	Additional CRA
~~	~~~	Craig Johnson	Additional CRA
~~	~~~	Danylle Corkery	Additional CRA
~~	~~~	Darlene tenHaaf	Additional CRA
~~	~~~	Elenor DiLullo	Additional CRA
~~	~~~	Heather Mayer	Additional CRA
~~	~~~	Jennifer A Hare	Additional CRA
~~	~~~	Kate Loughlin	Additional CRA
~~	~~~	Lois Eichenberger	Additional CRA
~~	~~~	Mary Beth Husson	Additional CRA
~~	~~~	Patricia Moore	Additional CRA
~~	~~~	Peggy Francis	Additional CRA
~~	~~~	Stephen Mardell	Additional CRA
~~	~~~	Susan Grant	Additional CRA
~~	~~~	Lori Sax	Pharmacist
~~	~~~	Mary Jane Camara	Other
Canada	Montreal, QC: CIUSSS de l'Est-de-I'Île de Montréal Hôpital Maisonneuve-Rosemont	Peter Vavassis	Site PI
~~	~~~	Alma Sylvestre	Clinical/Surgical
~~	~~~	Benoit Laliberte	Clinical/Surgical
~~	~~~	Celine Lemaire	Clinical/Surgical

INVESTIGATORS AND COLLABORATORS: SITE STAFF
Staff on site delegation logs

Country	Site	Names	Role
~~	~~~	David H A Nguyen	Clinical/Surgical
~~	~~~	Michael Yassa	Clinical/Surgical
~~	~~~	Nader Khaouam	Clinical/Surgical
~~	~~~	Josee AbiSaad	Principal CRA
~~	~~~	Linda RoyHuneault	Principal CRA
~~	~~~	Veronique Tran	Principal CRA
~~	~~~	Josee AbiSaad	Ethics CRA
~~	~~~	Linda RoyHuneault	Ethics CRA
~~	~~~	Veronique Tran	Ethics CRA
~~	~~~	AnneMarie Chatelain	Additional CRA
~~	~~~	Jacqueline Fortin	Additional CRA
~~	~~~	Linda RoyHuneault	Additional CRA
~~	~~~	Nicole Lachance	Additional CRA
~~	~~~	Stefanie Houle	Additional CRA
Canada	Montreal, QC: McGill University	Marie Duclos	Site PI
~~	~~~	George Shenouda	Clinical/Surgical
~~	~~~	Luis Souhami	Clinical/Surgical
~~	~~~	Sergio Luiz Faria	Clinical/Surgical
~~	~~~	MarieClaude Joncas	Principal CRA
~~	~~~	Penny Chipman	Principal CRA
~~	~~~	Rajesh Sharma	Principal CRA
~~	~~~	Brenda Lee	Ethics CRA
~~	~~~	Carolynna Olha	Ethics CRA
~~	~~~	Charlotte Corwin	Ethics CRA
~~	~~~	Linda Casey	Ethics CRA
~~	~~~	Stephanie Larocque	Ethics CRA
~~	~~~	Wayne Briand	Ethics CRA
~~	~~~	Jamila Adnaan	Additional CRA
~~	~~~	Mokhtar Ghoul	Additional CRA
~~	~~~	Zoe Koulouris	Pharmacist
~~	~~~	MarieFrance Robert	Other
Canada	Oshawa, ON: Lakeridge Health Oshawa	Wayne Koll	Site PI
~~	~~~	Audrey Li	Clinical/Surgical
~~	~~~	Fawaad Iqbal	Clinical/Surgical

INVESTIGATORS AND COLLABORATORS: SITE STAFF

Staff on site delegation logs

Country	Site	Names	Role
~~	~~~	Jimmy Mui	Clinical/Surgical
~~	~~~	Sten Myrehaug	Clinical/Surgical
~~	~~~	Ann Mueller	Principal CRA
~~	~~~	Helen Norton	Principal CRA
~~	~~~	Linda Klich	Principal CRA
~~	~~~	Virginia Albrecht	Principal CRA
~~	~~~	Edeliza Mendoza	Ethics CRA
~~	~~~	Nicole Stevens	Ethics CRA
~~	~~~	Aileen Manganaro	Additional CRA
~~	~~~	Angelina Singson	Additional CRA
~~	~~~	Ann Mueller	Additional CRA
~~	~~~	Ashane Somasiri	Additional CRA
~~	~~~	Dianne McKee	Additional CRA
~~	~~~	Helen Norton	Additional CRA
~~	~~~	Jane Froese	Additional CRA
~~	~~~	Linda Klich	Additional CRA
~~	~~~	Shannon Kift	Additional CRA
~~	~~~	Janet Slessor	Pharmacist
~~	~~~	Barbara MacGregor	Other
~~	~~~	Nancy Froude	Other
~~	~~~	Pamela Howitt	Other
Canada	Ottawa, ON: Ottawa Hosp Research Institute	Shawn Malone	Site PI
~~	~~~	Alain Haddad	Clinical/Surgical
~~	~~~	Choan E	Clinical/Surgical
~~	~~~	Gad A Perry	Clinical/Surgical
~~	~~~	JeanMarc Bourque	Clinical/Surgical
~~	~~~	Libni Eapen	Clinical/Surgical
~~	~~~	Robert M MacRae	Clinical/Surgical
~~	~~~	Scott Morgan	Clinical/Surgical
~~	~~~	Wayne S Kendal	Clinical/Surgical
~~	~~~	Caroline Proulx	Principal CRA
~~	~~~	Margaret MacGillivray	Principal CRA
~~	~~~	Scott Grimes	Principal CRA
~~	~~~	Stella Park	Principal CRA

INVESTIGATORS AND COLLABORATORS: SITE STAFF
Staff on site delegation logs

Country	Site	Names	Role
~~	~~~	Archana Tikoo	Ethics CRA
~~	~~~	Elina Iordanidi	Ethics CRA
~~	~~~	Femina Kanji	Ethics CRA
~~	~~~	Julie Nguyen	Ethics CRA
~~	~~~	Koralee Berghout	Ethics CRA
~~	~~~	Lisa Turriff	Ethics CRA
~~	~~~	Maria Sanchez	Ethics CRA
~~	~~~	MarieClaude Reeves	Ethics CRA
~~	~~~	Mary Spearman	Ethics CRA
~~	~~~	Matt Fish	Ethics CRA
~~	~~~	Nancy Eze	Ethics CRA
~~	~~~	Amy Carkner	Additional CRA
~~	~~~	Anna OBrien	Additional CRA
~~	~~~	Caroline Proulx	Additional CRA
~~	~~~	Joanne Roach	Additional CRA
~~	~~~	Kimberly Hicks	Additional CRA
~~	~~~	Lisa MacMullin	Additional CRA
~~	~~~	Margaret MacGillivray	Additional CRA
~~	~~~	Nancy Page	Additional CRA
~~	~~~	Natalie Wright	Additional CRA
~~	~~~	Patti Spencer	Additional CRA
~~	~~~	Scott Grimes	Additional CRA
~~	~~~	Stella Park	Additional CRA
Canada	Quebec City, QC: Hotel-Dieu de Quebec	Eric Vigneault	Site PI
~~	~~~	AndreGuy Martin	Clinical/Surgical
~~	~~~	JoseLuis Gomez	Clinical/Surgical
~~	~~~	Lara Hathout	Clinical/Surgical
~~	~~~	Leonello Cusan	Clinical/Surgical
~~	~~~	Luis Diaz de Bedoya	Clinical/Surgical
~~	~~~	William Foster	Clinical/Surgical
~~	~~~	Josee Allard	Principal CRA
~~	~~~	Isabelle Desrosiers	Ethics CRA
~~	~~~	Josee Allard	Ethics CRA
~~	~~~	Danielle Cossette	Additional CRA

INVESTIGATORS AND COLLABORATORS: SITE STAFF
Staff on site delegation logs

Country	Site	Names	Role
~~	~~~	Josee Allard	Additional CRA
~~	~~~	MarieAndree Lajoie	Additional CRA
~~	~~~	Nathalie Dufour	Additional CRA
~~	~~~	Sophie Pouliot	Additional CRA
Canada	Sherbrooke, QC: CIUSSS de l'Estrie - Centre hospitalier universitaire de Sherbrooke	Abdenour Nabid	Site PI
~~	~~~	Annie Ebacher	Clinical/Surgical
~~	~~~	Audrey TetreaultLaflamme	Clinical/Surgical
~~	~~~	Isabelle C Gauthier	Clinical/Surgical
~~	~~~	Michel Carmel	Clinical/Surgical
~~	~~~	Myriam Bouchard	Clinical/Surgical
~~	~~~	Rachel Bujold	Clinical/Surgical
~~	~~~	Robert Sabbagh	Clinical/Surgical
~~	~~~	Sophie Couture	Principal CRA
~~	~~~	Sophie Couture	Ethics CRA
~~	~~~	Alexandra Lamoureux	Additional CRA
~~	~~~	Cynthia Ladouceur	Additional CRA
~~	~~~	Diane Pearson	Additional CRA
~~	~~~	Genevieve Cote	Additional CRA
~~	~~~	Helene Dion	Additional CRA
~~	~~~	Julie Larouche	Additional CRA
~~	~~~	Tania Marquis	Additional CRA
Canada	Toronto, ON: Dr. H. Bliss Murphy Cancer Centre	Jinka R Sathya	Site PI
~~	~~~	John Thoms	Site PI
~~	~~~	Alia Norman	Clinical/Surgical
~~	~~~	Asim Kamran	Clinical/Surgical
~~	~~~	Craig Pochini	Clinical/Surgical
~~	~~~	Jonathan Greenland	Clinical/Surgical
~~	~~~	Allison Joy	Principal CRA
~~	~~~	Elysia Desai	Principal CRA
~~	~~~	Kelli Mitchell	Principal CRA
~~	~~~	Arifur Rahman	Ethics CRA
~~	~~~	Dawne Putt	Ethics CRA
~~	~~~	Erin Baker	Ethics CRA
~~	~~~	Lauren Rickert	Ethics CRA

INVESTIGATORS AND COLLABORATORS: SITE STAFF

Staff on site delegation logs

Country	Site	Names	Role
~~	~~~	Liz Fuller	Ethics CRA
~~	~~~	Matthew Nelder	Ethics CRA
~~	~~~	Stacy Whittle	Ethics CRA
~~	~~~	Allison Joy	Additional CRA
~~	~~~	Ashley Hoskins	Additional CRA
~~	~~~	Breanne Teasdale	Additional CRA
~~	~~~	Chrystal Whiteway	Additional CRA
~~	~~~	Denise Galway	Additional CRA
~~	~~~	Elysia Desai	Additional CRA
~~	~~~	Erin Pinnell	Additional CRA
~~	~~~	Gail House	Additional CRA
~~	~~~	Geoffrey Blackwood	Additional CRA
~~	~~~	Jamie ODea	Additional CRA
~~	~~~	Kelli Mitchell	Additional CRA
~~	~~~	Kimberley Manning	Additional CRA
~~	~~~	Liz Fuller	Additional CRA
~~	~~~	Lorilee Noel	Additional CRA
~~	~~~	Zeta Hannaford	Pharmacist
Canada	Toronto, ON: Odette Cancer Centre	Hans T Chung	Site PI
~~	~~~	Cyril Danjoux	Clinical/Surgical
~~	~~~	Danny Vesprini	Clinical/Surgical
~~	~~~	Douglas Andrew Loblaw	Clinical/Surgical
~~	~~~	Eric Chia Lin Tseng	Clinical/Surgical
~~	~~~	Ewa Szumacher	Clinical/Surgical
~~	~~~	Gerard Morton	Clinical/Surgical
~~	~~~	Laurence Klotz	Clinical/Surgical
~~	~~~	Patrick CF Cheung	Clinical/Surgical
~~	~~~	Robert K Nam	Clinical/Surgical
~~	~~~	Stanley Liu	Clinical/Surgical
~~	~~~	William Chu	Clinical/Surgical
~~	~~~	Anam Shahid	Principal CRA
~~	~~~	Andrea DeAbreu	Principal CRA
~~	~~~	Arlynne Marquez	Principal CRA
~~	~~~	Kristina Commisso	Principal CRA

INVESTIGATORS AND COLLABORATORS: SITE STAFF

Staff on site delegation logs

Country	Site	Names	Role
~~	~~~	Senny Chan	Principal CRA
~~	~~~	Vivette Escueta	Principal CRA
~~	~~~	Andrea DeAbreu	Ethics CRA
~~	~~~	Arlynne Marquez	Ethics CRA
~~	~~~	Kristina Commisso	Ethics CRA
~~	~~~	Senny Chan	Ethics CRA
~~	~~~	Zeeba Bhounr	Ethics CRA
~~	~~~	Anam Shahid	Additional CRA
~~	~~~	Andrea DeAbreu	Additional CRA
~~	~~~	Angela Commisso	Additional CRA
~~	~~~	Arlynne Marquez	Additional CRA
~~	~~~	Leila Malek	Additional CRA
~~	~~~	Zeeba Bhounr	Additional CRA
~~	~~~	Michael Leung	Pharmacist
Canada	Toronto, ON: University Health Network Princess Margaret Cancer Centre	Charles Catton	Site PI
~~	~~~	Alejandro Berlin	Clinical/Surgical
~~	~~~	Andrew J Bayley	Clinical/Surgical
~~	~~~	Cynthia Menard	Clinical/Surgical
~~	~~~	Joelle Helou	Clinical/Surgical
~~	~~~	Mary Gospodarowicz	Clinical/Surgical
~~	~~~	Michael Milosevic	Clinical/Surgical
~~	~~~	Nafisha Lalani	Clinical/Surgical
~~	~~~	Padraig Warde	Clinical/Surgical
~~	~~~	Peter Chung	Clinical/Surgical
~~	~~~	Robert Bristow	Clinical/Surgical
~~	~~~	Srinivas Raman	Clinical/Surgical
~~	~~~	Andrei Rotarescu	Principal CRA
~~	~~~	Evan Strom	Principal CRA
~~	~~~	Ida Lee	Principal CRA
~~	~~~	Mary Tannourji	Principal CRA
~~	~~~	Melissa Lem	Principal CRA
~~	~~~	Navneet Arora	Principal CRA
~~	~~~	Suzana Djuric	Principal CRA
~~	~~~	Andrei Rotarescu	Ethics CRA

INVESTIGATORS AND COLLABORATORS: SITE STAFF
Staff on site delegation logs

Country	Site	Names	Role
~~	~~~	Evan Strom	Ethics CRA
~~	~~~	Ida Lee	Ethics CRA
~~	~~~	Mary Tannourji	Ethics CRA
~~	~~~	Melissa Lem	Ethics CRA
~~	~~~	Navneet Arora	Ethics CRA
~~	~~~	Suzana Djuric	Ethics CRA
~~	~~~	Aleksandra Petrovska	Additional CRA
~~	~~~	Aleksandra Topalovich	Additional CRA
~~	~~~	Amadeus Chui	Additional CRA
~~	~~~	Andrei Rotarescu	Additional CRA
~~	~~~	Arundhati Shukla	Additional CRA
~~	~~~	Avi Petroff	Additional CRA
~~	~~~	Bernadeth Lao	Additional CRA
~~	~~~	Cynthia Torres	Additional CRA
~~	~~~	Daeria Lawson	Additional CRA
~~	~~~	Daniel Ang	Additional CRA
~~	~~~	Debbie Tsuji	Additional CRA
~~	~~~	Emily Gregorcic	Additional CRA
~~	~~~	Erin Velec	Additional CRA
~~	~~~	Evan Strom	Additional CRA
~~	~~~	Heidi Chan	Additional CRA
~~	~~~	Ida Lee	Additional CRA
~~	~~~	Jessy Abed	Additional CRA
~~	~~~	John Hsien	Additional CRA
~~	~~~	Judy Quintos	Additional CRA
~~	~~~	Karen Tse	Additional CRA
~~	~~~	Kathryn Sabate	Additional CRA
~~	~~~	Kris Wallace	Additional CRA
~~	~~~	Kyoko Tiessen	Additional CRA
~~	~~~	Maria Jose Gaitan Ruiz	Additional CRA
~~	~~~	Mary Tannourji	Additional CRA
~~	~~~	Nanthini Tharahan	Additional CRA
~~	~~~	Nicole Gumapac	Additional CRA
~~	~~~	Pat Merante	Additional CRA

INVESTIGATORS AND COLLABORATORS: SITE STAFF

Staff on site delegation logs

Country	Site	Names	Role
~~	~~~	Ramesh Mirmooji	Additional CRA
~~	~~~	Sandra Pineda	Additional CRA
~~	~~~	Sarah Degendorfer	Additional CRA
~~	~~~	Sarah Ramotar	Additional CRA
~~	~~~	Sarin Peres	Additional CRA
~~	~~~	Suzana Djuric	Additional CRA
~~	~~~	Vaishaali Thevarajah	Additional CRA
~~	~~~	Vickie Kong	Additional CRA
~~	~~~	Zaynab Muraj	Additional CRA
Canada	Trois-Rivieres, QC: Centre hospitalier regional de Trois-Rivieres	Francois Vincent	Site PI
~~	~~~	Benoit Lebrun	Clinical/Surgical
~~	~~~	Julie Harvey	Clinical/Surgical
~~	~~~	LindaSuzanne Vincent	Clinical/Surgical
~~	~~~	MarieEve Sicard	Clinical/Surgical
~~	~~~	Rafika Dahmane	Clinical/Surgical
~~	~~~	MarieEve Caron	Principal CRA
~~	~~~	MarieEve Caron	Ethics CRA
~~	~~~	Andreanne Thibeault	Additional CRA
~~	~~~	Julie Samson	Additional CRA
~~	~~~	Line Marineau	Additional CRA
~~	~~~	MarieEve Bisson	Additional CRA
~~	~~~	Vanessa Gagne	Additional CRA
~~	~~~	Julie Montour	Pharmacist
Canada	Vancouver, BC: Vancouver Cancer Centre	Abraham S Alexander	Site PI
~~	~~~	Jennifer Goulart	Site PI
~~	~~~	Abraham S Alexander	Clinical/Surgical
~~	~~~	Caroline Holloway	Clinical/Surgical
~~	~~~	Daniel Glick	Clinical/Surgical
~~	~~~	Emilie Priestley	Clinical/Surgical
~~	~~~	Howard H Pai	Clinical/Surgical
~~	~~~	Howard Joe	Clinical/Surgical
~~	~~~	Isabelle Vallieres	Clinical/Surgical
~~	~~~	Jacqueline Lam	Clinical/Surgical
~~	~~~	Jan Lim	Clinical/Surgical

INVESTIGATORS AND COLLABORATORS: SITE STAFF

Staff on site delegation logs

Country	Site	Names	Role
~~	~~~	Jennifer Goulart	Clinical/Surgical
~~	~~~	Joycelin Canavan	Clinical/Surgical
~~	~~~	Negin Shahid	Clinical/Surgical
~~	~~~	Nelson Leong	Clinical/Surgical
~~	~~~	Paul A Blood	Clinical/Surgical
~~	~~~	Emily White	Principal CRA
~~	~~~	Heather Lockyer	Principal CRA
~~	~~~	Michael A Miller	Principal CRA
~~	~~~	Sally Hodgson	Principal CRA
~~	~~~	Sarah Irons	Principal CRA
~~	~~~	Senz Hamilton	Principal CRA
~~	~~~	Emily White	Ethics CRA
~~	~~~	Heather Lockyer	Ethics CRA
~~	~~~	Michael A Miller	Ethics CRA
~~	~~~	Sally Hodgson	Ethics CRA
~~	~~~	Sarah Irons	Ethics CRA
~~	~~~	Senz Hamilton	Ethics CRA
~~	~~~	Alia Lomas	Additional CRA
~~	~~~	Cathy Lacey	Additional CRA
~~	~~~	Eleanor Holwerda	Additional CRA
~~	~~~	Heather Lockyer	Additional CRA
~~	~~~	Mia AnnaMaria Clements	Additional CRA
~~	~~~	Michael A Miller	Additional CRA
~~	~~~	Senz Hamilton	Additional CRA
Canada	Victoria, BC: Vancouver Island Cancer Centre	Charmaine KimSing	Site PI
~~	~~~	Alan I So	Clinical/Surgical
~~	~~~	Graeme Duncan	Clinical/Surgical
~~	~~~	Martin E Gleave	Clinical/Surgical
~~	~~~	Michael McKenzie	Clinical/Surgical
~~	~~~	Michael Peacock	Clinical/Surgical
~~	~~~	Mira Keyes	Clinical/Surgical
~~	~~~	S Larry Goldenberg	Clinical/Surgical
~~	~~~	Scott Tyldesley	Clinical/Surgical
~~	~~~	Sree Rodda	Clinical/Surgical

INVESTIGATORS AND COLLABORATORS: SITE STAFF

Staff on site delegation logs

Country	Site	Names	Role
~~	~~~	Thomas A Pickles	Clinical/Surgical
~~	~~~	Tina Wanting Zhang	Clinical/Surgical
~~	~~~	William James Morris	Clinical/Surgical
~~	~~~	Dana Matuszewski	Principal CRA
~~	~~~	Devon Poznanski	Principal CRA
~~	~~~	Lejla Gavranovic	Principal CRA
~~	~~~	Lorenz Yeung	Principal CRA
~~	~~~	Sandy Chang	Principal CRA
~~	~~~	Dana Matuszewski	Ethics CRA
~~	~~~	Devon Poznanski	Ethics CRA
~~	~~~	Lejla Gavranovic	Ethics CRA
~~	~~~	Lorenz Yeung	Ethics CRA
~~	~~~	Sandy Chang	Ethics CRA
~~	~~~	Sheena Sibug	Additional CRA
~~	~~~	Subashini Karunakaran	Additional CRA
~~	~~~	Lynne Nakashima	Pharmacist
Canada	Winnipeg, MB: CancerCare Manitoba	Aldrich Dixon Ong	Site PI
~~	~~~	Amitava D Chowdhury	Clinical/Surgical
~~	~~~	Arbind Dubey	Clinical/Surgical
~~	~~~	Atul Sharma	Clinical/Surgical
~~	~~~	Bashir Bashir	Clinical/Surgical
~~	~~~	Darrel E Drachenberg	Clinical/Surgical
~~	~~~	David Dawe	Clinical/Surgical
~~	~~~	Gokulan Sivananthan	Clinical/Surgical
~~	~~~	Hanbo Zhang	Clinical/Surgical
~~	~~~	Harvey Quon	Clinical/Surgical
~~	~~~	Jeffrey Graham	Clinical/Surgical
~~	~~~	Maged Nashed	Clinical/Surgical
~~	~~~	Rashmi Koul	Clinical/Surgical
~~	~~~	Shahida Ahmed	Clinical/Surgical
~~	~~~	William Hunter	Clinical/Surgical
~~	~~~	Amber DelisleCorps	Principal CRA
~~	~~~	Chinedu Ifeanyi	Principal CRA
~~	~~~	Christopher Schroeder	Principal CRA

INVESTIGATORS AND COLLABORATORS: SITE STAFF
Staff on site delegation logs

Country	Site	Names	Role
~~	~~~	Elizabeth Lylyk	Principal CRA
~~	~~~	Heidi Kampen	Principal CRA
~~	~~~	Maggie Putnam	Principal CRA
~~	~~~	Megan Ridler	Principal CRA
~~	~~~	Melissa Bridges	Principal CRA
~~	~~~	Sandy Yap	Principal CRA
~~	~~~	Swati Singla	Principal CRA
~~	~~~	Tianna Mohammed	Principal CRA
~~	~~~	Amber DelisleCorps	Ethics CRA
~~	~~~	Chinedu Ifeanyi	Ethics CRA
~~	~~~	Christopher Schroeder	Ethics CRA
~~	~~~	Christy Turner	Ethics CRA
~~	~~~	Darlene Zwarych	Ethics CRA
~~	~~~	DeepKumar Patel	Ethics CRA
~~	~~~	Elizabeth Lylyk	Ethics CRA
~~	~~~	Heidi Kampen	Ethics CRA
~~	~~~	Jennifer McNish	Ethics CRA
~~	~~~	Jill Jacinto	Ethics CRA
~~	~~~	JoAnn Weir	Ethics CRA
~~	~~~	Joyce Wei	Ethics CRA
~~	~~~	Laurel Johnston	Ethics CRA
~~	~~~	Leah Bergen	Ethics CRA
~~	~~~	Lovely Cadiz	Ethics CRA
~~	~~~	Maggie Putnam	Ethics CRA
~~	~~~	Megan Ridler	Ethics CRA
~~	~~~	Melissa Bridges	Ethics CRA
~~	~~~	Raquel Gamez	Ethics CRA
~~	~~~	Sandy Yap	Ethics CRA
~~	~~~	Swati Singla	Ethics CRA
~~	~~~	Theresa Moore	Ethics CRA
~~	~~~	Tianna Mohammed	Ethics CRA
~~	~~~	Allison Wood	Additional CRA
~~	~~~	Arpita Majumdar	Additional CRA
~~	~~~	Ashley Ouelette	Additional CRA

INVESTIGATORS AND COLLABORATORS: SITE STAFF
Staff on site delegation logs

Country	Site	Names	Role
~~	~~~	Barb Ammeter	Additional CRA
~~	~~~	Caitlin Kitkowski	Additional CRA
~~	~~~	Cristina Francisco	Additional CRA
~~	~~~	Debbie Last	Additional CRA
~~	~~~	Gina Garrett	Additional CRA
~~	~~~	Heather Davies	Additional CRA
~~	~~~	Heather Long	Additional CRA
~~	~~~	Heidi Kampen	Additional CRA
~~	~~~	Jacqueline Chahine	Additional CRA
~~	~~~	Jamie Vaughn	Additional CRA
~~	~~~	Jill Jacinto	Additional CRA
~~	~~~	Joelle DuMontier	Additional CRA
~~	~~~	Julia Giovannini	Additional CRA
~~	~~~	Kathi Klapp	Additional CRA
~~	~~~	Kathleen Marek	Additional CRA
~~	~~~	Kathy Cherepak	Additional CRA
~~	~~~	Kathy Trakalo	Additional CRA
~~	~~~	KuanLin Li	Additional CRA
~~	~~~	Laurie Mills	Additional CRA
~~	~~~	Leanne Anderson	Additional CRA
~~	~~~	Lisa Peak	Additional CRA
~~	~~~	Lori Walker	Additional CRA
~~	~~~	Mandy Squires	Additional CRA
~~	~~~	Maria Morales	Additional CRA
~~	~~~	Marilyn Pawl	Additional CRA
~~	~~~	Megan Ridler	Additional CRA
~~	~~~	Melanie R Watson	Additional CRA
~~	~~~	Patricia Benjaminson	Additional CRA
~~	~~~	Priscilla Santos	Additional CRA
~~	~~~	Rayleen Rudnicki	Additional CRA
~~	~~~	Rhonda Nichol	Additional CRA
~~	~~~	Robyn Guarino	Additional CRA
~~	~~~	Rose Woloshyn	Additional CRA
~~	~~~	Sandy Yap	Additional CRA

INVESTIGATORS AND COLLABORATORS: SITE STAFF
Staff on site delegation logs

Country	Site	Names	Role
~~	~~~	Shauna Priebe	Additional CRA
~~	~~~	Stacey Rizzuto	Additional CRA
~~	~~~	Taehee Ann	Additional CRA
~~	~~~	Theresa Moore	Additional CRA
~~	~~~	Tiffani Eska	Additional CRA
~~	~~~	Tracy Chornopyski	Additional CRA
~~	~~~	Valerie Kuzyk	Additional CRA
~~	~~~	Zeljka Bakija	Additional CRA
Denmark	Aalborg: Aalborg University Hosp	Ms Mette Moe Kempel	Site PI
~~	~~~	Mr Niels Harving	Clinical/Surgical
~~	~~~	Mr Finn Hejlesen	Point of contact (1st)
~~	~~~	Ms Kirsten Steffensen	Research Nurse
Denmark	Aarhus: Aarhus Kommunehospital	Dr Simon Buus	Site PI
~~	~~~	Dr Henrik Schultz	Clinical/Surgical
~~	~~~	Dr Henrik Schultz	Clinical/Surgical
~~	~~~	Dr Kirsten Fode	Clinical/Surgical
~~	~~~	Dr Lise Bentzen	Clinical/Surgical
~~	~~~	Dr Michael Borre	Clinical/Surgical
~~	~~~	Dr Slavka Lucakova	Clinical/Surgical
~~	~~~	Dr Yasmin Lassen	Clinical/Surgical
~~	~~~	Ms Helle Lemng Kruse	Point of contact (1st)
~~	~~~	Ms Helle Nørmark Iversen	Point of contact (1st)
~~	~~~	Ms Vibeke Laursen	Point of contact (1st)
~~	~~~	Ms Birgit Kaa Bach	Research Nurse
Denmark	Copenhagen: Rigshospitalet University Hosp	Dr Peter Meidhal Petersen	Site PI
~~	~~~	Dr Anne Juel Christensen	Clinical/Surgical
~~	~~~	Prof Klaus Brasso	Clinical/Surgical
~~	~~~	Ms Anne Sofie Lunau	Point of contact (1st)
~~	~~~	Ms Sine Stilling	Point of contact (1st)
~~	~~~	Ms Stine Thim	Point of contact (1st)
~~	~~~	Ms Karina Klovgaard	Point of contact (2nd)
~~	~~~	Ms Anna Torin Lehmann	Research Nurse
~~	~~~	Ms Lisa Gruschy	Research Nurse
~~	~~~	Ms Lone Lund Poder	Research Nurse

INVESTIGATORS AND COLLABORATORS: SITE STAFF
Staff on site delegation logs

Country	Site	Names	Role
~~	~~~	Ms Maria Vandborg	Research Nurse
~~	~~~	Ms Trine Moller Christensen	Research Nurse
~~	~~~	Ms Kristine Ribers	Other
Denmark	Herlev: Amtssygehuset i Herlev (Herlev University Hosp)	Dr Henriette Lindberg	Site PI
~~	~~~	Ms Kathrine Friser Kokholm	Point of contact (1st)
~~	~~~	Ms Tine Christensen	Point of contact (1st)
~~	~~~	Ms Charlotte Saxe	Trial Coordinator
~~	~~~	Ms Eva Rønnengart	Trial Coordinator
~~	~~~	Kirstine Nybom	Other
Ireland	Galway: University College Hosp Galway	Mr Garret Durkan	Site PI
~~	~~~	Dr Joseph Martin	Clinical/Surgical
~~	~~~	Mr Eamonn Rogers	Clinical/Surgical
~~	~~~	Prof Frank Sullivan	Clinical/Surgical
~~	~~~	Ms Chiaw Woon Teh	Point of contact (1st)
~~	~~~	Ms Marian Jennings	Point of contact (1st)
~~	~~~	Ms Ann Wright	Point of contact (2nd)
~~	~~~	Ms Catriona Mahoney	Research Nurse
~~	~~~	Ms Liz Raftery	Research Nurse
~~	~~~	Ms Mary Marg Byrne	Research Nurse
~~	~~~	Ms Veronica McInerney	Research Nurse
~~	~~~	Ms Emma Deenihan	Administrator
~~	~~~	Ms Maria Spillane	Other
UK	Aberdeen: Aberdeen Royal Inf	Dr Graham MacDonald	Site PI
~~	~~~	Dr Donald Bissett	Clinical/Surgical
~~	~~~	Dr Judith Grant	Clinical/Surgical
~~	~~~	Mr Rory Lynch	Point of contact (1st)
~~	~~~	Mrs Rachel Moir	Point of contact (1st)
~~	~~~	Ms Marie McWilliam	Point of contact (1st)
~~	~~~	Ms Kirsty Shearer	Point of contact (2nd)
~~	~~~	Mrs Margaret Smith	Research Nurse
~~	~~~	Ms Sue Rodwell	Research Nurse
~~	~~~	Ms Shelagh Bonner-Shand	Trial Coordinator
UK	Airedale: Airedale General Hosp	Dr Ganesan Jeyasanger	Site PI
~~	~~~	Dr Ann Henry	Clinical/Surgical

INVESTIGATORS AND COLLABORATORS: SITE STAFF
Staff on site delegation logs

Country	Site	Names	Role
~~	~~~	Dr Nathalie Casanova	Clinical/Surgical
~~	~~~	Dr Simon Brown	Clinical/Surgical
~~	~~~	Mrs Alison Shaw	Point of contact (1st)
~~	~~~	Mrs Pipa Hill	Point of contact (1st)
~~	~~~	Ms Helen Henson	Point of contact (1st)
~~	~~~	Ms Maxine Briggs	Point of contact (2nd)
~~	~~~	Mrs Sharron Parkinson	Research Nurse
~~	~~~	Ms Judy McAlister	Research Nurse
~~	~~~	Ms Louise Binns	Research Nurse
~~	~~~	Mrs Jasmine Hartley	Research Asst
~~	~~~	Dr Katy Clark	Other
UK	Aylesbury: Stoke Mandeville Hosp	Dr Katherine Hyde	Site PI
~~	~~~	Dr Ami Sabharwal	Clinical/Surgical
~~	~~~	Dr Andrew Weaver	Clinical/Surgical
~~	~~~	Dr Christopher Alcock	Clinical/Surgical
~~	~~~	Dr Joanne Brady	Clinical/Surgical
~~	~~~	Dr Niki Panakis	Clinical/Surgical
~~	~~~	Dr Philip Camilleri	Clinical/Surgical
~~	~~~	Dr Thinn Pwint	Clinical/Surgical
~~	~~~	Dr Janice Carpenter	Point of contact (1st)
~~	~~~	Mrs Alice Ngumo	Point of contact (1st)
~~	~~~	Mrs Cheryl Padilla-Harris	Research Nurse
~~	~~~	Mrs Gail Varley	Research Nurse
~~	~~~	Mrs Tracey Stammers	Research Nurse
~~	~~~	Ms Emma Hogbin	Research Nurse
~~	~~~	Ms Hazel Wynn	Research Nurse
~~	~~~	Ms Helena Stone	Research Nurse
~~	~~~	Ms Roisin Kavanagh	Pharmacist
UK	Ayr: Ayr Hosp	Dr Nicholas Macleod	Site PI
~~	~~~	Dr Aisha Tufail	Clinical/Surgical
~~	~~~	Dr Aqilah Othman	Clinical/Surgical
~~	~~~	Dr Hilary Glen	Clinical/Surgical
~~	~~~	Dr Janet Graham	Clinical/Surgical
~~	~~~	Dr Jawaher Ansari	Clinical/Surgical

INVESTIGATORS AND COLLABORATORS: SITE STAFF
Staff on site delegation logs

Country	Site	Names	Role
~~	~~~	Dr Maryon Hardie	Clinical/Surgical
~~	~~~	Dr Nick Mcleod	Clinical/Surgical
~~	~~~	Dr Patricia Roxburgh	Clinical/Surgical
~~	~~~	Dr Rana Mahmood	Clinical/Surgical
~~	~~~	Miss Kristy Ross	Clinical/Surgical
~~	~~~	Ms Claudia Coubrough	Point of contact (1st)
~~	~~~	Ms Claudia Turley	Point of contact (1st)
~~	~~~	Mr Mark Wilson	Research Nurse
~~	~~~	Mrs Jane McClements	Research Nurse
~~	~~~	Ms Chloe Cowan	Research Nurse
~~	~~~	Ms Margaret McKernan	Research Nurse
~~	~~~	Ms Maureen Templeton	Research Nurse
~~	~~~	Mr Philip Cannon	Trial Coordinator
~~	~~~	Ms Clare Love	Data Manager
~~	~~~	Ms Elaine Allan	Administrator
~~	~~~	Mr Brian McGlynn	Other
~~	~~~	Mr Ross Carruthers	Other
UK	Bangor: Ysbyty Gwynedd	Dr Nikhil Oommen	Site PI
~~	~~~	Dr Rachel Williams	Clinical/Surgical
~~	~~~	Dr Thomas Coventry	Clinical/Surgical
~~	~~~	Dr Zulfiqer Ali	Clinical/Surgical
~~	~~~	Mr Gareth Jones	Point of contact (1st)
~~	~~~	Ms Dianne Thomas	Point of contact (1st)
~~	~~~	Mrs Caryl Butterworth	Research Nurse
~~	~~~	Ms Rebecca Burns	Research Nurse
~~	~~~	Ms Wendy Saxton	Research Nurse
~~	~~~	Sister Hayley Tapping	Research Nurse
~~	~~~	Mr Sion Lewis	Trial Coordinator
~~	~~~	Mrs Angela Evans	Trial Coordinator
~~	~~~	Miss Kelly Andrews	Administrator
~~	~~~	Miss Rachel Thomas	Administrator
~~	~~~	Ms Beth Walker	MDT coordinator
~~	~~~	Ms Susan Owen	Radiographer
UK	Barnstaple: North Devon District Hosp	Dr Mohini Varughese	Site PI

INVESTIGATORS AND COLLABORATORS: SITE STAFF

Staff on site delegation logs

Country	Site	Names	Role
~~	~~~	Dr Anne McCormack	Clinical/Surgical
~~	~~~	Dr Denise Sheehan	Clinical/Surgical
~~	~~~	Dr Elizabeth Kershaw	Clinical/Surgical
~~	~~~	Dr Elizabeth Toy	Clinical/Surgical
~~	~~~	Dr Maria Martinez	Clinical/Surgical
~~	~~~	Dr Victoria Ford	Clinical/Surgical
~~	~~~	Ms Becky Holbrook	Point of contact (1st)
~~	~~~	Mrs Susan Collard	Research Nurse
~~	~~~	Mrs Samantha Ley	Trial Coordinator
~~	~~~	Ms Frances Goodhind	Pharmacist
~~	~~~	Mrs Lynne Van-Koutrik	Other
UK	Basingstoke: Basingstoke & North Hampshire Hosp	Dr Richard Shaffer	Site PI
~~	~~~	Dr Sangeeta Paisey	Site PI
~~	~~~	Dr Hilawati Yusof	Clinical/Surgical
~~	~~~	Dr Katherine Aitken	Clinical/Surgical
~~	~~~	Dr Rosalyne Westley	Clinical/Surgical
~~	~~~	Dr Teresa Guerrero-Urbano	Clinical/Surgical
~~	~~~	Beata Krysta	Point of contact (1st)
~~	~~~	Mrs Abigail Edwards	Point of contact (1st)
~~	~~~	Mr Godfrey Bownie-Mukumbu	Point of contact (2nd)
~~	~~~	Miss Rachel Bryan	Research Nurse
~~	~~~	Mrs Jackie Smith	Research Nurse
~~	~~~	Ms Liz Happle	Research Nurse
~~	~~~	Mrs Adrienn Fazekasne Fulep	Trial Coordinator
~~	~~~	Dr David Barlow	Data Manager
~~	~~~	Miss Louise Beattie	Data Manager
~~	~~~	Ms Victoria Corner	Research Asst
~~	~~~	Ms Julie Gwilt	Administrator
~~	~~~	Ms Dee Jackman	MDT coordinator
UK	Bath: Royal United Hosp	Dr Olivera Frim	Site PI
~~	~~~	Dr Abigail Jenner	Clinical/Surgical
~~	~~~	Dr Christine Elwell	Clinical/Surgical
~~	~~~	Dr Hugh Newman	Clinical/Surgical
~~	~~~	Dr Jessica Mason	Clinical/Surgical

INVESTIGATORS AND COLLABORATORS: SITE STAFF
Staff on site delegation logs

Country	Site	Names	Role
~~	~~~	Dr Mark Beresford	Clinical/Surgical
~~	~~~	Dr Susan Masson	Clinical/Surgical
~~	~~~	Dr Zoe Hudson	Clinical/Surgical
~~	~~~	Mr Jonathan McFarlane	Clinical/Surgical
~~	~~~	Mr Mark Mantle	Clinical/Surgical
~~	~~~	Miss Abigail Pocock	Point of contact (1st)
~~	~~~	Mrs Samantha Curtis	Point of contact (1st)
~~	~~~	Ms Bryony Robertson	Point of contact (2nd)
~~	~~~	Mrs Beatrice Hamilton	Research Nurse
~~	~~~	Mrs Christine Cox	Research Nurse
~~	~~~	Mrs Rowan Appleby	Research Nurse
~~	~~~	Mrs Ruth Brydon-Hill	Research Nurse
~~	~~~	Ms Rebecca Wassall	Research Nurse
~~	~~~	Sister Tania Williams (Née Allen)	Research Nurse
~~	~~~	Ms Eve Tomlinson	Administrator
~~	~~~	Miss Joanne Avis	Other
UK	Belfast: Belfast City Hosp	Prof Joe O'Sullivan	Site PI
~~	~~~	Dr Aiden Cole	Clinical/Surgical
~~	~~~	Dr Darren Mitchell	Clinical/Surgical
~~	~~~	Dr David Stewart	Clinical/Surgical
~~	~~~	Dr Fionnuala Houghton	Clinical/Surgical
~~	~~~	Dr Jackie Harney	Clinical/Surgical
~~	~~~	Dr Jonathan McAleese	Clinical/Surgical
~~	~~~	Dr Poh Lin Shum	Clinical/Surgical
~~	~~~	Dr Ruth Johnston	Clinical/Surgical
~~	~~~	Dr Stephen Stranex	Clinical/Surgical
~~	~~~	Dr Suneil Jain	Clinical/Surgical
~~	~~~	Mr Chris Hagan	Clinical/Surgical
~~	~~~	Mr Nambi Rajan	Clinical/Surgical
~~	~~~	Mr Patrick Keane	Clinical/Surgical
~~	~~~	Mr Peter Clarke	Point of contact (1st)
~~	~~~	Mrs Gail Gilchrist	Point of contact (2nd)
~~	~~~	Mrs Grace Totten	Point of contact (2nd)
~~	~~~	Ms Emma Hanna	Data Manager

INVESTIGATORS AND COLLABORATORS: SITE STAFF
Staff on site delegation logs

Country	Site	Names	Role
~~	~~~	Miss Jane Cousins	Radiographer
~~	~~~	Miss Karen McKenna	Radiographer
~~	~~~	Miss Stacey Murray	Radiographer
~~	~~~	Mrs Stacey Hetherington	Radiographer
~~	~~~	Ms Grace Brown	Radiographer
~~	~~~	Dr Ciara Lyons	Other
~~	~~~	Mrs Jo McAllister	Other
~~	~~~	Ms Sharon Hynds	Other
UK	Birmingham: Birmingham Heartlands Hosp	Dr Anjali Zarkar	Site PI
~~	~~~	Mrs Kavitha Shetty	Clinical/Surgical
~~	~~~	Ms Rachel Lokes	Point of contact (1st)
~~	~~~	Ms Sundip Sohanpal	Point of contact (1st)
~~	~~~	Mrs Madhura Chandrashekara	Point of contact (2nd)
~~	~~~	Ms Ann Schumacher	Point of contact (2nd)
~~	~~~	Mrs Ellen Drew	Research Nurse
~~	~~~	Mrs Tina Gamble	Research Nurse
~~	~~~	Ms Mary (Ellen) Drew	Research Nurse
~~	~~~	Mr James Whitehouse	Trial Coordinator
~~	~~~	Mrs Julie Fletcher	Trial Coordinator
UK	Birmingham: Good Hope Hosp	Dr Daniel Ford	Site PI
~~	~~~	Dr John Glaholm	Clinical/Surgical
~~	~~~	Miss Rachael O’Beney	Point of contact (1st)
~~	~~~	Mr Daniel Lenton	Point of contact (1st)
~~	~~~	Mrs Helen Thomas	Research Nurse
~~	~~~	Ms Helen Taylor	Research Nurse
~~	~~~	Mr James Whitehouse	Trial Coordinator
~~	~~~	Ms Sundip Sohanpal	Data Manager
UK	Birmingham: Queen Elizabeth Hosp (Birmingham)	Dr Anjali Zarkar	Site PI
~~	~~~	Dr Ahmed El-Modir	Clinical/Surgical
~~	~~~	Dr Daniel Ford	Clinical/Surgical
~~	~~~	Dr John Glaholm	Clinical/Surgical
~~	~~~	Mr A Doherty	Clinical/Surgical
~~	~~~	Prof Nicholas James	Clinical/Surgical
~~	~~~	Miss Tarandip Samra	Point of contact (1st)

INVESTIGATORS AND COLLABORATORS: SITE STAFF

Staff on site delegation logs

Country	Site	Names	Role
~~	~~~	Mrs Liliam Ross	Point of contact (1st)
~~	~~~	Ms Rachel Lokes	Point of contact (1st)
~~	~~~	Miss Josephine Marange	Research Nurse
~~	~~~	Miss Rebekah Stephens	Research Nurse
~~	~~~	Mr Andrew Morrison	Trial Coordinator
~~	~~~	Mr Phillip Watson-Jones	Trial Coordinator
~~	~~~	Miss Melanie Gunn	Data Manager
~~	~~~	Miss Zhane Peterkin	Data Manager
~~	~~~	Mr George Preece	Data Manager
~~	~~~	Mrs Aliyah Mannan	Data Manager
~~	~~~	Mr Zayn Qureshi	MDT coordinator
~~	~~~	Mrs Elizabeth Southgate	Radiographer
~~	~~~	Mrs Maryanne Okubanjo	Radiographer
~~	~~~	Mrs Samantha Chetiyawardana	Radiographer
~~	~~~	Ms Darlletta Olaniyi-Oduntan	Radiographer
~~	~~~	Ms Liz Kedge	Radiographer
~~	~~~	Dr Robert Stevenson	Other
UK	Blackburn: Burnley General Hosp	Dr Omi Parikh	Site PI
~~	~~~	Dr Catherine Thompson	Clinical/Surgical
~~	~~~	Dr Danya Abdulwahid	Clinical/Surgical
~~	~~~	Dr Imran Haidar	Clinical/Surgical
~~	~~~	Dr Jennifer King	Clinical/Surgical
~~	~~~	Dr Marcus Wise	Clinical/Surgical
~~	~~~	Dr Natalie Charnley	Clinical/Surgical
~~	~~~	Dr Parth Desai	Clinical/Surgical
~~	~~~	Mrs Jacqueline Thomas	Point of contact (1st)
~~	~~~	Ms Jeanette Hargreaves	Point of contact (1st)
~~	~~~	Mrs Janet Ryan-Smith	Point of contact (2nd)
~~	~~~	Mrs Angela Hugill	Research Nurse
~~	~~~	Mrs Helen Frankland	Research Nurse
~~	~~~	Mrs Lynsey Waring	Research Nurse
~~	~~~	Ms Diane Forrest	Research Nurse
~~	~~~	Ms Jan Flaherty	Research Nurse
~~	~~~	Ms Karen Beard	Research Nurse

INVESTIGATORS AND COLLABORATORS: SITE STAFF
Staff on site delegation logs

Country	Site	Names	Role
~~	~~~	Ms Karen Jewers	Research Nurse
~~	~~~	Ms Karen Riley	Research Nurse
~~	~~~	Ms Sarah Ainsworth	Research Nurse
~~	~~~	Ms Sarah Keith	Research Nurse
~~	~~~	Mrs Sue Ashworth	Trial Coordinator
~~	~~~	Miss Bethany Fielding	Other
~~	~~~	Mr Stephen Kilroy	Other
~~	~~~	Ms Joanne Henry	Other
~~	~~~	Ms Vivienne Tickle	Other
UK	Blackburn: Royal Blackburn Hosp	Dr Omi Parikh	Site PI
~~	~~~	Dr Catherine Thompson	Clinical/Surgical
~~	~~~	Dr Danya Abdulwahid	Clinical/Surgical
~~	~~~	Dr Imran Haidar	Clinical/Surgical
~~	~~~	Dr Jennifer King	Clinical/Surgical
~~	~~~	Dr Marcus Wise	Clinical/Surgical
~~	~~~	Dr Parth Desai	Clinical/Surgical
~~	~~~	Ms Jeanette Hargreaves	Point of contact (1st)
~~	~~~	Mrs Janet Ryan-Smith	Point of contact (2nd)
~~	~~~	Mrs Lynsey Waring	Point of contact (2nd)
~~	~~~	Mrs Angela Hugill	Research Nurse
~~	~~~	Mrs Helen Frankland	Research Nurse
~~	~~~	Ms Diane Forrest	Research Nurse
~~	~~~	Ms Jan Flaherty	Research Nurse
~~	~~~	Ms Karen Beard	Research Nurse
~~	~~~	Ms Karen Jewers	Research Nurse
~~	~~~	Ms Karen Riley	Research Nurse
~~	~~~	Ms Louise Dawson	Research Nurse
~~	~~~	Ms Sarah Ainsworth	Research Nurse
~~	~~~	Ms Sarah Keith	Research Nurse
~~	~~~	Mrs Julie Ditchfield	Trial Coordinator
~~	~~~	Mrs Sue Ashworth	Trial Coordinator
~~	~~~	Miss Bethany Fielding	Other
~~	~~~	Mr Stephen Kilroy	Other
~~	~~~	Ms Joanne Henry	Other

INVESTIGATORS AND COLLABORATORS: SITE STAFF

Staff on site delegation logs

Country	Site	Names	Role
~~	~~~	Ms Vivienne Tickle	Other
UK	Bodelwyddan: Glan Clwyd Hosp	Dr Nikhil Oommen	Site PI
~~	~~~	Dr Aileen Flavin	Clinical/Surgical
~~	~~~	Dr Amir Al-Samarraie	Clinical/Surgical
~~	~~~	Dr Zulfiqer Ali	Clinical/Surgical
~~	~~~	Mr H Toussi	Clinical/Surgical
~~	~~~	Mr Vaiku Srinivasan	Clinical/Surgical
~~	~~~	Miss Rachel Manley	Point of contact (1st)
~~	~~~	Mrs Jane Heron	Point of contact (1st)
~~	~~~	Mrs Llinos Davies	Point of contact (1st)
~~	~~~	Ms Heather Thomas	Point of contact (1st)
~~	~~~	Mrs Charley-Anne Rutter	Research Nurse
~~	~~~	Mrs Joanne Lewis	Research Nurse
~~	~~~	Ms Jane Stockport	Research Nurse
~~	~~~	Sister Hayley Tapping	Research Nurse
~~	~~~	Miss Faye Hughes	Trial Coordinator
~~	~~~	Mrs Kim Jackson	Trial Coordinator
~~	~~~	Ms Annette Bolger	Data Manager
~~	~~~	Ms Cathryn Wood	Radiographer
~~	~~~	Dr Nick Smith	Other
UK	Boston: Pilgrim Hosp	Dr Thiagarajan Sreenivasan	Site PI
~~	~~~	Dr Miguel Panades	Clinical/Surgical
~~	~~~	Mr Simon Archer	Point of contact (1st)
~~	~~~	Mrs Kinga Szymiczek	Point of contact (1st)
~~	~~~	Ms Amy Kirkby	Point of contact (1st)
~~	~~~	Ms Joanne Fletcher	Point of contact (1st)
~~	~~~	Ms Tara Lawrence nee Palmer	Point of contact (1st)
~~	~~~	Miss Karen Metcalf	Research Nurse
~~	~~~	Mrs Isobel Thomas	Research Nurse
~~	~~~	Mrs Victoria Knight (n. Sherburn)	Research Nurse
~~	~~~	Ms Anita Young	Research Nurse
~~	~~~	Ms Helen Ginnelly	Research Nurse
~~	~~~	Mrs Beverley Mashegede	Data Manager
UK	Bournemouth: Royal Bournemouth Hosp	Dr Sue Brock	Site PI

INVESTIGATORS AND COLLABORATORS: SITE STAFF
Staff on site delegation logs

Country	Site	Names	Role
~~	~~~	Dr Joseph Davies	Clinical/Surgical
~~	~~~	Ms Sarah Savage	Point of contact (1st)
~~	~~~	Mrs Nicky Naraine	Research Nurse
~~	~~~	Ms Eve Broadley	Research Nurse
~~	~~~	Ms Stephanie Jones	Research Nurse
~~	~~~	Miss Laura Purandare	Data Manager
~~	~~~	Miss Taslima Rabbi	Data Manager
~~	~~~	Mr Luke Vamplew	Data Manager
~~	~~~	Ms Kate Preece	Data Manager
~~	~~~	Ms Ruby McCully	Administrator
UK	Bradford: Bradford Royal Inf	Dr Sree Rodda	Site PI
~~	~~~	Dr Ann Henry	Clinical/Surgical
~~	~~~	Dr Lisa Owen	Clinical/Surgical
~~	~~~	Dr Louise Karsera	Clinical/Surgical
~~	~~~	Dr Mark Teo	Clinical/Surgical
~~	~~~	Ms Chandran Nallathambi	Clinical/Surgical
~~	~~~	Ms Shefali Parikh	Clinical/Surgical
~~	~~~	Miss Jannika Lazarte	Point of contact (1st)
~~	~~~	Mrs Jane Sewell	Point of contact (1st)
~~	~~~	Miss Lucille Kenyon	Research Nurse
~~	~~~	Mrs Hayley Inman	Research Nurse
~~	~~~	Mrs Helen Robertshaw	Research Nurse
~~	~~~	Ms Anne Marie Kay	Research Nurse
~~	~~~	Ms Linda Bamford	Research Nurse
~~	~~~	Miss Amy Pendrill	Data Manager
~~	~~~	Mr Richard Benton	Data Manager
~~	~~~	Miss Eleanor Waldron	Administrator
~~	~~~	Mrs Dawn McNulty	Administrator
~~	~~~	Ms Eleanor Moore	Administrator
~~	~~~	Ms Lauren Cotterill	MDT coordinator
~~	~~~	Ms Deirdre Naylor	Pharmacist
~~	~~~	Ms Joanne Price	Pharmacist
UK	Brighton: Royal Sussex County Hosp	Dr Angus Robinson	Site PI
~~	~~~	Dr Andrew Webb	Clinical/Surgical

INVESTIGATORS AND COLLABORATORS: SITE STAFF

Staff on site delegation logs

Country	Site	Names	Role
~~	~~~	Dr Ashok Nikapota	Clinical/Surgical
~~	~~~	Dr David Bloomfield	Clinical/Surgical
~~	~~~	Dr Duncan Gilbert	Clinical/Surgical
~~	~~~	Dr Fiona McKinna	Clinical/Surgical
~~	~~~	Dr Marie Wilkins	Clinical/Surgical
~~	~~~	Miss Catherine Hunter	Point of contact (1st)
~~	~~~	Mr Paul Frattaroli	Point of contact (1st)
~~	~~~	Ms Jessica Dlodlo	Point of contact (1st)
~~	~~~	Ms Joanne Brown	Point of contact (1st)
~~	~~~	Mr Andrew Hart	Research Nurse
~~	~~~	Ms Madalena Leitao	Research Nurse
~~	~~~	Miss Jean Tremlett	Trial Coordinator
~~	~~~	Ms Bobbie Young	Trial Coordinator
~~	~~~	Ms Jane Peterson	Trial Coordinator
~~	~~~	Ms Tracy Blythe	Trial Coordinator
~~	~~~	Miss Samantha Hodges	Data Manager
~~	~~~	Ms Carla Chambers	Data Manager
~~	~~~	Ms Ranee Lactao	Data Manager
~~	~~~	Mr Simon Hooper	Radiographer
~~	~~~	Ms Philippa Carr	Radiographer
UK	Bristol: Bristol Haematology & Oncology Centre	Dr Amit Bahl	Site PI
~~	~~~	Dr Amarnath Challipalli	Clinical/Surgical
~~	~~~	Dr Mark Beresford	Clinical/Surgical
~~	~~~	Dr Paula Wilson	Clinical/Surgical
~~	~~~	Dr Serena Hilman	Clinical/Surgical
~~	~~~	Dr Susan Masson	Clinical/Surgical
~~	~~~	Mr David Gillatt	Clinical/Surgical
~~	~~~	Mr Marc Coe	Point of contact (1st)
~~	~~~	Mr Martin Woods	Point of contact (1st)
~~	~~~	Ms Vivienne Lee	Point of contact (1st)
~~	~~~	Mr Matt Baxter	Point of contact (2nd)
~~	~~~	Miss Mary Kisanga	Research Nurse
~~	~~~	Mr Peter Robertson	Research Nurse
~~	~~~	Mrs Dorothy Griffiths	Research Nurse

INVESTIGATORS AND COLLABORATORS: SITE STAFF

Staff on site delegation logs

Country	Site	Names	Role
~~	~~~	Miss Amelia Lowe	Trial Coordinator
~~	~~~	Ms Helen Saldanha	Trial Coordinator
~~	~~~	Ms Rhiannon Macefield	Trial Coordinator
~~	~~~	Ms Ruth Pegler	Trial Coordinator
~~	~~~	Ms Verity Henson (nee Savidge)	Trial Coordinator
~~	~~~	Mr Robert Hollister	Data Manager
~~	~~~	Ms Laura Sims	Data Manager
~~	~~~	Mrs Lucie Wheeler	Administrator
~~	~~~	Miss Mary Simmonds	Radiographer
~~	~~~	Mrs Sarah Zelley	Radiographer
~~	~~~	Mrs Sue Cowley	Radiographer
~~	~~~	Mrs Sue Yarrow	Radiographer
~~	~~~	Ms Dawn Bowers	Radiographer
UK	Bristol: Southmead Hosp	Mr Raj Persad	Site PI
~~	~~~	Dr Amarnath Challipalli	Clinical/Surgical
~~	~~~	Dr Amit Bahl	Clinical/Surgical
~~	~~~	Dr Chris Herbert	Clinical/Surgical
~~	~~~	Dr Mark Beresford	Clinical/Surgical
~~	~~~	Dr Paula Wilson	Clinical/Surgical
~~	~~~	Mr Anthony Koupparis	Clinical/Surgical
~~	~~~	Mr David Gillatt	Clinical/Surgical
~~	~~~	Mr Edward Rowe	Clinical/Surgical
~~	~~~	Mrs Samantha Clarke	Point of contact (1st)
~~	~~~	Ms Emily Perry	Point of contact (1st)
~~	~~~	Ms Lyndsey Johnson	Point of contact (1st)
~~	~~~	Ms Marta Cobos-Arrivabene	Point of contact (1st)
~~	~~~	Ms Rebecca Cousins	Point of contact (1st)
~~	~~~	Ms Victoria Garner	Point of contact (1st)
~~	~~~	Ms Carol Brain	Point of contact (2nd)
~~	~~~	Ms Constance Shiridzinomwa	Point of contact (2nd)
~~	~~~	Ms Kathryn Jones	Point of contact (2nd)
~~	~~~	Mrs Sarah Kirkby	Research Nurse
~~	~~~	Mrs Suriya Kirkpatrick	Research Nurse
~~	~~~	Ms Charlotte Phipps	Research Nurse

INVESTIGATORS AND COLLABORATORS: SITE STAFF
Staff on site delegation logs

Country	Site	Names	Role
~~	~~~	Ms Helen Corderoy	Research Nurse
~~	~~~	Ms Carolyn Smith	Trial Coordinator
~~	~~~	Ms Kate Alderton	Trial Coordinator
~~	~~~	Mrs Kerry Richmond-Russell	Data Manager
~~	~~~	Ms Ann Treasure	Administrator
~~	~~~	Miss Clare Wyatt	MDT coordinator
~~	~~~	Ms Courtney Manning	MDT coordinator
~~	~~~	Dr Jon Oxley	Other
~~	~~~	Mr Luke Shelton	Other
~~	~~~	Mr Mohamed Abd Alazeez	Other
~~	~~~	Ms Gail Kemp	Other
~~	~~~	Ms Helen Chilcott	Other
UK	Cambridge: Addenbrooke's Hosp	Dr Yvonne Rimmer	Site PI
~~	~~~	Dr Cathryn Woodward	Clinical/Surgical
~~	~~~	Dr Danish Mazhar	Clinical/Surgical
~~	~~~	Dr Helen Patterson	Clinical/Surgical
~~	~~~	Dr Luke Hughes-Davies	Clinical/Surgical
~~	~~~	Dr Ramya Ramanujachar	Clinical/Surgical
~~	~~~	Dr Richard Benson	Clinical/Surgical
~~	~~~	Dr Robert Thomas	Clinical/Surgical
~~	~~~	Dr Sanjay Raj	Clinical/Surgical
~~	~~~	Dr Simon Russell	Clinical/Surgical
~~	~~~	Ms Juliette Harnwell	Point of contact (1st)
~~	~~~	Mr Matthew Stone	Point of contact (2nd)
~~	~~~	Miss Vicky Joslin	Trial Coordinator
~~	~~~	Mrs Vanessa Goss	Trial Coordinator
~~	~~~	Ms Angelique Laubscher	Administrator
~~	~~~	Ms Ellie Couch	Administrator
~~	~~~	Ms Katherine Beesley	Administrator
~~	~~~	Mrs Sue Foxwell	MDT coordinator
~~	~~~	Miss Nicola Robinson	Radiographer
~~	~~~	Mrs Jo Treeby	Radiographer
~~	~~~	Ms Kathryn Vickery	Radiographer
UK	Canterbury: Kent & Canterbury Hosp	Dr Rakesh Raman	Site PI

INVESTIGATORS AND COLLABORATORS: SITE STAFF

Staff on site delegation logs

Country	Site	Names	Role
~~	~~~	Dr Albert Edwards	Clinical/Surgical
~~	~~~	Dr Alice Rendall	Clinical/Surgical
~~	~~~	Dr Carys Thomas	Clinical/Surgical
~~	~~~	Dr Christos Mikropoulos	Clinical/Surgical
~~	~~~	Dr Clary Evans	Clinical/Surgical
~~	~~~	Dr Gemma Hegarty	Clinical/Surgical
~~	~~~	Dr Ilyas Ahmed	Clinical/Surgical
~~	~~~	Dr Ioannis Trigonis	Clinical/Surgical
~~	~~~	Dr Jessica Gough	Clinical/Surgical
~~	~~~	Dr Jessica Little	Clinical/Surgical
~~	~~~	Dr Joao Galante	Clinical/Surgical
~~	~~~	Dr Kannon Nathan	Clinical/Surgical
~~	~~~	Dr Natasha Mithal	Clinical/Surgical
~~	~~~	Dr Patryk Brulinski	Clinical/Surgical
~~	~~~	Dr Rohit Malde	Clinical/Surgical
~~	~~~	Dr Van Sim	Clinical/Surgical
~~	~~~	Mr Arafat Mirza	Clinical/Surgical
~~	~~~	Mrs Laura Kehoe	Point of contact (1st)
~~	~~~	Mrs Laura Mould	Point of contact (1st)
~~	~~~	Mrs Louise Gladwell	Point of contact (1st)
~~	~~~	Mrs Julie Buckley	Research Nurse
~~	~~~	Ms Carolyn Hargreaves	Research Nurse
~~	~~~	Ms Elizabeth Williamson	Research Nurse
~~	~~~	Ms Joanne Williams	Research Nurse
~~	~~~	Ms Julie-Ann Davies	Research Nurse
~~	~~~	Ms Michelle Swann	Research Nurse
~~	~~~	Ms Pauline Wood	Research Nurse
~~	~~~	Ms Rachel Ryan	Research Nurse
~~	~~~	Ms Susan Drakeley	Research Nurse
~~	~~~	Sister Denise Crawford	Research Nurse
~~	~~~	Mrs Rachel Larkins	Trial Coordinator
~~	~~~	Ms Bonny Appleby	Trial Coordinator
~~	~~~	Ms Hilary Zurakovsky	Trial Coordinator
~~	~~~	Ms Karen Robinson	Trial Coordinator

INVESTIGATORS AND COLLABORATORS: SITE STAFF
Staff on site delegation logs

Country	Site	Names	Role
~~	~~~	Ms Marian Wood	Trial Coordinator
~~	~~~	Ms Sharon Middleton	Trial Coordinator
~~	~~~	Mrs Laura Mould	Administrator
~~	~~~	Ms Lorraine Tasker	MDT coordinator
~~	~~~	Miss Katy Taylor	Radiographer
~~	~~~	Miss Natalie Catt	Radiographer
~~	~~~	Ms Helen Coppins	Radiographer
~~	~~~	Ms Karen Walker	Radiographer
~~	~~~	Ms Margaret Lipsham	Radiographer
UK	Cardiff: University Hosp of Wales	Mr Krishna Narahari	Site PI
~~	~~~	Dr John Staffurth	Clinical/Surgical
~~	~~~	Mr Owen Hughes	Clinical/Surgical
~~	~~~	Ms Hin Fan Chan	Clinical/Surgical
~~	~~~	Prof Howard Kynaston	Clinical/Surgical
~~	~~~	Prof Malcolm Mason	Clinical/Surgical
~~	~~~	Miss Elizabeth Bois (nee Harris)	Point of contact (1st)
~~	~~~	Ms Colette Clements	Point of contact (1st)
~~	~~~	Mr Kevin Pearse	Research Nurse
~~	~~~	Mrs Clare Jones	Research Nurse
~~	~~~	Mrs Loveness Chikopela	Research Nurse
~~	~~~	Mrs Samantha Holliday	Research Nurse
~~	~~~	Mrs Helen Clark	Data Manager
UK	Chelmsford: Broomfield Hosp	Dr Abdel Hamid	Site PI
~~	~~~	Dr Kiran Kancherla	Clinical/Surgical
~~	~~~	Dr Priscilla Leone	Clinical/Surgical
~~	~~~	Mr Bryan Singizi	Point of contact (1st)
~~	~~~	Ms Sian Gibson	Point of contact (1st)
~~	~~~	Ms Lauren Perkins	Point of contact (2nd)
~~	~~~	Ms Mandy Austin	Point of contact (2nd)
~~	~~~	Ms Elizabeth Dawson	Research Nurse
~~	~~~	Sister Tracey Camburn	Research Nurse
~~	~~~	Ms Valerie Ramsay	Trial Coordinator
~~	~~~	Mr Christian Barnett	Data Manager
UK	Cheltenham: Cheltenham General Hosp	Dr Jo Bowen	Site PI

INVESTIGATORS AND COLLABORATORS: SITE STAFF
Staff on site delegation logs

Country	Site	Names	Role
~~	~~~	Dr Audrey Cook	Clinical/Surgical
~~	~~~	Dr Chris Shimell	Clinical/Surgical
~~	~~~	Dr Colin Binks	Clinical/Surgical
~~	~~~	Dr Peter Jenkins	Clinical/Surgical
~~	~~~	Dr Roger Owen	Clinical/Surgical
~~	~~~	Dr Sai Jonnada	Clinical/Surgical
~~	~~~	Miss Sarah Beazer	Point of contact (1st)
~~	~~~	Mr Matthew Tan	Point of contact (1st)
~~	~~~	Mrs Julie Allen	Point of contact (1st)
~~	~~~	Mrs Kate Trigg-Hogarth	Point of contact (2nd)
~~	~~~	Miss Amy Skelton	Research Nurse
~~	~~~	Mrs Chris Ford	Research Nurse
~~	~~~	Mrs Elaine Pratten	Research Nurse
~~	~~~	Mrs Sue Wronski	Research Nurse
~~	~~~	Mrs Susan Anderson	Research Nurse
~~	~~~	Mrs Barbara Broomfield	Trial Coordinator
~~	~~~	Mrs Rachel Sayers	Trial Coordinator
~~	~~~	Ms Janet Forkes	Trial Coordinator
~~	~~~	Ms Rehana Bakawala	Trial Coordinator
~~	~~~	Mr Nigel Johnson	Data Manager
~~	~~~	Mrs Julia Hall	Data Manager
~~	~~~	Mrs Lin Crossley	Data Manager
~~	~~~	Ms Jennifer Healey-Mariano	Research Asst
~~	~~~	Miss Eleanor Andrews (nee Moore)	Administrator
UK	Chester: Countess of Chester Hosp	Dr Azman Ibrahim	Site PI
~~	~~~	Miss Elizabeth Gallimore	Point of contact (1st)
~~	~~~	Mrs Sue Green	Point of contact (1st)
~~	~~~	Ms Carys Jones	Point of contact (1st)
~~	~~~	Ms Jude Prince	Point of contact (1st)
~~	~~~	Ms Mary Aldous	Research Nurse
~~	~~~	Ms Denise Archer	Data Manager
~~	~~~	Ms Helen Eccleson	Data Manager
UK	Chesterfield: Chesterfield Royal Hosp	Dr Omar Din	Site PI
~~	~~~	Dr Peter Kirkbride	Clinical/Surgical

INVESTIGATORS AND COLLABORATORS: SITE STAFF

Staff on site delegation logs

Country	Site	Names	Role
~~	~~~	Ms Lucy Smith	Clinical/Surgical
~~	~~~	Ms Lesley Stevenson	Point of contact (1st)
~~	~~~	Ms Vittoria Sorice	Point of contact (1st)
~~	~~~	Mrs Nicky Ford	Point of contact (2nd)
~~	~~~	Mrs Melanie Chrystal	Research Nurse
~~	~~~	Ms Julie Toms	Research Nurse
~~	~~~	Sister Kim Wood	Research Nurse
~~	~~~	Ms Alison Redfearn	Trial Coordinator
~~	~~~	Miss Alexandra Firth	Data Manager
~~	~~~	Mr John Martindale	Data Manager
~~	~~~	Mrs Janine Smedley (nee McCabe)	Data Manager
UK	Colchester: Essex County Hosp	Dr Dakshinamoorthy Muthu Kumar	Site PI
~~	~~~	Dr Bruce Sizer	Clinical/Surgical
~~	~~~	Dr Devy Basu	Clinical/Surgical
~~	~~~	Dr Muthar Kumar	Clinical/Surgical
~~	~~~	Dr Priscilla Leone	Clinical/Surgical
~~	~~~	Mr Louies Mabelin	Point of contact (1st)
~~	~~~	Mrs Liz Hunting	Point of contact (1st)
~~	~~~	Ms Celine Driscoll	Point of contact (1st)
~~	~~~	Ms Lorna Dewar	Trial Coordinator
~~	~~~	Mrs Lucy Thorogood	Data Manager
~~	~~~	Ms Nyssa Barke	Administrator
UK	Cornwall: Royal Cornwall Hosp	Dr Alastair H Thomson	Site PI
~~	~~~	Dr Aaron Gould	Clinical/Surgical
~~	~~~	Dr Duncan Wheatley	Clinical/Surgical
~~	~~~	Miss Elizabeth Firth	Point of contact (1st)
~~	~~~	Mr Christopher Hocking	Point of contact (1st)
~~	~~~	Ms Anita Steele	Point of contact (1st)
~~	~~~	Ms Corrine Penhaligon	Point of contact (1st)
~~	~~~	Miss Alice Topps	Point of contact (2nd)
~~	~~~	Ms Catherine Pentecost	Point of contact (2nd)
~~	~~~	Ms Jane Broom	Point of contact (2nd)
~~	~~~	Ms Sarah Askew	Point of contact (2nd)
~~	~~~	Mrs Thea Barlow	Research Nurse

INVESTIGATORS AND COLLABORATORS: SITE STAFF

Staff on site delegation logs

Country	Site	Names	Role
~~	~~~	Mrs Thea Barlow	Research Nurse
~~	~~~	Ms Emma Duley	Research Nurse
~~	~~~	Ms Emma Kent	Research Nurse
~~	~~~	Dr Darren Beech	Trial Coordinator
~~	~~~	Miss Melissa Poultney	Data Manager
~~	~~~	Mr Luke Townley	Data Manager
~~	~~~	Mr William Pynsent	Data Manager
~~	~~~	Ms Kerena Partridge	Data Manager
~~	~~~	Mr John Madine	Research Asst
~~	~~~	Miss Louise Johns	Administrator
~~	~~~	Mrs Kay Pollard	Administrator
~~	~~~	Ms Johanna Skewes	Pharmacist
UK	Coventry: University Hosp Coventry & Warwickshire	Dr Jane Worlding	Site PI
~~	~~~	Dr Andrew Chan	Clinical/Surgical
~~	~~~	Dr Andrew Stockdale	Clinical/Surgical
~~	~~~	Dr Caroline Humber	Clinical/Surgical
~~	~~~	Dr Medy Tsalic	Clinical/Surgical
~~	~~~	Dr Yakhub Khan	Clinical/Surgical
~~	~~~	Manreet Thind	Point of contact (1st)
~~	~~~	Mr Albert Mislang	Point of contact (1st)
~~	~~~	Ms Kerry Geraghty	Point of contact (1st)
~~	~~~	Manju Sunny	Point of contact (2nd)
~~	~~~	Mrs Laura Stanley	Point of contact (2nd)
~~	~~~	Mrs Elaine Simmons	Research Nurse
~~	~~~	Mrs Lesley Hayward	Research Nurse
~~	~~~	Mrs Rosaleen Laverick	Research Nurse
~~	~~~	Ms Fiona Tranter	Research Nurse
~~	~~~	Ms Kay Sanders	Research Nurse
~~	~~~	Ms Su Ngwenya	Research Nurse
~~	~~~	Sister Judith Lake	Research Nurse
~~	~~~	Mr Pritpal Panesar	Trial Coordinator
~~	~~~	Ms Maggie Brown	Trial Coordinator
~~	~~~	Miss Emily Steventon	Data Manager
~~	~~~	Mrs Sue Elwell	Data Manager

INVESTIGATORS AND COLLABORATORS: SITE STAFF

Staff on site delegation logs

Country	Site	Names	Role
~~	~~~	Mrs Theresa Griffiths	Data Manager
~~	~~~	Miss Melanie Sinfield	Administrator
~~	~~~	Ms Gemma Mansell	Administrator
~~	~~~	Ms Marie Connor	MDT coordinator
UK	Crewe: Leighton Hosp	Dr Anna Tran	Site PI
~~	~~~	Dr James Wylie	Clinical/Surgical
~~	~~~	Dr John Logue	Clinical/Surgical
~~	~~~	Mr P Irwin	Clinical/Surgical
~~	~~~	Mr P Javle	Clinical/Surgical
~~	~~~	Miss Adele Hough	Point of contact (1st)
~~	~~~	Mrs Caroline Walker	Point of contact (1st)
~~	~~~	Ms Carolyn Mansfield	Point of contact (1st)
~~	~~~	Ms Joanne Hughes	Point of contact (1st)
~~	~~~	Ms Lesley Sumner	Point of contact (1st)
~~	~~~	Ms Rachel Smith	Point of contact (1st)
~~	~~~	Miss Annabel Tomlinson	Point of contact (2nd)
~~	~~~	Mrs Chris Hough	Research Nurse
~~	~~~	Ms Vanessa Adamson	Research Nurse
~~	~~~	Mrs Carole Bennion	Administrator
~~	~~~	Ms Jenny Butler-Barnes	Other
UK	Derby: Royal Derby Hosp	Dr Prantik Das	Site PI
~~	~~~	Dr Dakshinamoorthy Muthukumar	Clinical/Surgical
~~	~~~	Dr Prabir Chakraborti	Clinical/Surgical
~~	~~~	Mr Pugazhenthi Pattu	Clinical/Surgical
~~	~~~	Ms Dawn Ennis	Point of contact (1st)
~~	~~~	Ms Sue Marriott	Research Nurse
~~	~~~	Mr Colin Ward	Pharmacist
UK	Doncaster: Doncaster Royal Inf	Dr Carmel Pezaro	Site PI
~~	~~~	Dr Catherine Ferguson	Clinical/Surgical
~~	~~~	Dr Mymoona Alzouebi	Clinical/Surgical
~~	~~~	Ms Lucy Smith	Clinical/Surgical
~~	~~~	Mrs Nicola Wilkinson	Point of contact (1st)
~~	~~~	Ms Georgia Hooton	Point of contact (2nd)
~~	~~~	Miss Jennifer Taylor	Research Nurse

INVESTIGATORS AND COLLABORATORS: SITE STAFF
Staff on site delegation logs

Country	Site	Names	Role
~~	~~~	Mrs Amy Neal	Research Nurse
~~	~~~	Mrs Nicole Jeffcutt	Research Nurse
~~	~~~	Ms Barbara Burlace	Research Nurse
~~	~~~	Sister Kim Wood	Research Nurse
~~	~~~	Miss Alexandra Firth	Data Manager
~~	~~~	Mr John Martindale	Data Manager
~~	~~~	Mrs Janine Smedley (nee McCabe)	Data Manager
~~	~~~	Dr Virgil Sivoglo	Other
UK	Dorchester: Dorset County Hosp	Dr Benjamin Masters	Site PI
~~	~~~	Mr Naveed Afzal	Site PI
~~	~~~	Dr Perric Crellin	Clinical/Surgical
~~	~~~	Mr Andrew Cornaby	Clinical/Surgical
~~	~~~	Mr Stephen Andrews	Clinical/Surgical
~~	~~~	Mrs Josie Goodsell	Point of contact (1st)
~~	~~~	Ms Kate Taylor	Point of contact (1st)
~~	~~~	Mr Piet Bakker	Research Nurse
~~	~~~	Mr Simon Sharpe	Research Nurse
~~	~~~	Mrs Jackie Gibbins	Research Nurse
~~	~~~	Mrs Sally Love	Research Nurse
~~	~~~	Ms Beverley Anderson	Research Nurse
~~	~~~	Ms Sally Breakspear	Research Nurse
~~	~~~	Ms Sarah Horton	Research Nurse
~~	~~~	Ms Stephanie Jones	Research Nurse
~~	~~~	Ms Tracy Glen	Research Nurse
~~	~~~	Mr Andrew Gibbins	Data Manager
~~	~~~	Mr Andrew Rees	Data Manager
~~	~~~	Ms Suzy Wignall	Data Manager
UK	Dudley: Russells Hall Hosp	Dr Pek Keng-Koh	Site PI
~~	~~~	Dr Prakash Ramachandra	Clinical/Surgical
~~	~~~	Lesley Jones	Point of contact (1st)
~~	~~~	Sister Kath Harrow	Point of contact (1st)
~~	~~~	Ms Karen McGarry	Point of contact (2nd)
~~	~~~	Mrs Lucy Smith	Research Nurse
~~	~~~	Ms Karen Kanyi	Research Nurse

INVESTIGATORS AND COLLABORATORS: SITE STAFF

Staff on site delegation logs

Country	Site	Names	Role
~~	~~~	Ms Angela Watts	Other
UK	Eastbourne: Eastbourne District General Hosp	Dr Caroline Manetta	Site PI
~~	~~~	Dr Duncan Gilbert	Clinical/Surgical
~~	~~~	Dr Fiona McKinna	Clinical/Surgical
~~	~~~	Mrs Jo-Anne Taylor	Point of contact (1st)
~~	~~~	Ms Shelley Baumber	Point of contact (2nd)
~~	~~~	Mrs Kay Jones-Skipper	Research Nurse
~~	~~~	Ms Lauren McCrisken	Research Nurse
~~	~~~	Ms Amanda Williams	Trial Coordinator
~~	~~~	Ms Joanna Howard	Data Manager
UK	Edinburgh: Western General Hosp	Dr Duncan McLaren	Site PI
~~	~~~	Mr Param Mariappan	Clinical/Surgical
~~	~~~	Ms Susan Forman	Point of contact (1st)
~~	~~~	Ms Catherine Woods	Point of contact (2nd)
~~	~~~	Mrs Barbara Mayne	Research Nurse
~~	~~~	Ms Lisa Egan	Research Nurse
~~	~~~	Ms Kathleen Fiddes	Trial Coordinator
~~	~~~	Mr Brian Rogers	Data Manager
~~	~~~	Mr David Jeffrey	Data Manager
~~	~~~	Ms Alison Clark	Data Manager
~~	~~~	Ms Fiona Gardiner	Data Manager
~~	~~~	Ms Maria Clarke	Data Manager
~~	~~~	Dr Douglas Young	Other
UK	Exeter: Royal Devon & Exeter Hosp	Dr Rajaguru Srinivasan	Site PI
~~	~~~	Dr Victoria Ford	Site PI
~~	~~~	Dr Denise Sheehan	Clinical/Surgical
~~	~~~	Dr John McGrath	Clinical/Surgical
~~	~~~	Mr M Crundwell	Clinical/Surgical
~~	~~~	Mr Mark Stott	Clinical/Surgical
~~	~~~	Mrs Claire Webb	Point of contact (1st)
~~	~~~	Mrs Ingrid Seath	Point of contact (1st)
~~	~~~	Ms Ellen Matkins	Point of contact (1st)
~~	~~~	Ms Jane Piper	Point of contact (1st)
~~	~~~	Ms Kizzy Baines	Point of contact (1st)

INVESTIGATORS AND COLLABORATORS: SITE STAFF

Staff on site delegation logs

Country	Site	Names	Role
~~	~~~	Ms Alison Roantree	Research Nurse
~~	~~~	Ms Elizabeth Davey	Research Nurse
~~	~~~	Ms Shannon McGinley	Trial Coordinator
~~	~~~	Mr John Anderson	Data Manager
~~	~~~	Miss Theresa Lawless	Administrator
~~	~~~	Ms Katie Timmings	Radiographer
UK	Glasgow: Beatson West of Scotland Cancer Centre	Dr Azmat Sadozye	Site PI
~~	~~~	Dr Abdulla Al-hasso	Clinical/Surgical
~~	~~~	Dr David Dodds	Clinical/Surgical
~~	~~~	Dr Jan Wallace	Clinical/Surgical
~~	~~~	Dr Martin Russell	Clinical/Surgical
~~	~~~	Dr Norma Sidek	Clinical/Surgical
~~	~~~	Dr Rana Mahmood	Clinical/Surgical
~~	~~~	Dr Rob Jones	Clinical/Surgical
~~	~~~	Mrs Ailsa Griffen	Point of contact (1st)
~~	~~~	Niall Finnegan	Point of contact (1st)
~~	~~~	Ms Chloe Cowan	Research Nurse
~~	~~~	Ms Lorraine Barwell	Research Nurse
~~	~~~	Ms Antonia MacMillan	Trial Coordinator
~~	~~~	Ms Sian Shirley	Trial Coordinator
~~	~~~	Ms Claire Lawless	Data Manager
~~	~~~	Ms Elaine Allan	Administrator
UK	Glasgow: The New Victoria ACH (Ambulatory Care Hosp)	Dr Abdulla Al-hasso	Site PI
~~	~~~	Dr Jawaher Ansari	Clinical/Surgical
~~	~~~	Mr Naeem Akhtar	Clinical/Surgical
~~	~~~	Mrs Emma Moody	Point of contact (1st)
~~	~~~	Ms Louise Humpreys	Point of contact (1st)
~~	~~~	Mrs Karen Bell	Research Nurse
~~	~~~	Ms Claudia Turley	Research Nurse
~~	~~~	Ms Donna McWilliam	Research Nurse
~~	~~~	Ms Rebecca O'Neil	Research Nurse
~~	~~~	Ms Suzannah Peck	Research Nurse
UK	Guildford: Royal Surrey County Hosp	Dr Chee Goh	Site PI
~~	~~~	Dr Aruna Medisetti	Clinical/Surgical

INVESTIGATORS AND COLLABORATORS: SITE STAFF
Staff on site delegation logs

Country	Site	Names	Role
~~	~~~	Dr Jenny Nobes	Clinical/Surgical
~~	~~~	Dr Julian Money-Kyrle	Clinical/Surgical
~~	~~~	Dr Richard Shaffer	Clinical/Surgical
~~	~~~	Dr Robert Laing	Clinical/Surgical
~~	~~~	Dr Sara Khaksar	Clinical/Surgical
~~	~~~	Dr Teresa Guerrero-Urbano	Clinical/Surgical
~~	~~~	Mrs Jules Jones	Point of contact (1st)
~~	~~~	Ms Laura Matthews	Point of contact (1st)
~~	~~~	Ms Raidah Auladin	Point of contact (1st)
~~	~~~	Ms Sarah Stone	Point of contact (1st)
~~	~~~	Mrs Barbara Molony-Oates	Research Nurse
~~	~~~	Mrs Caterina Bissa	Research Nurse
~~	~~~	Mrs Jane Woods	Research Nurse
~~	~~~	Mrs Min Wu	Trial Coordinator
~~	~~~	Ms Daisy May Smale	Trial Coordinator
~~	~~~	Ms Frances Sidi	Administrator
~~	~~~	Miss Weronika Rabsztyn	Radiographer
~~	~~~	Mr Fabio Di Maria	Radiographer
~~	~~~	Mr Joshua Harding	Radiographer
~~	~~~	Mrs Annie Tindall	Radiographer
~~	~~~	Ms Ceri Jamieson	Radiographer
~~	~~~	Ms Marianne Dabbs	Radiographer
~~	~~~	Ms Stephanie Bird	Radiographer
UK	Harlow: Princess Alexandra Hosp (Harlow)	Dr Lucinda Melcher	Site PI
~~	~~~	Ms Amelia Daniel	Point of contact (1st)
~~	~~~	Ms Teresa Light	Point of contact (1st)
~~	~~~	Ms Lily Robinson	Point of contact (2nd)
~~	~~~	Ms Nikki Staines	Point of contact (2nd)
~~	~~~	Ms Amanda Lewis	Research Nurse
~~	~~~	Ms Joanne Kellaway	Research Nurse
~~	~~~	Ms Evelyn Holmes	Pharmacist
UK	Hereford: Hereford County Hosp	Dr Warren Grant	Site PI
~~	~~~	Dr Audrey Cook	Clinical/Surgical
~~	~~~	Mr Josh Follows	Point of contact (1st)

INVESTIGATORS AND COLLABORATORS: SITE STAFF
Staff on site delegation logs

Country	Site	Names	Role
~~	~~~	Mrs Janine Jones (Birch)	Point of contact (1st)
~~	~~~	Mrs Melanie Evans	Point of contact (1st)
~~	~~~	Miss Lisa King	Point of contact (2nd)
~~	~~~	Miss Sophie Cooper	Point of contact (2nd)
~~	~~~	Ms Sophie Myles	Point of contact (2nd)
~~	~~~	Miss Nicola Williamson	Research Nurse
~~	~~~	Miss Serrafina Carini	Research Nurse
~~	~~~	Mrs Susan Anderson	Research Nurse
~~	~~~	Ms Claire Hughes	Research Nurse
~~	~~~	Ms Lily Mercer	Research Nurse
~~	~~~	Ms Janet Forkes	Trial Coordinator
~~	~~~	Mrs Zara Roberts	Data Manager
~~	~~~	Mrs Laura Lees	Administrator
~~	~~~	Mr Andy Hedges	Pharmacist
UK	Herts: Mount Vernon Hosp	Dr Peter Ostler	Site PI
~~	~~~	Prof Peter Hoskin	Site PI
~~	~~~	Dr Jeanette Dickson	Clinical/Surgical
~~	~~~	Dr Nicola Anyamene	Clinical/Surgical
~~	~~~	Dr Robert Hughes	Clinical/Surgical
~~	~~~	Dr Roberto Alonzi	Clinical/Surgical
~~	~~~	Miss Lucy Collins	Point of contact (1st)
~~	~~~	Ms Justina Kailey	Point of contact (1st)
~~	~~~	Ms Justina Kailey	Point of contact (1st)
~~	~~~	Ms Lesley Mitchell	Point of contact (1st)
~~	~~~	Ms Sara Abbassi	Point of contact (2nd)
~~	~~~	Ms Jessica Milner	Research Nurse
~~	~~~	Ms Julia Bici	Research Nurse
~~	~~~	Miss Aamna Rashid	Trial Coordinator
~~	~~~	Ms Paulina Kowalewska	Trial Coordinator
~~	~~~	Ms Suzanne Jenkins	Trial Coordinator
~~	~~~	Miss Stephanie Stapleton	Data Manager
~~	~~~	Ms Sandra Garrido-Perez	Other
UK	Hull: Castle Hill Hosp	Mr Matthew Simms	Site PI
~~	~~~	Dr Faheem Bashir	Clinical/Surgical

INVESTIGATORS AND COLLABORATORS: SITE STAFF
Staff on site delegation logs

Country	Site	Names	Role
~~	~~~	Dr Sanjay Dixit	Clinical/Surgical
~~	~~~	Mrs Julie Rawlings	Point of contact (1st)
~~	~~~	Mrs Diane Clark	Point of contact (2nd)
~~	~~~	Mrs Linzi Bone	Research Nurse
~~	~~~	Ms Sarah Palmer	Research Nurse
~~	~~~	Mr Kristian Plowman	Data Manager
~~	~~~	Mrs Dawn Jones	Data Manager
~~	~~~	Mrs Rhian Horne	Pharmacist
UK	Inverness: Raigmore Hosp	Dr Neil McPhail	Site PI
~~	~~~	Dr Alison Nicholls	Clinical/Surgical
~~	~~~	Dr Aristoula Papakostidi	Clinical/Surgical
~~	~~~	Dr Carol Macgregor	Clinical/Surgical
~~	~~~	Dr David Whillis	Clinical/Surgical
~~	~~~	Dr Kay Kelly	Clinical/Surgical
~~	~~~	Mr Sudhir Borgaonkar	Clinical/Surgical
~~	~~~	Mrs Rachel Mackay	Point of contact (1st)
~~	~~~	Mr Sean Neville	Research Nurse
~~	~~~	Mrs Anglise Addison	Research Nurse
~~	~~~	Mrs Georgina Simpson	Research Nurse
~~	~~~	Mrs Sandra Brown	Research Nurse
~~	~~~	Ms Morag McNally	Research Nurse
~~	~~~	Mrs Alison Macdonald	Data Manager
~~	~~~	Mrs Seonaid Arnott	Data Manager
~~	~~~	Ms Anna Skene	Data Manager
~~	~~~	Ms Glenda Sinclair	Data Manager
~~	~~~	Ms Debbie Lister	MDT coordinator
~~	~~~	Mr Jude Madeleine	Pharmacist
~~	~~~	Ms Audrey Campbell	Pharmacist
~~	~~~	Ms Zoe Urquhart	Pharmacist
~~	~~~	Mrs Margaret Cormack	Radiographer
~~	~~~	Mrs Sheena Telfer	Radiographer
~~	~~~	Mrs Victoria Doughty	Radiographer
~~	~~~	Mr Steve Colligan	Other
UK	Ipswich: Ipswich Hosp	Dr Christopher Scrase	Site PI

INVESTIGATORS AND COLLABORATORS: SITE STAFF
Staff on site delegation logs

Country	Site	Names	Role
~~	~~~	Dr Ramachandran Venkitaraman	Clinical/Surgical
~~	~~~	Mr Paul Ridley	Point of contact (1st)
~~	~~~	Ms Susan Upson	Point of contact (1st)
~~	~~~	Mrs Amanda Ford	Radiographer
~~	~~~	Mrs Charlotte Etheridge	Other
UK	Kidderminster: Kidderminster General Hosp	Dr Lisa Capaldi	Site PI
~~	~~~	Dr Mark Churn	Clinical/Surgical
~~	~~~	Mrs Helen Tranter	Point of contact (1st)
~~	~~~	Mr Jacob Taylor	Point of contact (2nd)
~~	~~~	Mrs Julie Wollaston	Research Nurse
~~	~~~	Ms Helen Knott	Research Nurse
~~	~~~	Ms Sally Stringer (pr. Davis)	Research Nurse
~~	~~~	Ms Linda Higgins	Data Manager
UK	Larbert: Forth Valley Royal Hosp	Dr Norma Sidek	Site PI
~~	~~~	Dr Martin Russell	Clinical/Surgical
~~	~~~	Mr James Tweedle	Clinical/Surgical
~~	~~~	Mr Seamus Teahan	Clinical/Surgical
~~	~~~	Miss Stephanie Brogan (nee Roddie)	Point of contact (1st)
~~	~~~	Mrs Lynn Prentice	Point of contact (1st)
~~	~~~	Mrs Anne Todd	Point of contact (2nd)
~~	~~~	Mrs Lesley Symon	Point of contact (2nd)
~~	~~~	Mrs Sally Young	Research Nurse
~~	~~~	Ms Susan Erskine	MDT coordinator
~~	~~~	Mrs Maureen Hamill	Other
UK	Leeds: St James University Hosp (Leeds)	Dr David Bottomley	Site PI
~~	~~~	Dr Ann Henry	Clinical/Surgical
~~	~~~	Dr Anne Kiltie	Clinical/Surgical
~~	~~~	Dr Carmel Loughrey	Clinical/Surgical
~~	~~~	Dr Catherine Coyle	Clinical/Surgical
~~	~~~	Dr Ian Boon	Clinical/Surgical
~~	~~~	Mr Edmund Breckin	Point of contact (1st)
~~	~~~	Mrs Pam Shuttleworth	Point of contact (1st)
~~	~~~	Ms Beccy Smith	Point of contact (1st)
~~	~~~	Mrs Jude Clarke	Research Nurse

INVESTIGATORS AND COLLABORATORS: SITE STAFF
Staff on site delegation logs

Country	Site	Names	Role
~~	~~~	Ms Emily Davies	Research Nurse
~~	~~~	Ms Gemma Austin (nee Glover)	Research Nurse
~~	~~~	Ms Claire Pratt	Trial Coordinator
~~	~~~	Ms Fatima Murad	Trial Coordinator
~~	~~~	Mr James Goulding	Administrator
~~	~~~	Ms Eleanor Moore	Administrator
~~	~~~	Ms Gill Smith	MDT coordinator
UK	Leicester: Leicester Royal Inf	Dr Subramanian Vasanthan	Site PI
~~	~~~	Dr Christopher Kent	Clinical/Surgical
~~	~~~	Dr Lesley Speed	Clinical/Surgical
~~	~~~	Mr Leyshon Griffiths	Clinical/Surgical
~~	~~~	Mr Roger Kockelbergh	Clinical/Surgical
~~	~~~	Ms Janet Potterton	Point of contact (1st)
~~	~~~	Ms Sallyanne Christmas	Point of contact (2nd)
~~	~~~	Mrs Julia Walker	Research Nurse
~~	~~~	Ms Jill Cooke	Research Nurse
~~	~~~	Ms Amy Branson (née Dineen)	Research Asst
~~	~~~	Ms Amy King	Administrator
UK	Lincoln: Lincoln County Hosp	Dr Thiagarajan Sreenivasan	Site PI
~~	~~~	Dr Karin Baria	Clinical/Surgical
~~	~~~	Dr Miguel Panades	Clinical/Surgical
~~	~~~	Mr Ian Mark	Clinical/Surgical
~~	~~~	Mr Nazeer Dahar	Clinical/Surgical
~~	~~~	Mr Pallon Daruwala	Clinical/Surgical
~~	~~~	Mrs Olesya Francis	Point of contact (1st)
~~	~~~	Ms Kathryn Hoare	Point of contact (1st)
~~	~~~	Ms Rachel Newton	Point of contact (1st)
~~	~~~	Mr Andrew Sloan	Point of contact (2nd)
~~	~~~	Mr Simon Archer	Research Nurse
~~	~~~	Ms Claire Key	Research Nurse
~~	~~~	Ms Helen Ginnelly	Research Nurse
~~	~~~	Ms Sarah Coombs	Research Nurse
~~	~~~	Miss Samantha Bateman	Data Manager
~~	~~~	Mrs Jane Hall	Radiographer

INVESTIGATORS AND COLLABORATORS: SITE STAFF
Staff on site delegation logs

Country	Site	Names	Role
~~	~~~	Mrs Maryanne Okubanjo	Radiographer
~~	~~~	Ms Carol Lockwood	Radiographer
~~	~~~	Ms Amy Cunningham	Other
UK	Liverpool: Royal Liverpool University Hosp	Dr Zafar Malik	Site PI
~~	~~~	Dr Peter Robson	Clinical/Surgical
~~	~~~	Miss Lynsey Dean	Point of contact (1st)
~~	~~~	Mr Thomas Rogers	Point of contact (1st)
~~	~~~	Miss Katy Treherne	Research Nurse
~~	~~~	Ms Nicola Bermingham	Research Nurse
~~	~~~	Ms Pauline Pilkington	Research Nurse
~~	~~~	Ms Heather Rogers	Other
UK	London: Charing Cross Hosp	Dr Alison Falconer	Site PI
~~	~~~	Dr May Stancliffe	Clinical/Surgical
~~	~~~	Dr Sathish Harinarayanan	Clinical/Surgical
~~	~~~	Dr Simon Stewart	Clinical/Surgical
~~	~~~	Mr Ross Dalton-Short	Point of contact (1st)
~~	~~~	Mr Steve Edwards	Point of contact (1st)
~~	~~~	Ms Sarah Rezkallah	Point of contact (1st)
~~	~~~	Ms Sue McInerney	Point of contact (2nd)
~~	~~~	Nebah Hassan	Point of contact (2nd)
~~	~~~	Ms Anne-Maree Thoi	Research Nurse
~~	~~~	Ms Ibiyemi Sadare (Olaleye)	Research Nurse
~~	~~~	Ms Amy Ford	Trial Coordinator
~~	~~~	Ms Bindu Chikkamuniyappa	Trial Coordinator
~~	~~~	Ms Gillian Hornzee	Trial Coordinator
~~	~~~	Miss Anna Westrop	Administrator
~~	~~~	Mr Bhavesh Pratap	Administrator
~~	~~~	Ms Sophia Magwaro	Radiographer
~~	~~~	Ms Amie Bourke	Other
UK	London: Croydon University Hosp	Mr Babbin John	Site PI
~~	~~~	Dr Adham Hijab	Clinical/Surgical
~~	~~~	Dr Alison Tree	Clinical/Surgical
~~	~~~	Dr Miguel Ferreira	Clinical/Surgical
~~	~~~	Dr Priyanka Patel	Clinical/Surgical

INVESTIGATORS AND COLLABORATORS: SITE STAFF

Staff on site delegation logs

Country	Site	Names	Role
~~	~~~	Dr Robert Huddart	Clinical/Surgical
~~	~~~	Mr Matthew Perry	Clinical/Surgical
~~	~~~	Mr Nasr Arsanious	Clinical/Surgical
~~	~~~	Ms Claire Crowley	Clinical/Surgical
~~	~~~	Ms Ibiyemi Sadare (Olaleye)	Point of contact (1st)
~~	~~~	Mrs Yvonne Campbell	Point of contact (2nd)
~~	~~~	Ms Shaki Balogun	Point of contact (2nd)
~~	~~~	Mrs Ann Payne	Research Nurse
~~	~~~	Ms Anne Haldeos	Research Nurse
~~	~~~	Ms Cheryl Batish	Research Nurse
~~	~~~	Ms Christine Springall	Research Nurse
~~	~~~	Ms Jane Thompson	Research Nurse
~~	~~~	Sister Jane Thomson	Research Nurse
~~	~~~	Mrs Maria Serra	Research Asst
~~	~~~	Ms Jackie Pach	Administrator
~~	~~~	Ms Sheefa Ahamadali	MDT coordinator
~~	~~~	Dr Andriana Michaelidou	Other
~~	~~~	Mr Mohammed Nawrozzadeh	Other
~~	~~~	Ms Emma Dunne	Other
UK	London: Guy's Hosp (London)	Dr Stephen Morris	Site PI
~~	~~~	Dr Kannon Nathan	Clinical/Surgical
~~	~~~	Dr Ronald Beaney	Clinical/Surgical
~~	~~~	Dr Sarah Harris	Clinical/Surgical
~~	~~~	Dr Simon Hughes	Clinical/Surgical
~~	~~~	Dr Teresa Guerrero-Urbano	Clinical/Surgical
~~	~~~	Ms Gabriella Assante	Point of contact (1st)
~~	~~~	Ms Jessica Rashid	Point of contact (1st)
~~	~~~	Ms Katie Jones	Point of contact (1st)
~~	~~~	Ms Viviana Aya	Point of contact (1st)
~~	~~~	Miss Lorna Bower	Research Nurse
~~	~~~	Mr Brendan Hore	Trial Coordinator
~~	~~~	Mr Greg Kuenzig	Trial Coordinator
~~	~~~	Mrs Vesna Hogan	Trial Coordinator
~~	~~~	Ms Gabriella Assante	Trial Coordinator

INVESTIGATORS AND COLLABORATORS: SITE STAFF
Staff on site delegation logs

Country	Site	Names	Role
~~	~~~	Ms Isabel Grau	Trial Coordinator
~~	~~~	Mr Hussain Gordon	Research Asst
~~	~~~	Miss Lisa-Jane Conway	Radiographer
~~	~~~	Mr Philip Reynolds	Radiographer
~~	~~~	Ms Sally Donaghey	Radiographer
~~	~~~	Bali Rooprai	Other
~~	~~~	Mrs Dawn Nunney	Other
UK	London: Hammersmith Hosp	Dr Stephen Mangar	Site PI
~~	~~~	Dr Simon Stewart	Clinical/Surgical
~~	~~~	Mr Ross Dalton-Short	Point of contact (1st)
~~	~~~	Mr Steve Edwards	Point of contact (1st)
~~	~~~	Ms Sarah Rezkallah	Point of contact (1st)
~~	~~~	Ms Bindu Chikkamuniyappa	Point of contact (2nd)
~~	~~~	Nebah Hassan	Point of contact (2nd)
~~	~~~	Ms Anne-Maree Thoi	Research Nurse
~~	~~~	Miss Anna Westrop	Administrator
~~	~~~	Ms Sophia Magwaro	Radiographer
UK	London: North Middlesex Hosp	Dr Nishi Gupta	Site PI
~~	~~~	Dr Farhad Neave	Clinical/Surgical
~~	~~~	Dr Jackie Newby	Clinical/Surgical
~~	~~~	Dr Lucinda Melcher	Clinical/Surgical
~~	~~~	Dr Stephen Karp	Clinical/Surgical
~~	~~~	Miss Sagal Kullane	Point of contact (1st)
~~	~~~	Ms Judy Hill	Research Nurse
~~	~~~	Miss Chloe Van Someren	Data Manager
~~	~~~	Mr Tom Caumont	Data Manager
~~	~~~	Ms Hanna Azirar	Data Manager
~~	~~~	Ms Tina Macavoy	MDT coordinator
UK	London: Royal Free Hosp	Dr Sarah Needleman	Site PI
~~	~~~	Dr Katherine Pigott	Clinical/Surgical
~~	~~~	Dr Maria Vilarino-Varela	Clinical/Surgical
~~	~~~	Dr Nicola Rosenfelder	Clinical/Surgical
~~	~~~	Ms Alexandra Gore	Point of contact (1st)
~~	~~~	Ms Claire Jarvis	Point of contact (1st)

INVESTIGATORS AND COLLABORATORS: SITE STAFF
Staff on site delegation logs

Country	Site	Names	Role
~~	~~~	Ms Hannah Powell	Point of contact (1st)
~~	~~~	Mrs Kaliyanee Ramtohul	Research Nurse
~~	~~~	Ms Angela McCadden	Research Nurse
~~	~~~	Ms Juniebel Cooke	Research Nurse
~~	~~~	Ms Lynda Annan	Research Nurse
~~	~~~	Ms Sara Fawcitt	Research Nurse
~~	~~~	Ms Sylvia Grieve	Research Nurse
~~	~~~	Miss Emma Douch	Trial Coordinator
~~	~~~	Ms Naomi Anderson	Data Manager
UK	London: Royal Marsden Hosp (London)	Dr Vincent Khoo	Site PI
~~	~~~	Dr Alison Tree	Clinical/Surgical
~~	~~~	Dr Liam Welsh	Clinical/Surgical
~~	~~~	Dr Nicholas Van As	Clinical/Surgical
~~	~~~	Miss Holly Hogan	Point of contact (1st)
~~	~~~	Miss Jennyfa Ali	Point of contact (1st)
~~	~~~	Mr Bernard Siu	Point of contact (1st)
~~	~~~	Mrs Sijy Pillai	Point of contact (1st)
~~	~~~	Ms Giulia Carlino	Point of contact (1st)
~~	~~~	Mr Joseph Montebello	Point of contact (2nd)
~~	~~~	Miss Vijitha Vijayakumar	Research Nurse
~~	~~~	Mr Trevor Bott	Research Nurse
~~	~~~	Mrs Debra Townsend-Thorn	Research Nurse
~~	~~~	Mrs Ruth Stafferton	Research Nurse
~~	~~~	Miss Hanna Bryant	Trial Coordinator
~~	~~~	Mr Emmanuel Brown	Trial Coordinator
~~	~~~	Ms Cordelia Grant	Trial Coordinator
~~	~~~	Ms Laillah-Crystal Banda	Trial Coordinator
~~	~~~	Miss Maryam Ali	Administrator
~~	~~~	Mr Chintan Mojidra	Administrator
~~	~~~	Mrs Sarah Storrs	Administrator
~~	~~~	Mr Matthew Olabanji	Other
~~	~~~	Ms Jennifer Morrison	Other
UK	London: Royal Marsden Hosp (Sutton)	Dr Chris Parker	Site PI
~~	~~~	Dr Ramachandran Venkitaraman	Clinical/Surgical

INVESTIGATORS AND COLLABORATORS: SITE STAFF
Staff on site delegation logs

Country	Site	Names	Role
~~	~~~	Dr Robert Huddart	Clinical/Surgical
~~	~~~	Prof Alan Horwich	Clinical/Surgical
~~	~~~	Prof David Dearnaley	Clinical/Surgical
~~	~~~	Mrs Annie Gao	Point of contact (1st)
~~	~~~	Ms Louise Murphy	Point of contact (1st)
~~	~~~	Mr John Marshall	Trial Coordinator
~~	~~~	Ms Val Lewington	Trial Coordinator
UK	London: St Bartholomews Hosp (London)	Dr Paula Wells	Site PI
~~	~~~	Dr Alexandre Kaliski	Clinical/Surgical
~~	~~~	Dr Karen Tipples	Clinical/Surgical
~~	~~~	Mrs Resmi Jayachandran	Point of contact (1st)
~~	~~~	Ms Olivia Bolton	Point of contact (1st)
~~	~~~	Mr Paul Hillman	Research Nurse
~~	~~~	Ms Janet Kiff	Research Nurse
~~	~~~	Mr Fatjon Dekaj	Trial Coordinator
~~	~~~	Mr Alastair Nicholson	Data Manager
~~	~~~	Mr Jude Nixon	Data Manager
~~	~~~	Ms Janet Oladimeji	Data Manager
~~	~~~	Ms Nanette Bech-Nielsen	Research Asst
~~	~~~	Mr Oscar Riches	Radiographer
~~	~~~	Mrs Samantha Chetiyawardana	Radiographer
~~	~~~	Dr Dan Smith	Other
~~	~~~	Dr John Conibear	Other
~~	~~~	Dr Kirsty Beaton	Other
UK	London: St Georges Hosp (London)	Mr Rami Issa	Site PI
~~	~~~	Dr Vincent Khoo	Clinical/Surgical
~~	~~~	Mr Chris Anderson	Clinical/Surgical
~~	~~~	Mr Matthew Perry	Clinical/Surgical
~~	~~~	Miss Claire Gilmartin	Point of contact (1st)
~~	~~~	Miss Serena Dover	Point of contact (1st)
~~	~~~	Mr Juel Tuazon	Point of contact (1st)
~~	~~~	Ms Sophie Golden	Point of contact (1st)
~~	~~~	Miss Jesusa Toledo	Research Nurse
~~	~~~	Mr Robert Varro	Research Nurse

INVESTIGATORS AND COLLABORATORS: SITE STAFF
Staff on site delegation logs

Country	Site	Names	Role
~~	~~~	Ms Helen Tighe	Research Nurse
~~	~~~	Ms Jane Gregg	Research Nurse
~~	~~~	Mr Mark Quarrell	Trial Coordinator
~~	~~~	Ms Chandni Patel	Trial Coordinator
~~	~~~	Ms Deirdre Daly	Data Manager
~~	~~~	Miss Titilayo Oni	Administrator
~~	~~~	Ms Debbie Rolfe	Pharmacist
UK	London: St Marys Hosp (London)	Dr Simon Stewart	Site PI
~~	~~~	Mr Mark Caballes	Point of contact (1st)
~~	~~~	Mrs Manisha Joshi	Point of contact (1st)
~~	~~~	Ms Emily Russell	Point of contact (1st)
~~	~~~	Ms Stephanie Ivie	Point of contact (1st)
~~	~~~	Ms Anne-Maree Thoi	Research Nurse
~~	~~~	Mr Farhan Naim	Trial Coordinator
~~	~~~	Ms Byiravey Pathmanathan	Trial Coordinator
~~	~~~	Ms Gillian Hornzee	Trial Coordinator
~~	~~~	Ms Joy Liau	Trial Coordinator
~~	~~~	Mr Vikram Bohra	Data Manager
~~	~~~	Ms Laura Custins	Data Manager
~~	~~~	Miss Anna Westrop	Administrator
~~	~~~	Mr Anup Patel	Administrator
~~	~~~	Ms Anna Tippins	MDT coordinator
~~	~~~	Ms Sophia Magwaro	Radiographer
UK	London: University College Hosp	Dr Heather Payne	Site PI
~~	~~~	Dr Reena Davda	Site PI
~~	~~~	Dr Ajay Aggarwal	Clinical/Surgical
~~	~~~	Dr Anita Mitra	Clinical/Surgical
~~	~~~	Dr David Woolf	Clinical/Surgical
~~	~~~	Dr Jonathan Teh	Clinical/Surgical
~~	~~~	Dr Julia Hall	Clinical/Surgical
~~	~~~	Dr Rachel Khong	Clinical/Surgical
~~	~~~	Mr Thomas Amoaten	Point of contact (1st)
~~	~~~	Ms Nicole Bonsu	Point of contact (1st)
~~	~~~	Ms Patricia Danaswamy	Point of contact (1st)

INVESTIGATORS AND COLLABORATORS: SITE STAFF
Staff on site delegation logs

Country	Site	Names	Role
~~	~~~	Ms Suzy Lowi	Point of contact (1st)
~~	~~~	Ms Didem Agdiran	Point of contact (2nd)
~~	~~~	Ms Helene Zilkha	Point of contact (2nd)
~~	~~~	Ms Samantha Whinn	Point of contact (2nd)
~~	~~~	Ms Annelies Gillesen	Research Nurse
~~	~~~	Mr Richard Merrick	Trial Coordinator
~~	~~~	Mrs Roshni Goel	Data Manager
~~	~~~	Ms Natasha Aslam	Data Manager
~~	~~~	Ms Zainab Butt	Radiographer
UK	London: Whipps Cross University Hosp	Mr James Green	Site PI
~~	~~~	Dr David Woolf	Clinical/Surgical
~~	~~~	Dr Paula Wells	Clinical/Surgical
~~	~~~	Mr John Hines	Clinical/Surgical
~~	~~~	Mr John Peters	Clinical/Surgical
~~	~~~	Mr Simon Holden	Clinical/Surgical
~~	~~~	Mr Timothy Philp	Clinical/Surgical
~~	~~~	Mrs Resmi Jayachandran	Point of contact (1st)
~~	~~~	Ms Olivia Bolton	Point of contact (1st)
~~	~~~	Miss Memory Kazingizi	Research Nurse
~~	~~~	Mr John O'Neill	Research Nurse
~~	~~~	Mr Paul Hillman	Research Nurse
~~	~~~	Mr Thompson Olaoni	Research Nurse
~~	~~~	Ms Emma Foster	Research Nurse
~~	~~~	Ms Linda Dawson-Athey	Research Nurse
~~	~~~	Ms Patricia Danaswamy	Research Nurse
~~	~~~	Ms Sadaf Zaidi	Trial Coordinator
~~	~~~	Ms Nanette Bech-Nielsen	Research Asst
~~	~~~	Mr Andrew Gillian	Pharmacist
UK	Macclesfield: Macclesfield District General Hosp	Mr Richard Brough	Site PI
~~	~~~	Mr Adebanji Adeyoju	Clinical/Surgical
~~	~~~	Mr Gerald Collins	Clinical/Surgical
~~	~~~	Mr Stephen CW Brown	Clinical/Surgical
~~	~~~	Mr Waheed Zafar	Clinical/Surgical
~~	~~~	Ms Magda Kujawa	Clinical/Surgical

INVESTIGATORS AND COLLABORATORS: SITE STAFF

Staff on site delegation logs

Country	Site	Names	Role
~~	~~~	Mrs Pippa Hill	Point of contact (1st)
~~	~~~	Mrs Victoria Adinkra	Research Nurse
~~	~~~	Ms Barbara Townley	Research Nurse
~~	~~~	Mr Iain Woodhouse	Trial Coordinator
~~	~~~	Ms Marilyn McCurrie	Administrator
~~	~~~	Ms Lisa Hardstaff	Other
UK	Maidstone: Maidstone Hosp	Dr Henry Taylor	Site PI
~~	~~~	Dr Carys Thomas	Clinical/Surgical
~~	~~~	Dr Christos Mikropoulos	Clinical/Surgical
~~	~~~	Mr John Donohue	Clinical/Surgical
~~	~~~	Dr Kathryn Lees	Clinical/Surgical
~~	~~~	Dr Patryk Brulinski	Clinical/Surgical
~~	~~~	Dr Sharon Beesley	Clinical/Surgical
~~	~~~	Mrs Alison Davison	Point of contact (1st)
~~	~~~	Ms Verity Roberts	Point of contact (1st)
~~	~~~	Mrs Ann Phillips	Research Nurse
~~	~~~	Mrs Jane Murray	Research Nurse
~~	~~~	Ms Barbara Mercier	Research Nurse
~~	~~~	Ms Carmel Jope	Research Nurse
~~	~~~	Ms Lisa Tribe	Research Nurse
~~	~~~	Ms Sarah Martins	Research Nurse
~~	~~~	Ms Vivienne Breen	Research Nurse
~~	~~~	Ms Yvonne Lines	Trial Coordinator
~~	~~~	Mr Gavin Fossey	Administrator
~~	~~~	Ms Clare Calvert	Administrator
~~	~~~	Mr Ian Pamphlett	Pharmacist
~~	~~~	Dr Pauline Wood	Radiographer
~~	~~~	Miss Katy Taylor	Radiographer
~~	~~~	Mr Innocent Neshiri	Radiographer
~~	~~~	Mrs Heather Dias	Radiographer
~~	~~~	Ms Jodie Hotine	Radiographer
~~	~~~	Ms Pavnish Rai	Radiographer
~~	~~~	Ms Sarah Rezkallah	Radiographer
~~	~~~	Mr Ifan Jones	Other

INVESTIGATORS AND COLLABORATORS: SITE STAFF

Staff on site delegation logs

Country	Site	Names	Role
~~	~~~	Ms Su Burrage	Other
UK	Manchester: Christie Hosp	Dr John Logue	Site PI
~~	~~~	Dr Ananya Choudhury	Clinical/Surgical
~~	~~~	Dr Jacqueline Livsey	Clinical/Surgical
~~	~~~	Dr James Wylie	Clinical/Surgical
~~	~~~	Dr Richard Cowan	Clinical/Surgical
~~	~~~	Dr Ruth Conroy	Clinical/Surgical
~~	~~~	Dr Tony Elliott	Clinical/Surgical
~~	~~~	Miss Amber Hart	Point of contact (1st)
~~	~~~	Ms Roonak Nazari	Point of contact (1st)
~~	~~~	Ms Sarah-Ellen Smith	Point of contact (1st)
~~	~~~	Ms Lucy Worsley	Point of contact (2nd)
~~	~~~	Miss Kate O'Connor	Research Nurse
~~	~~~	Mr Damian McCaul	Research Nurse
~~	~~~	Ms Carol Newbery	Research Nurse
~~	~~~	Ms Catherine Redshaw	Research Nurse
~~	~~~	Sister Jackie O'Dwyer	Research Nurse
~~	~~~	Sister Viv Thomas	Research Nurse
~~	~~~	Miss Holly White	Trial Coordinator
~~	~~~	Miss Laura Flanagan	Trial Coordinator
~~	~~~	Miss Maria Petsa	Trial Coordinator
~~	~~~	Miss Willemijn Spoor	Trial Coordinator
~~	~~~	Mr Adrian Fallaize	Trial Coordinator
~~	~~~	Mr Ekugbe Onoge	Trial Coordinator
~~	~~~	Mr Ian Duncan	Trial Coordinator
~~	~~~	Ms Joanne Oliver	Trial Coordinator
~~	~~~	Ms Viviana Carpio	Trial Coordinator
~~	~~~	Miss Sarah Green	Data Manager
~~	~~~	Mr Ian Bottomley	Data Manager
~~	~~~	Miss Kim Fair	Administrator
~~	~~~	Mrs Sue Davison	Administrator
~~	~~~	Mr Jonathan Buchan	MDT coordinator
~~	~~~	Ms Catherine Harris	Other
~~	~~~	Ms Cathryn Jones	Other

INVESTIGATORS AND COLLABORATORS: SITE STAFF
Staff on site delegation logs

Country	Site	Names	Role
UK	Manchester: Withington Hosp	Mr Vijay Sangar	Site PI
~~	~~~	Dr James Wylie	Clinical/Surgical
~~	~~~	Mr Vijay Ramani	Clinical/Surgical
~~	~~~	Ms Heena Mistry	Point of contact (1st)
~~	~~~	Ms Linda Bailey	Point of contact (1st)
~~	~~~	Ms Lindsay Piper	Point of contact (1st)
~~	~~~	Ms Molly Bennett	Point of contact (1st)
~~	~~~	Ms Kathryn Slevin	Point of contact (2nd)
~~	~~~	Miss Anna Gipson	Research Nurse
~~	~~~	Miss Fiona McCarth	Research Nurse
~~	~~~	Mrs Helen Haydock	Research Nurse
~~	~~~	Ms Fiona McCartin	Research Nurse
~~	~~~	Ms Kathryn Fellows	Research Nurse
~~	~~~	Ms Sarah Liptrott	Research Nurse
~~	~~~	Ms Thobekile Mthethwa	Research Nurse
~~	~~~	Ms Annie Duffy	Trial Coordinator
~~	~~~	Ms Sarah Sahin	Trial Coordinator
~~	~~~	Ms Rebecca Corless	Data Manager
~~	~~~	Ms Lillian Partington	Pharmacist
~~	~~~	Dr Jonathan Tuck	Other
~~	~~~	Dr WF Knox	Other
UK	Middlesbrough: James Cook University Hosp	Dr Clive Peedell	Site PI
~~	~~~	Dr David Wilson	Clinical/Surgical
~~	~~~	Dr Devadasan Shakespeare	Clinical/Surgical
~~	~~~	Dr Hans Van der Voet	Clinical/Surgical
~~	~~~	Dr John Hardman	Clinical/Surgical
~~	~~~	Dr Julia McBride	Clinical/Surgical
~~	~~~	Mr David Chadwick	Clinical/Surgical
~~	~~~	Ms Rita Mohan	Point of contact (1st)
~~	~~~	Mr Keith Harland	Point of contact (2nd)
~~	~~~	Mrs Alison Barnes	Research Nurse
~~	~~~	Mrs Carol Long	Research Nurse
~~	~~~	Ms Lorraine Atkinson	Research Nurse
~~	~~~	Sister Patricia McClurey	Research Nurse

INVESTIGATORS AND COLLABORATORS: SITE STAFF
Staff on site delegation logs

Country	Site	Names	Role
~~	~~~	Mrs Alison Chilvers	Trial Coordinator
~~	~~~	Ms Alison Chilvers	Trial Coordinator
~~	~~~	Ms Sarah McAuliffe	Trial Coordinator
~~	~~~	Ms Lynne Naylor	Administrator
~~	~~~	Ms Sarah Barnbrooke	MDT coordinator
~~	~~~	Mrs Paula Robson	Radiographer
~~	~~~	Ms Emma Thompson	Radiographer
~~	~~~	Ms Caroline Brownless	Other
UK	Newcastle: Freeman Hosp	Dr Ian Pedley	Site PI
~~	~~~	Dr John Frew	Clinical/Surgical
~~	~~~	Dr Rhona McMenemin	Clinical/Surgical
~~	~~~	Dr Trevor Roberts	Clinical/Surgical
~~	~~~	Miss Janine Tate	Point of contact (1st)
~~	~~~	Mr Jonathan Stoddart	Point of contact (1st)
~~	~~~	Ms Jayashree Walker	Trial Coordinator
~~	~~~	Miss Danielle Riseborough	Data Manager
~~	~~~	Miss Katy Lambert	Data Manager
~~	~~~	Miss Emma Stockley	Radiographer
~~	~~~	Mr Chris Barron	Radiographer
~~	~~~	Ms Bridget Workman	Other
UK	Newport: Royal Gwent Hosp	Mr Jim Wilson	Site PI
~~	~~~	Mr Adam Carter	Clinical/Surgical
~~	~~~	Mr Adam Cox	Clinical/Surgical
~~	~~~	Mr Syhed Rahman	Clinical/Surgical
~~	~~~	Miss Maxine Nash	Point of contact (1st)
~~	~~~	Mr Simon Hodge	Research Nurse
~~	~~~	Mrs Debra Barnett	Research Nurse
~~	~~~	Ms Julie Simpson	Research Nurse
~~	~~~	Ms Karen Wild	Research Nurse
~~	~~~	Ms Rachel Williams	Research Nurse
~~	~~~	Ms S Kearney	Research Nurse
~~	~~~	Ms Alison Davey	Trial Coordinator
~~	~~~	Ms Janet Marty	Trial Coordinator
~~	~~~	Ms Kirstin Davies	Trial Coordinator

INVESTIGATORS AND COLLABORATORS: SITE STAFF

Staff on site delegation logs

Country	Site	Names	Role
~~	~~~	Ms Lisa Gilby	Trial Coordinator
~~	~~~	Mrs Jayne Richards	Data Manager
~~	~~~	Mrs Paula Webb	Data Manager
~~	~~~	Ms Helene Lavender	Data Manager
~~	~~~	Ms Elaine Wall	Administrator
UK	Northampton: Northampton General Hosp	Dr Jenny Branagan	Site PI
~~	~~~	Dr Christine Elwell	Clinical/Surgical
~~	~~~	Dr Dorai Ramanathan	Clinical/Surgical
~~	~~~	Dr Philip Camilleri	Clinical/Surgical
~~	~~~	Mrs Ruby Goyena	Point of contact (1st)
~~	~~~	Ms Maxine Foo	Point of contact (1st)
~~	~~~	Ms Rachel Tighe (nee Bussey)	Point of contact (1st)
~~	~~~	Jane O’Callaghan	Point of contact (2nd)
~~	~~~	Ms Andrea Jones	Point of contact (2nd)
~~	~~~	Mrs Katherine McGrath	Research Nurse
~~	~~~	Ms Gillian Dell	Research Nurse
~~	~~~	Ms Bronwen Thominson	Administrator
~~	~~~	Mrs Elizabeth Tee	Other
~~	~~~	Ms Hazel McBain	Other
UK	Norwich: Norfolk & Norwich University Hosp	Dr Rob Wade	Site PI
~~	~~~	Dr Jenny Nobes	Clinical/Surgical
~~	~~~	Dr Joe Ostrowski	Clinical/Surgical
~~	~~~	Mr Guarav Kapur	Clinical/Surgical
~~	~~~	Mrs Adele Cooper	Clinical/Surgical
~~	~~~	Mrs Suzanne Walker	Clinical/Surgical
~~	~~~	Ms Karen Heasley	Clinical/Surgical
~~	~~~	Ms Cheryl Websdale	Point of contact (1st)
~~	~~~	Ms Sara Callam	Point of contact (1st)
~~	~~~	Ms Sarah Turner	Point of contact (1st)
~~	~~~	Ms Denise Archer	Point of contact (2nd)
~~	~~~	Dr Jane Beety	Trial Coordinator
~~	~~~	Mrs Joanna White	Trial Coordinator
~~	~~~	Mrs Sharon Walton	Trial Coordinator
~~	~~~	Ms Bridget Shobrook	Trial Coordinator

INVESTIGATORS AND COLLABORATORS: SITE STAFF

Staff on site delegation logs

Country	Site	Names	Role
~~	~~~	Ms Helen Darby	Trial Coordinator
~~	~~~	Mr Mark Bloomfield	Data Manager
~~	~~~	Mr Richard Birch	Data Manager
~~	~~~	Mrs Katrien Oosterom	Data Manager
~~	~~~	Miss Clare London	Pharmacist
~~	~~~	Miss Gail Healey	Pharmacist
UK	Nottingham: Nottingham University Hosps, City Campus	Dr Georgina Walker	Site PI
~~	~~~	Dr Ian Sayers	Clinical/Surgical
~~	~~~	Dr Jamie Mills	Clinical/Surgical
~~	~~~	Dr Santhanam Sundar	Clinical/Surgical
~~	~~~	Miss Leanne Alder	Point of contact (1st)
~~	~~~	Ms Hazel Marley	Point of contact (1st)
~~	~~~	Ms Cody Jevons	Point of contact (2nd)
~~	~~~	Ms Rachael Chivers	Point of contact (2nd)
~~	~~~	Miss Lucy Howard	Research Nurse
~~	~~~	Ms Hanna Purves	Research Nurse
~~	~~~	Mrs Catherine Wood	Administrator
~~	~~~	Mrs Carol Gooch	Radiographer
~~	~~~	Miss Camille Hutchinson	Other
~~	~~~	Mr Daniel Kumar	Other
~~	~~~	Mr Jacob Szolin-Jones	Other
UK	Oldham: Royal Oldham Hosp	Dr Jacqueline Livsey	Site PI
~~	~~~	Dr Ruth Conroy	Site PI
~~	~~~	Dr Ananya Choudhury	Clinical/Surgical
~~	~~~	Ms Lyndsay Scarratt	Point of contact (1st)
~~	~~~	Ms Dawn Johnstone	Point of contact (2nd)
~~	~~~	Ms Joanne Johnson	Point of contact (2nd)
~~	~~~	Mr Richard Jones	Research Nurse
~~	~~~	Ms Wendy Cook	Research Nurse
~~	~~~	Mrs Leena Mistry	Trial Coordinator
~~	~~~	Ms Lisa Gill	Trial Coordinator
UK	Oxford: Churchill Hosp	Dr Philip Camilleri	Site PI
~~	~~~	Dr Ahmad Sabbagh	Clinical/Surgical
~~	~~~	Dr Ami Sabharwal	Clinical/Surgical

INVESTIGATORS AND COLLABORATORS: SITE STAFF
Staff on site delegation logs

Country	Site	Names	Role
~~	~~~	Dr Anne Kiltie	Clinical/Surgical
~~	~~~	Dr David J Cole	Clinical/Surgical
~~	~~~	Dr Gagan Bhatnagar	Clinical/Surgical
~~	~~~	Dr Gerard Andrade	Clinical/Surgical
~~	~~~	Dr Hiba Al-Chamali	Clinical/Surgical
~~	~~~	Dr Katherine Hyde	Clinical/Surgical
~~	~~~	Dr Michael Skwarski	Clinical/Surgical
~~	~~~	Dr Niki Panakis	Clinical/Surgical
~~	~~~	Dr Raj Jampana	Clinical/Surgical
~~	~~~	Dr Rob Owens	Clinical/Surgical
~~	~~~	Dr Robert Stuart	Clinical/Surgical
~~	~~~	Mr Daniel Ajzensztejn	Clinical/Surgical
~~	~~~	Mr Simon Brewster	Clinical/Surgical
~~	~~~	Miss Patrycja Jastrzebska	Point of contact (1st)
~~	~~~	Miss Weronika Rabsztyn	Point of contact (1st)
~~	~~~	Mrs Kerrie Doyle	Point of contact (1st)
~~	~~~	Ms Sandra Mukkath	Point of contact (1st)
~~	~~~	Ms Sylwia Bekulart	Point of contact (1st)
~~	~~~	Mrs Ann Murphy	Research Nurse
~~	~~~	Mrs Anne Butterfield	Research Nurse
~~	~~~	Mrs Jo Wilson	Research Nurse
~~	~~~	Mrs Kerrie Marston	Research Nurse
~~	~~~	Ms Jane Boutflower	Research Nurse
~~	~~~	Ms Phillippa Kyffin	Research Nurse
~~	~~~	Ms Sarah Lawrey	Research Nurse
~~	~~~	Ms Sarah Markus	Research Nurse
~~	~~~	Mr Dave Barber	Trial Coordinator
~~	~~~	Ms Abimbola Aiku	Trial Coordinator
~~	~~~	Mr Matthew Mooney	Data Manager
~~	~~~	Mr Naveen Sankighatta	Data Manager
~~	~~~	Mr Tim Coutts	Data Manager
~~	~~~	Ms Trish Green	Data Manager
~~	~~~	Ms Nicole Langridge	Administrator
~~	~~~	Miss Grace Samkange	Radiographer

INVESTIGATORS AND COLLABORATORS: SITE STAFF
Staff on site delegation logs

Country	Site	Names	Role
~~	~~~	Miss Lisa Durrant	Radiographer
~~	~~~	Mrs Sarah Ruane	Radiographer
~~	~~~	Ms Weronika Carroll	Radiographer
~~	~~~	Ms Sandie Wellman	Other
UK	Peterborough: Peterborough City Hosp	Dr Abigail Hollingdale	Site PI
~~	~~~	Ms Kerrie Cavanagh	Point of contact (1st)
UK	Peterborough: Peterborough District Hosp	Dr Debbie Gregory	Site PI
~~	~~~	Dr Charlotte Ingle	Clinical/Surgical
~~	~~~	Dr Richard Benson	Clinical/Surgical
~~	~~~	Ms Elisa Barter	Clinical/Surgical
~~	~~~	Ms Paula-Joanne Rooney	Research Nurse
~~	~~~	Ms Susan Allen	Trial Coordinator
~~	~~~	Miss Steph Lawrence	Administrator
UK	Plymouth: Derriford Hosp	Mr Henry Sells	Site PI
~~	~~~	Dr Esther McLarty	Clinical/Surgical
~~	~~~	Dr Salvatore Natale	Clinical/Surgical
~~	~~~	Dr Sarah Pascoe	Clinical/Surgical
~~	~~~	Mr John Christopher Hammonds	Clinical/Surgical
~~	~~~	Mr Paul McInerney	Clinical/Surgical
~~	~~~	Mr Keith Purcell	Point of contact (1st)
~~	~~~	Ms Lyn Cogley	Point of contact (1st)
~~	~~~	Miss Emma Bishop	Point of contact (2nd)
~~	~~~	Ms Sharah Tyner	Point of contact (2nd)
~~	~~~	Ms Victoria yates	Point of contact (2nd)
~~	~~~	Ms Maria Brennan	Research Nurse
UK	Poole: Poole Hosp	Dr Joe Davies	Site PI
~~	~~~	Dr Perric Crellin	Clinical/Surgical
~~	~~~	Dr Sathish Harinarayanan	Clinical/Surgical
~~	~~~	Dr Sue Brock	Clinical/Surgical
~~	~~~	Mrs Louise Heckford	Point of contact (1st)
~~	~~~	Ms Sophie Rix	Point of contact (1st)
~~	~~~	Mr Neal Beamish	Point of contact (2nd)
~~	~~~	Mr Roger Wheelwright	Research Nurse
~~	~~~	Ms Amanda Iskender	Research Nurse

INVESTIGATORS AND COLLABORATORS: SITE STAFF
Staff on site delegation logs

Country	Site	Names	Role
~~	~~~	Ms Elizabeth Clarke	Research Nurse
~~	~~~	Ms Hilary Blaney	Research Nurse
~~	~~~	Ms Sandy Pressdee	Research Nurse
~~	~~~	Ms Seonaid Wright	Research Nurse
~~	~~~	Mrs Josie Goodsell	Trial Coordinator
~~	~~~	Miss Elizabeth Woodward	Data Manager
~~	~~~	Miss Nichola Downs	Data Manager
~~	~~~	Ms Sally Gillespie	Data Manager
~~	~~~	Ms Sara Orford	Data Manager
~~	~~~	Ms Teresa Coffin	Administrator
~~	~~~	Miss Felicity Clapp	Radiographer
UK	Portsmouth: Queen Alexandra Hosp	Dr Maja Uherek	Site PI
~~	~~~	Dr Yoodhvir Nagar	Site PI
~~	~~~	Dr Azarel Virgo	Clinical/Surgical
~~	~~~	Dr Ghassan Khoury	Clinical/Surgical
~~	~~~	Dr Kudingila Madhava	Clinical/Surgical
~~	~~~	Ms Mila Roca	Point of contact (1st)
~~	~~~	Mrs Jennifer Hale	Point of contact (2nd)
~~	~~~	Mrs Tracey Dobson	Point of contact (2nd)
~~	~~~	Ms Anna Stephenson	Point of contact (2nd)
~~	~~~	Mrs Angie Harris-Burland	Research Nurse
~~	~~~	Mrs Wendy Stacey	Research Nurse
~~	~~~	Ms Lorna Meadows	Administrator
~~	~~~	Ms Catrin Watkinson	Pharmacist
~~	~~~	Ms Kathy Blight	Pharmacist
UK	Preston: Royal Preston Hosp	Dr Alison Birtle	Site PI
~~	~~~	Dr Marcus Wise	Clinical/Surgical
~~	~~~	Dr Omi Parikh	Clinical/Surgical
~~	~~~	Ms Catherine Walmsley	Point of contact (1st)
~~	~~~	Ms Zainab Chauhan	Point of contact (2nd)
~~	~~~	Miss Rose Ellard	Research Nurse
~~	~~~	Mr Billy Hefferon	Research Nurse
~~	~~~	Mrs Claire Searle	Research Nurse
~~	~~~	Mrs Sandra Curtis	Research Nurse

INVESTIGATORS AND COLLABORATORS: SITE STAFF
Staff on site delegation logs

Country	Site	Names	Role
~~	~~~	Ms Stephanie Cornthwaite	Research Nurse
~~	~~~	Mr Nathan Fish	Data Manager
~~	~~~	Ms Helen Spickett	Data Manager
UK	Reading: Royal Berkshire Hosp	Dr Paul Rogers	Site PI
~~	~~~	Dr Richard B Brown	Clinical/Surgical
~~	~~~	Mr Stephen Parr	Point of contact (1st)
~~	~~~	Ms Emma Vowell	Point of contact (1st)
~~	~~~	Ms Jane Atkinson	Point of contact (1st)
~~	~~~	Mrs Christina Lewis	Point of contact (2nd)
~~	~~~	Mrs Wioletta Kowalczyk-Williams	Research Nurse
~~	~~~	Ms Allison Hunt	Research Nurse
~~	~~~	Ms Helen Purdon	Research Nurse
~~	~~~	Ms Kristy Coomber	Research Nurse
~~	~~~	Ms Debbie Cartwright	Trial Coordinator
UK	Redditch: Alexandra Hosp	Dr Lisa Capaldi	Site PI
~~	~~~	Dr Joanna Hamilton	Clinical/Surgical
~~	~~~	Mrs Alison Harrison	Point of contact (1st)
~~	~~~	Mrs Hayley Hodson	Point of contact (2nd)
~~	~~~	Mrs Sarah Moss	Research Nurse
~~	~~~	Mrs Wendy Featherstone	Research Nurse
~~	~~~	Ms Andrea Isaew	Research Nurse
~~	~~~	Ms Jeanette Knapp	Research Nurse
~~	~~~	Ms Margaret Hindle	MDT coordinator
UK	Romford: Queen's Hosp (Romford)	Dr Maria Martinou	Site PI
~~	~~~	Dr Stephanie Gibbs	Site PI
~~	~~~	Prof Saad Tahir	Site PI
~~	~~~	Dr Ramachandran Subramaniam	Clinical/Surgical
~~	~~~	Mr Anand Kelkar	Clinical/Surgical
~~	~~~	Mr Mohammad Tanvir Vandal	Clinical/Surgical
~~	~~~	Mr Sandeep Gujral	Clinical/Surgical
~~	~~~	Mr Neale O'Brien	Point of contact (1st)
~~	~~~	Thi Vu	Point of contact (1st)
~~	~~~	Mr Revanth Jannapureddy	Point of contact (2nd)
~~	~~~	Ms Tina Mills-Baldock	Research Nurse

INVESTIGATORS AND COLLABORATORS: SITE STAFF
Staff on site delegation logs

Country	Site	Names	Role
~~	~~~	Mr Alastair Nicholson	Data Manager
UK	Salford: Salford Royal Hosp	Prof Noel Clarke	Site PI
~~	~~~	Dr Anna Tran	Clinical/Surgical
~~	~~~	Dr Richard Cowan	Clinical/Surgical
~~	~~~	Mr Chris Betts	Clinical/Surgical
~~	~~~	Mr David Shackley	Clinical/Surgical
~~	~~~	Mr Kieran O'Flynn	Clinical/Surgical
~~	~~~	Mr Maurice Lau	Clinical/Surgical
~~	~~~	Mr Tony Elliott	Clinical/Surgical
~~	~~~	Miss Kay Goulden	Point of contact (1st)
~~	~~~	Mrs Siny George	Point of contact (1st)
~~	~~~	Ms Cellins Vinod	Point of contact (1st)
~~	~~~	Ms Catherine Redshaw	Research Nurse
~~	~~~	Ms Helen Farrell	Research Nurse
~~	~~~	Ms Rachael Allen	Research Nurse
~~	~~~	Ms Soney Dharmaprasad	Research Nurse
~~	~~~	Sister Jill Youd	Research Nurse
~~	~~~	Sister Melanie Taylor	Research Nurse
~~	~~~	Sister Sarah Kirk	Research Nurse
~~	~~~	Mr Garry Stevenson	Trial Coordinator
~~	~~~	Mrs Chloe-Anne Thompson	Data Manager
~~	~~~	Mrs Christine Farnworth	Data Manager
~~	~~~	Miss Danielle Platt	Administrator
~~	~~~	Mr Oliver Wadsworth	Administrator
~~	~~~	Ms Karen Richardson	Administrator
~~	~~~	Ms Leah Harter	Administrator
~~	~~~	Ms Malan Kaushal	Administrator
~~	~~~	Mrs Amanda Cordwell	MDT coordinator
~~	~~~	Ms Amanda Bowmer	MDT coordinator
~~	~~~	Mrs Claire Duncan (nee Keatley)	Pharmacist
~~	~~~	Ms Anne Marie Lydon	Other
UK	Salisbury: Salisbury District Hosp	Dr Adityanarayan Bhatnagar	Site PI
~~	~~~	Mr Allister Campbell	Clinical/Surgical
~~	~~~	Mr Gregor Mcintosh	Clinical/Surgical

INVESTIGATORS AND COLLABORATORS: SITE STAFF
Staff on site delegation logs

Country	Site	Names	Role
~~	~~~	Mr Mohammed El-Saghir	Clinical/Surgical
~~	~~~	Mr Peter Guy	Clinical/Surgical
~~	~~~	Mrs Lehentha Mattocks	Point of contact (1st)
~~	~~~	Mrs Catherine Reed	Research Nurse
~~	~~~	Mrs Sarah Bunn	Research Nurse
~~	~~~	Ms Julie Attlee	Research Nurse
~~	~~~	Ms Ruth Fennelly	Trial Coordinator
~~	~~~	Mrs Sophia Strong-Sheldrake	Administrator
~~	~~~	Ms Julie Gwilt	Administrator
~~	~~~	Ms Gemma Tedd	MDT coordinator
~~	~~~	Mrs Brenda Murphy	Pharmacist
UK	Scunthorpe: Scunthorpe General Hosp	Dr Sanjay Dixit	Site PI
~~	~~~	Mrs Sue Spencer	Point of contact (1st)
~~	~~~	Ms Kathleen Dent	Point of contact (1st)
~~	~~~	Ms Sandra Pearson	Point of contact (1st)
~~	~~~	Ms Dorota Potoczna	Point of contact (2nd)
~~	~~~	Mrs Karen Martin	Research Nurse
~~	~~~	Ms Jo Towse	Data Manager
~~	~~~	Ms Nikita Whotton	Data Manager
~~	~~~	Ms Marion Hood	Administrator
UK	Sheffield: Weston Park Hosp	Dr Catherine Ferguson	Site PI
~~	~~~	Dr Jessica Tay	Site PI
~~	~~~	Dr Jackie Martin	Clinical/Surgical
~~	~~~	Dr Katie Bowen	Clinical/Surgical
~~	~~~	Dr Peter Kirkbride	Clinical/Surgical
~~	~~~	Ms Rachael Clarke	Point of contact (1st)
~~	~~~	Sister Kim Wood	Research Nurse
~~	~~~	Mrs Kate Gibbins	Trial Coordinator
~~	~~~	Miss Alexandra Firth	Data Manager
~~	~~~	Mr John Martindale	Data Manager
~~	~~~	Mrs Janine Smedley (nee McCabe)	Data Manager
~~	~~~	Ms Su Clark	Data Manager
~~	~~~	Ms Catherine Spalton	Research Asst
~~	~~~	Ms Suzanne Smith	Administrator

INVESTIGATORS AND COLLABORATORS: SITE STAFF
Staff on site delegation logs

Country	Site	Names	Role
UK	Shrewsbury: Royal Shrewsbury Hosp	Dr Narayanan Srihari	Site PI
~~	~~~	Mr Ravi Prashant	Clinical/Surgical
~~	~~~	Mr Sanal Jose	Point of contact (1st)
~~	~~~	Ms Emma Bates	Point of contact (1st)
~~	~~~	Ms Indukala Chennattukungu	Point of contact (1st)
~~	~~~	Ms Joanna Clancy	Point of contact (1st)
~~	~~~	Mrs Hayley Hughes	Research Nurse
~~	~~~	Mrs Sunita Kurian-Downer	Research Nurse
~~	~~~	Ms Elena Michael	Research Nurse
~~	~~~	Ms Karen Nicholas	Research Nurse
~~	~~~	Ms Sally Potts	Research Nurse
~~	~~~	Ms Vanessa Cross	Research Nurse
~~	~~~	Sister Helen Moore	Research Nurse
~~	~~~	Mrs Marion Adams	Trial Coordinator
~~	~~~	Miss Suzanne Pope	Data Manager
~~	~~~	Mrs Sandra Smith	Data Manager
~~	~~~	Mrs Harpreet Singh	Administrator
~~	~~~	Mrs Emma Weaver	Radiographer
UK	Slough: Wexham Park Hosp	Dr Nicola Dallas	Site PI
~~	~~~	Dr Helen O'Donnell	Clinical/Surgical
~~	~~~	Dr M Laniado	Clinical/Surgical
~~	~~~	Dr Richard B Brown	Clinical/Surgical
~~	~~~	Dr Shahid Sharif	Clinical/Surgical
~~	~~~	Mr Omer Karim	Clinical/Surgical
~~	~~~	Miss Nicky Barnes	Point of contact (1st)
~~	~~~	Miss Nicole Kader	Point of contact (2nd)
~~	~~~	Ms Ana Sierra Pala	Point of contact (2nd)
~~	~~~	Mrs Victoria Robinson	Research Nurse
~~	~~~	Ms Alison Sears	Research Nurse
~~	~~~	Ms Jayne Litchfield	Research Nurse
~~	~~~	Sister Ann Jackson	Research Nurse
~~	~~~	Sister Catherine Smith	Research Nurse
~~	~~~	Mrs Julie-Ann Sinclair	Data Manager
~~	~~~	Ms Sana Mahmood	Pharmacist

INVESTIGATORS AND COLLABORATORS: SITE STAFF

Staff on site delegation logs

Country	Site	Names	Role
~~	~~~	Dr Ali Abbas	Other
UK	Somerset: Musgrove Park Hosp	Dr Emma Gray	Site PI
~~	~~~	Dr Faith McMeekin	Clinical/Surgical
~~	~~~	Dr John Boardman	Clinical/Surgical
~~	~~~	Dr John Graham	Clinical/Surgical
~~	~~~	Dr Joseph Jelski	Clinical/Surgical
~~	~~~	Dr Manivannan Periasamy	Clinical/Surgical
~~	~~~	Dr Manjusha Keni	Clinical/Surgical
~~	~~~	Dr Mary Tighe	Clinical/Surgical
~~	~~~	Dr Mohini Varughese	Clinical/Surgical
~~	~~~	Mr Surayne Segaran	Clinical/Surgical
~~	~~~	Ms Jeanette Bowes-Cavanagh	Clinical/Surgical
~~	~~~	Miss Rebecca Brown	Point of contact (1st)
~~	~~~	Mrs Sue Mahoney	Point of contact (1st)
~~	~~~	Ms Alison Whitcher	Point of contact (1st)
~~	~~~	Ms Sara Green	Point of contact (1st)
~~	~~~	Mr Tamlyn Russell	Point of contact (2nd)
~~	~~~	Mrs Maria Zietz	Point of contact (2nd)
~~	~~~	Ms Jayne Foot	Point of contact (2nd)
~~	~~~	Ms Alison Chedham	Research Nurse
~~	~~~	Ms Angela Locke	Research Nurse
~~	~~~	Ms Martha Wrigley	Research Nurse
~~	~~~	Mrs Michelle Farrar	Trial Coordinator
~~	~~~	Ms Flora Darch	Trial Coordinator
~~	~~~	Ms Christine Webster	Data Manager
~~	~~~	Mrs Sarah Wiggins	Administrator
~~	~~~	Ms Jan Ashcroft	Administrator
~~	~~~	Ms Rebecca Wallbutton	Administrator
~~	~~~	Mr Simon Goldsworthy	Radiographer
~~	~~~	Miss Odunayo Kalejaiye	Other
~~	~~~	Ms Elizabeth Ruszala	Other
UK	Southampton: Southampton General Hosp	Dr Catherine Heath	Site PI
~~	~~~	Dr Alec Paschalis	Clinical/Surgical
~~	~~~	Dr Mark Noble	Clinical/Surgical

INVESTIGATORS AND COLLABORATORS: SITE STAFF
Staff on site delegation logs

Country	Site	Names	Role
~~	~~~	Dr Victoria McFarlane	Clinical/Surgical
~~	~~~	Ms Anna Stephenson	Point of contact (1st)
~~	~~~	Ms Annelise Haskell	Point of contact (1st)
~~	~~~	Ms Fabiola Morales-Azofra	Point of contact (1st)
~~	~~~	Ms Lucy Galloway	Point of contact (1st)
~~	~~~	Ms Malavika Ganabady	Point of contact (1st)
~~	~~~	Ms Carina Mundy	Research Nurse
~~	~~~	Ms Kirsty Cumming	Research Nurse
~~	~~~	Ms Naomi James	Research Nurse
~~	~~~	Mrs Julie Kennedy	Trial Coordinator
~~	~~~	Mrs Julie Patrick	Trial Coordinator
~~	~~~	Mrs Susan Morton	Trial Coordinator
~~	~~~	Ms Shauna Wakefield	Trial Coordinator
~~	~~~	Ms Julie Abab	Administrator
~~	~~~	Ms Julie Gwilt	Administrator
~~	~~~	Ms Leanne Reader	Administrator
~~	~~~	Mrs Lisa Taylor	MDT coordinator
UK	Southend: Southend University Hosp	Dr David Tsang	Site PI
~~	~~~	Dr Abby Cyriac	Clinical/Surgical
~~	~~~	Dr Imtiaz Ahmed	Clinical/Surgical
~~	~~~	Dr Jan Prejbisz	Clinical/Surgical
~~	~~~	Dr Olivia Chan	Clinical/Surgical
~~	~~~	Mrs Tracey Davies	Point of contact (1st)
~~	~~~	Ms Sheila Reece	Research Nurse
~~	~~~	Ms Sue Bowman	Trial Coordinator
~~	~~~	Ms Heather Shires	Administrator
~~	~~~	Ms Katrina Maitland	Other
UK	Stockport: Stepping Hill Hosp	Dr John Logue	Site PI
~~	~~~	Mr WA Brough	Site PI
~~	~~~	Mr Gerald Collins	Clinical/Surgical
~~	~~~	Mr Richard Brough	Clinical/Surgical
~~	~~~	Mr Elliott Wiss	Point of contact (1st)
~~	~~~	Ms Sarah Connolly nee McKenna	Point of contact (1st)
~~	~~~	Ms Sarah Smallwood	Point of contact (1st)

INVESTIGATORS AND COLLABORATORS: SITE STAFF
Staff on site delegation logs

Country	Site	Names	Role
~~	~~~	Ms Sheila Hodgkinson	Point of contact (1st)
~~	~~~	Mrs Tricia Coughlan	Point of contact (2nd)
~~	~~~	Ms Emma Goodwin	Point of contact (2nd)
~~	~~~	Mrs Helen Haydock	Research Nurse
~~	~~~	Ms Christina Gilmour	Research Nurse
~~	~~~	Ms Eleanor Anscombe	Research Nurse
~~	~~~	Ms Sam Corcoran	Research Nurse
~~	~~~	Ms Jill Taylor	Data Manager
~~	~~~	Ms Tracie Cocks	Data Manager
~~	~~~	Ms Pat Clitheroe	Administrator
~~	~~~	Mrs Carol Rotherham	MDT coordinator
~~	~~~	Mr John Kilmartin	Pharmacist
~~	~~~	Ms Lucy Orrell	Pharmacist
~~	~~~	Ms Susan Graham	Pharmacist
~~	~~~	Dr Umi Hatimy	Other
UK	Stoke: Royal Stoke University Hosp	Dr Salil Vengalil	Site PI
~~	~~~	Dr Fawzi Adab	Clinical/Surgical
~~	~~~	Dr Kathirvelu Dhinakaran	Clinical/Surgical
~~	~~~	Dr Rajanee Bhana	Clinical/Surgical
~~	~~~	Mrs Angela Peake	Point of contact (1st)
~~	~~~	Ms Georgia Thomasson	Point of contact (1st)
~~	~~~	Miss Elizabeth Sellars	Point of contact (2nd)
~~	~~~	Ms Katrina Parkinson	Point of contact (2nd)
~~	~~~	Mrs Angela Ward	Research Nurse
~~	~~~	Mrs Marion Evans	Research Nurse
~~	~~~	Ms Alison Myatt	Research Nurse
~~	~~~	Ms Jenny Walton	Research Nurse
~~	~~~	Ms Julie Storer	Data Manager
~~	~~~	Ms Rowena Smith	Radiographer
UK	Sutton-in-Ashfield: King's Mill Hosp	Dr Eliot Chadwick	Site PI
~~	~~~	Dr Alastair McCabe	Clinical/Surgical
~~	~~~	Dr Daniel Saunders	Clinical/Surgical
~~	~~~	Dr Georgina Walker	Clinical/Surgical
~~	~~~	Dr Jun Lim	Clinical/Surgical

INVESTIGATORS AND COLLABORATORS: SITE STAFF
Staff on site delegation logs

Country	Site	Names	Role
~~	~~~	Dr Santhanam Sundar	Clinical/Surgical
~~	~~~	Mr Dominic Nash	Point of contact (1st)
~~	~~~	Ms Susan Smith	Point of contact (1st)
~~	~~~	Miss Jamie-Rae Burgoyne	Data Manager
~~	~~~	Mr Steve Haigh	Pharmacist
~~	~~~	Mrs Samantha Boam	Pharmacist
~~	~~~	Ms Keri Hollis	Pharmacist
~~	~~~	Ms Lyndsey Munson	Pharmacist
~~	~~~	Ms Lynne Wade	Pharmacist
~~	~~~	Dr Shafiq Gill	Other
UK	Swansea: Singleton Hosp	Dr Mau-Don Phan	Site PI
~~	~~~	Dr Delia Pudney	Clinical/Surgical
~~	~~~	Miss Ellen Tait	Point of contact (1st)
~~	~~~	Miss Nicola Lemon	Point of contact (1st)
~~	~~~	Ms Bethan Williams	Point of contact (1st)
~~	~~~	Ms Emily Harris (n. Marchant)	Point of contact (1st)
~~	~~~	Ms Elizabeth Evans	Point of contact (2nd)
~~	~~~	Ms Maria Johnstone	Point of contact (2nd)
~~	~~~	Mrs Alex Franklin	Research Nurse
~~	~~~	Ms Alison Stretch	Research Nurse
~~	~~~	Mr Lewis Jones	Trial Coordinator
~~	~~~	Ms Rachael Spence	Radiographer
UK	Swindon: Great Western Hosp	Dr Omar Khan	Site PI
~~	~~~	Dr David J Cole	Clinical/Surgical
~~	~~~	Dr Shiroma De Silva-Minor	Clinical/Surgical
~~	~~~	Ms Suzannah Pegler	Point of contact (1st)
~~	~~~	Ms Abbie Poole	Point of contact (2nd)
~~	~~~	Mr Vivian Zinyemba	Research Nurse
~~	~~~	Mrs Debbie Palmer	Research Nurse
~~	~~~	Ms Ania Jones	Research Nurse
~~	~~~	Ms Cerila Parajes	Research Nurse
~~	~~~	Ms Ellen Starling	Research Nurse
~~	~~~	Sister Helen Winter	Research Nurse
~~	~~~	Sister Jan Dodge	Research Nurse

INVESTIGATORS AND COLLABORATORS: SITE STAFF
Staff on site delegation logs

Country	Site	Names	Role
~~	~~~	Sister Tracey Sargent	Research Nurse
~~	~~~	Mrs Sarah Grayland	Research Asst
~~	~~~	Mr Tim Owen	Administrator
~~	~~~	Mrs Rebecca Belcher	Administrator
~~	~~~	Ms Becky Taylor	Administrator
UK	Torbay: Torbay District General Hosp	Dr Anna Lydon	Site PI
~~	~~~	Dr Erica Watts	Clinical/Surgical
~~	~~~	Dr Jorg Michels	Clinical/Surgical
~~	~~~	Dr Rajaguru Srinivasan	Clinical/Surgical
~~	~~~	Mr Jon Buckley	Point of contact (1st)
~~	~~~	Mrs Michele Allison	Point of contact (1st)
~~	~~~	Mrs Shelley Chamberlain	Point of contact (1st)
~~	~~~	Miss Linda Welsh	Point of contact (2nd)
~~	~~~	Mrs Donna Cuffe	Research Nurse
~~	~~~	Mrs Elaine Vandecandalaere	Research Nurse
~~	~~~	Ms Catherine Brookman	Research Nurse
~~	~~~	Ms Helen Greedus	Research Nurse
~~	~~~	Ms Ingrid Koehler	Research Nurse
~~	~~~	Ms Lorraine Thornton	Research Nurse
~~	~~~	Miss Claire Fairfax	Administrator
~~	~~~	Miss Hannah Griffin	Administrator
~~	~~~	Miss Stacey Davies	Administrator
~~	~~~	Ms Julia Pym	Administrator
~~	~~~	Mr Martyn Blundell	Pharmacist
~~	~~~	Dr Fiona Roberts	Other
UK	Wakefield: Pinderfields Hosp	Dr Juliette Anderson	Site PI
~~	~~~	Dr Ann Henry	Clinical/Surgical
~~	~~~	Dr Catherine Coyle	Clinical/Surgical
~~	~~~	Dr Chris Fosker	Clinical/Surgical
~~	~~~	Dr Nathalie Casanova	Clinical/Surgical
~~	~~~	Dr Peter Dickinson	Clinical/Surgical
~~	~~~	Dr Sree Rodda	Clinical/Surgical
~~	~~~	Mr Philip Weston	Clinical/Surgical
~~	~~~	Mr Rohit Chahal	Clinical/Surgical

INVESTIGATORS AND COLLABORATORS: SITE STAFF

Staff on site delegation logs

Country	Site	Names	Role
~~	~~~	Mr Subramanian Kanaga Sundaram	Clinical/Surgical
~~	~~~	Mr Subramanian Kanaga-Sundaram	Clinical/Surgical
~~	~~~	Mr Jim Anderson	Point of contact (1st)
~~	~~~	Mr Stephen Littler	Point of contact (1st)
~~	~~~	Mr Jonathan Slack	Point of contact (2nd)
~~	~~~	Ms Beverley Taylor	Point of contact (2nd)
~~	~~~	Ms Barbara Burlace	Research Nurse
~~	~~~	Ms Janine Heeley	Administrator
~~	~~~	Ms Julie Ball	Administrator
~~	~~~	Mr Richard Bowers	Pharmacist
~~	~~~	Ms Louise Benton	Pharmacist
UK	Warwick: Warwick Hosp	Dr Andrew Chan	Site PI
~~	~~~	Dr Andrew Stockdale	Clinical/Surgical
~~	~~~	Mr Ashley Johnson-Rollings	Point of contact (1st)
~~	~~~	Mrs Tina Gamble	Point of contact (1st)
~~	~~~	Ms Donna Walsh	Point of contact (1st)
~~	~~~	Ms Jo Williams	Point of contact (2nd)
~~	~~~	Mrs Elaine Simmons	Research Nurse
~~	~~~	Mrs Helen Millage	Research Nurse
~~	~~~	Ms Eilish O’Neill	Research Nurse
~~	~~~	Ms Kerrie Webb	Research Nurse
~~	~~~	Ms Lyn Hartwell	Research Nurse
~~	~~~	Mrs Theresa Griffiths	Data Manager
~~	~~~	Mrs Julia Jones	Pharmacist
~~	~~~	Ms Judith Chettle	Pharmacist
UK	Weston-Super-Mare: Weston General Hosp	Dr Serena Hilman	Site PI
~~	~~~	Dr Symeon Eleftheriadis	Clinical/Surgical
~~	~~~	Dr Tom Wells	Clinical/Surgical
~~	~~~	Mr Harvey Dymond	Point of contact (1st)
~~	~~~	Mr Hugh Lloyd-Jones	Research Nurse
~~	~~~	Mr John Anderson	Administrator
UK	Wirral: Clatterbridge Centre for Oncology	Dr Isabel Syndikus	Site PI
~~	~~~	Dr Helen Innes	Clinical/Surgical
~~	~~~	Dr John Littler	Clinical/Surgical

INVESTIGATORS AND COLLABORATORS: SITE STAFF
Staff on site delegation logs

Country	Site	Names	Role
~~	~~~	Dr Shaun Tolan	Clinical/Surgical
~~	~~~	Mrs Kathryn Hughes	Point of contact (1st)
~~	~~~	Mrs Sarah Bennett	Point of contact (1st)
~~	~~~	Mrs Sharon Dunn	Point of contact (1st)
~~	~~~	Ms Alison Kelly	Point of contact (1st)
~~	~~~	Ms Nikki Miller	Point of contact (1st)
~~	~~~	Mr Matthew Stott	Research Nurse
~~	~~~	Mrs Diane Fildes	Research Nurse
~~	~~~	Ms Alison Hassall	Research Nurse
~~	~~~	Ms Gaynor Herbert	Research Nurse
~~	~~~	Mr Laurie Lomax	Trial Coordinator
~~	~~~	Mr Chris Nutman	Data Manager
~~	~~~	Ms Alison Weston	Data Manager
UK	Wolverhampton: New Cross Hosp	Dr Ian Sayers	Site PI
~~	~~~	Dr Ali Samanci	Clinical/Surgical
~~	~~~	Dr Mark Churn	Clinical/Surgical
~~	~~~	Dr Pek Keng-Koh	Clinical/Surgical
~~	~~~	Mr Peter Cooke	Clinical/Surgical
~~	~~~	Arizoo Mohseeni	Point of contact (1st)
~~	~~~	Miss Renita Pawaroo	Point of contact (1st)
~~	~~~	Mr David Homer	Point of contact (1st)
~~	~~~	Mr Jason Rogers	Point of contact (2nd)
~~	~~~	Mrs Christine Kirk	Research Nurse
~~	~~~	Mrs Claire Lomas	Research Nurse
~~	~~~	Mrs Emma Sharman	Research Nurse
~~	~~~	Mrs Ivanna Baker	Research Nurse
~~	~~~	Ms Anna Grant	Trial Coordinator
~~	~~~	Mrs Hazel Spencer	Data Manager
~~	~~~	Ms Debbie Spruce	Data Manager
~~	~~~	Mrs Liz Radford	Research Asst
~~	~~~	Ms Hannah Worthington	Research Asst
~~	~~~	Ms Marian McCormick	Research Asst
~~	~~~	Ms Jenny Chatfield	Administrator
~~	~~~	Ms Vanda Carter	Other

INVESTIGATORS AND COLLABORATORS: SITE STAFF
Staff on site delegation logs

Country	Site	Names	Role
UK	Worcester: Worcestershire Royal Hosp	Dr Lisa Capaldi	Site PI
~~	~~~	Dr Jo Bowen	Clinical/Surgical
~~	~~~	Dr Kamalnayan Gupta	Clinical/Surgical
~~	~~~	Mr Jacob Taylor	Point of contact (1st)
~~	~~~	Mrs Amanda Holdsworth	Point of contact (1st)
~~	~~~	Ms Jayne Tyler	Point of contact (1st)
~~	~~~	Mrs Dagmara Bak	Point of contact (2nd)
~~	~~~	Mrs Hayley Hodson	Point of contact (2nd)
~~	~~~	Mrs Kristy Cleary	Research Nurse
~~	~~~	Mrs Helen Tranter	Trial Coordinator
~~	~~~	Ms Janet Forkes	Trial Coordinator
~~	~~~	Mrs Patricia Rimell	Data Manager
~~	~~~	Ms Sue Davies	Data Manager
~~	~~~	Miss Kate Ledger	Research Asst
~~	~~~	Ms Jennifer Healey-Mariano	Research Asst
~~	~~~	Mr Hugh Morrow	Pharmacist
~~	~~~	Mrs Ann White	Pharmacist
~~	~~~	Mrs Monica Gauntlett	Pharmacist
~~	~~~	Ms Alison Rosoman	Pharmacist
~~	~~~	Ms Heather Perry	Pharmacist
UK	Worthing: Worthing Hosp	Dr Ashok Nikapota	Site PI
~~	~~~	Dr David Bloomfield	Clinical/Surgical
~~	~~~	Dr George Plataniotis	Clinical/Surgical
~~	~~~	Miss Raquel Gomez-Marcos	Point of contact (1st)
~~	~~~	Ms Chloe Hoskins	Point of contact (1st)
~~	~~~	Ms Marian Flynn-Batham	Point of contact (1st)
~~	~~~	Ms Sarah Rippin	Point of contact (1st)
~~	~~~	Mr Tan Tsawayo	Point of contact (2nd)
~~	~~~	Mrs Sarah Funnell	Point of contact (2nd)
~~	~~~	Miss Paula Wakelen	Research Nurse
~~	~~~	Ms Dawn Crowe (nee Hughes)	Research Nurse
~~	~~~	Ms Jeanette Gilbert	Research Nurse
~~	~~~	Ms Linda Folkes	Research Nurse
~~	~~~	Ms Nikki Turner	Research Nurse

INVESTIGATORS AND COLLABORATORS: SITE STAFF
Staff on site delegation logs

Country	Site	Names	Role
~~	~~~	Ms Sally Moore	Research Nurse
~~	~~~	Mr Matthew Smith	Trial Coordinator
~~	~~~	Ms Celia Gonzalez	Pharmacist
UK	Wycombe: Wycombe Hosp	Dr Katherine Hyde	Site PI
~~	~~~	Dr Ami Sabharwal	Clinical/Surgical
~~	~~~	Dr Andrew Weaver	Clinical/Surgical
~~	~~~	Dr Joanne Brady	Clinical/Surgical
~~	~~~	Dr Niki Panakis	Clinical/Surgical
~~	~~~	Dr Philip Camilleri	Clinical/Surgical
~~	~~~	Dr Thinn Pwint	Clinical/Surgical
~~	~~~	Prof Andrew Protheroe	Clinical/Surgical
~~	~~~	Dr Janice Carpenter	Point of contact (1st)
~~	~~~	Mrs Anita Cserbane	Point of contact (1st)
~~	~~~	Mrs Alice Ngumo	Point of contact (2nd)
~~	~~~	Ms Emma Hogbin	Research Nurse
~~	~~~	Ms Hazel Wynn	Research Nurse
~~	~~~	Ms Sarah Manyangadze	Research Nurse
~~	~~~	Mrs Manisha Joshi	Data Manager
~~	~~~	Miss Claire Fernandez	Research Asst
~~	~~~	Ms Susan McLain-Smith	Research Asst
~~	~~~	Mr Rahul Kurup	Administrator
~~	~~~	Mr Neil Trew-Smith	Pharmacist
~~	~~~	Ms Roisin Kavanagh	Pharmacist
~~	~~~	Mr Neil Trewsmith	Other
UK	Yeovil: Yeovil District Hosp	Mr Tim Porter	Site PI
~~	~~~	Dr Emma Gray	Clinical/Surgical
~~	~~~	Dr Erica Beaumont	Clinical/Surgical
~~	~~~	Dr Geoffrey Sparrow	Clinical/Surgical
~~	~~~	Dr Shiyam Kumar	Clinical/Surgical
~~	~~~	Mrs Joanna Allison	Point of contact (1st)
~~	~~~	Mrs Joanne Macrory	Point of contact (1st)
~~	~~~	Mrs Kerry Rennie	Point of contact (1st)
~~	~~~	Miss Jess Perry	Point of contact (2nd)
~~	~~~	Mr Nigel Beer	Research Nurse

INVESTIGATORS AND COLLABORATORS: SITE STAFF
Staff on site delegation logs

Country	Site	Names	Role
~~	~~~	Mrs Lucy Pippard	Research Nurse
~~	~~~	Ms Shirley Fox	Research Nurse
~~	~~~	Ms Barbara Williams-Yesson	Trial Coordinator
~~	~~~	Miss Kate Beesley	Administrator
~~	~~~	Mrs Michelle Kotze	Administrator
~~	~~~	Miss Claire Barron	Pharmacist
~~	~~~	Mr David Donaldson	Pharmacist
~~	~~~	Mrs Tracey Duckett	Pharmacist
UK	York: York District Hosp	Dr Joji Joseph	Site PI
~~	~~~	Dr David Bottomley	Clinical/Surgical
~~	~~~	Dr Hima Bindu Musunuru	Clinical/Surgical
~~	~~~	Dr Russ Wilson	Clinical/Surgical
~~	~~~	Mr Michael Stower	Clinical/Surgical
~~	~~~	Mr Paul Brittain	Point of contact (1st)
~~	~~~	Mrs Flor Davies	Point of contact (1st)
~~	~~~	Mrs Paula Strider	Point of contact (1st)
~~	~~~	Mrs Claire Brookes	Point of contact (2nd)
~~	~~~	Mr Mark Fearnley	Research Nurse
~~	~~~	Mrs Jane Balderson	Research Nurse
~~	~~~	Mrs Jo Ingham	Research Nurse
~~	~~~	Mrs Lisa Mole	Research Nurse
~~	~~~	Mrs Nora Youngs	Research Nurse
~~	~~~	Ms Carol Taylor	Research Nurse
~~	~~~	Ms Emma Giddings	Research Nurse
~~	~~~	Ms Fereshteh Fallah	Research Nurse
~~	~~~	Ms Hilary Campbell	Research Nurse
~~	~~~	Ms Joanna Todd	Research Nurse
~~	~~~	Ms Lucy Howard	Research Nurse
~~	~~~	Mr Andy Holbrook	Trial Coordinator
~~	~~~	Mrs Srilakshmi Gollapothu	Data Manager
~~	~~~	Miss Shamila Saleem	Research Asst
~~	~~~	Mr Louis Thackray	Research Asst
~~	~~~	Ms Monica Campos	Research Asst
~~	~~~	Miss Lorna Bowling	Pharmacist

INVESTIGATORS AND COLLABORATORS: SITE STAFF

Staff on site delegation logs

Country	Site	Names	Role
~~	~~~	Mr John Wightman	Pharmacist
~~	~~~	Mr Mark Elliott	Pharmacist
~~	~~~	Mr Richard Evans	Pharmacist
~~	~~~	Mrs Christine Jakeman	Pharmacist
~~	~~~	Ms Michelle Donnison	Pharmacist
~~	~~~	Ms Rachel Conway	Pharmacist
~~	~~~	Ms Sally Harvey	Pharmacist
~~	~~~	Ms Sarah Pashley	Other

PARTICIPANTS

Approximately 4,000 people chose to participate in RADICALS. In addition to their care teams, they have been supported by family, friends and other key people. Every person who has participated in the trial is appreciated by the trial team and should be appreciated by the wider public. The findings from clinical trials can change practice for the future, but clinical trials only happen because people find the time and make the effort to support them. Thank you.
